# Uniform design-embedded predictions of (tetra-)peptide physicochemical properties

**DOI:** 10.1093/bioinformatics/btag036

**Published:** 2026-01-19

**Authors:** Zhihui Zhu, Huapeng Liu, Xuechen Li, Haojin Zhou, Jiaqi Wang

**Affiliations:** ZJU-Hangzhou Global Scientific and Technological Innovation Center, Zhejiang University, Hangzhou, Zhejiang 311215, China; Wisdom Lake Academy of Pharmacy, Xi’an Jiaotong-Liverpool University, Suzhou, Jiangsu 215123, China; Department of Chemistry, State Key Laboratory of Synthetic Chemistry, The University of Hong Kong, Pokfulam, Hong Kong SAR 999077, China; Wisdom Lake Academy of Pharmacy, Xi’an Jiaotong-Liverpool University, Suzhou, Jiangsu 215123, China; Department of Chemistry, State Key Laboratory of Synthetic Chemistry, The University of Hong Kong, Pokfulam, Hong Kong SAR 999077, China

## Abstract

**Motivation:**

Short peptides hold significant promise in drug discovery and materials science due to their biocompatibility, multifunctionality, ease of synthesis, *etc*. However, accurately predicting their physicochemical properties, a prerequisite for application development, remains a grand challenge due to the sheet quantity of peptides.

**Results:**

This study presents an innovative approach integrating uniform design (UD) on the sampling over the whole space with artificial intelligence (AI) on the sampled data to enhance prediction of key physicochemical properties, including aggregation propensity (AP), hydrophilicity (logP), and isoelectric point (pI), within the complete sequence space of tetrapeptides (160 000 sequences). Using UD, we generate 31 distinct peptide datasets, with a consistent amino acid occupation fraction of 5% at each position, thereby creating unbiased training data without any amino acid preferences for training AI models. This work provides comprehensive datasets on the physicochemical properties of all tetrapeptides, develops robust AI-based predictive models, and quantitatively elucidates the relationships between key physicochemical attributes and self-assembly behaviors of short peptides by Shapley Additive Explanations (SHAP) analysis. By integrating the strategic experimental design (i.e. UD), AI modeling, and peptide domain knowledge, our approach facilitates the discovery and optimization of functional peptides, offering new opportunities for peptide-based therapeutic applications.

**Availability and implementation:**

The complete datasets, source code, and pretrained models are made available at the Github repository (https://github.com/JiaqiBenWang/UD-AI-Peptide) and Zenodo (https://doi.org/10.5281/zenodo.17984124).

## Graphical abstract

**Figure:**
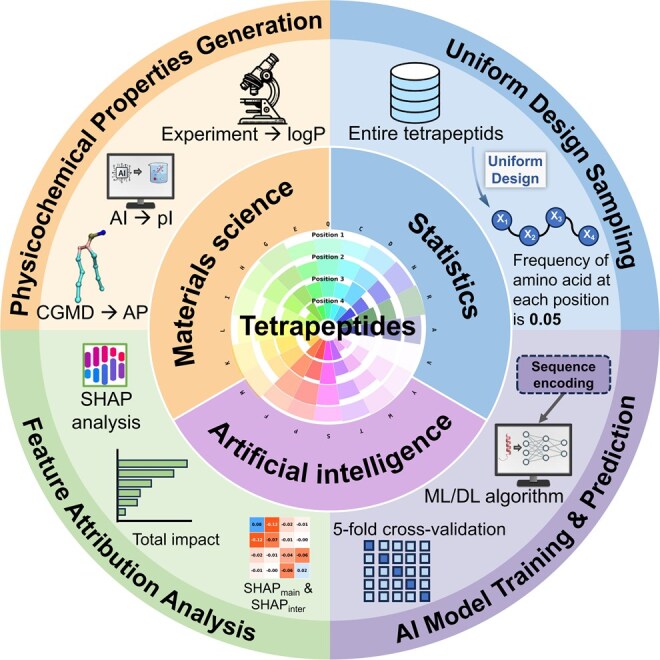

## 1 Introduction

Peptides, essential biomolecules, have attracted considerable interest in both academia and industry due to their biocompatibility (Matson *et al.* 2011), multifunctionality (Levin *et al.* 2020), and therapeutic potential (Acar *et al.* 2017). Their ability to self-assemble into functional nanostructures has further expanded their applications across diverse fields, including drug delivery, tissue engineering, bioimaging, antimicrobial therapy, and more (Cao *et al.* 2024). In drug delivery, self-assembled peptide nanostructures serve as efficient carriers for therapeutic agents, enhancing efficacy while minimizing side effects (Vilaça *et al.* 2017, Yang *et al.* 2021). In tissue engineering, they function as bioscaffolds that promote cell proliferation and tissue regeneration (Lee *et al.* 2009, Loo *et al.* 2014). Additionally, peptide self-assemblies can be engineered for noninvasive *in vivo* bioimaging, enabling real-time visualization of biological processes (An *et al.* 2020). Peptides also exhibit antimicrobial properties, offering novel strategies against antibiotic-resistant bacteria (Li *et al.* 2016). Despite these diverse applications, a major challenge remains in accurately predicting peptide self-assembly behavior and elucidating its intrinsic correlations with sequence-dependent physicochemical properties.

In recent years, the integration of artificial intelligence (AI, all abbreviations and acronyms are listed in the Supplementary file of “List of Abbreviations and Acronyms”, available as supplementary data at *Bioinformatics* online) with molecular dynamics (MD) simulations has emerged as a prominent approach for predicting the self-assembly behavior of peptides (Zhou *et al.* 2022, Xu *et al.* 2023, Cao *et al.* 2024, Njirjak *et al.* 2024, Wang *et al.* 2024). MD simulations generate high-quality data under consistent conditions, which can be leveraged as training datasets to develop AI models for predicting self-assembly sequences. For example, coarse-grained MD (CGMD) has been coupled with AI model to identify aggregation-prone sequences in large peptide spaces, such as pentapeptides and decapeptides, using search algorithms, transformer-based deep learning, or generative models (Batra *et al.* 2022, Wang *et al.* 2023, Chen *et al.* 2024). These approaches represent a powerful synergy between advanced molecular simulations and AI-driven prediction to accelerate peptide discovery.

However, the sampling methods and the representativeness of the training data in the aforementioned studies have not been sufficiently justified. It is important to note that the accuracy of AI models, not only those predicting peptide properties, is highly dependent on the quality and representativeness of the training data (Whang *et al.* 2023, Yan *et al.* 2025). As peptide length increases, the diversity of possible sequences within the complete sequence space grows exponentially, incurring substantial challenges for accurate predictions. Specifically, the training data may fail to adequately capture the intricate interplay between peptide sequences and their various physicochemical properties (Whang *et al.* 2023). Inadequate sampling of training data can introduce bias toward specific amino acid sequences or properties, thereby limiting the model’s generalization ability. Consequently, the implementation of a robust experimental design, which has been largely overlooked in previous research, is essential for enhancing the generalization and accuracy of AI models in predicting the physicochemical properties of peptides.

**Figure 1:**
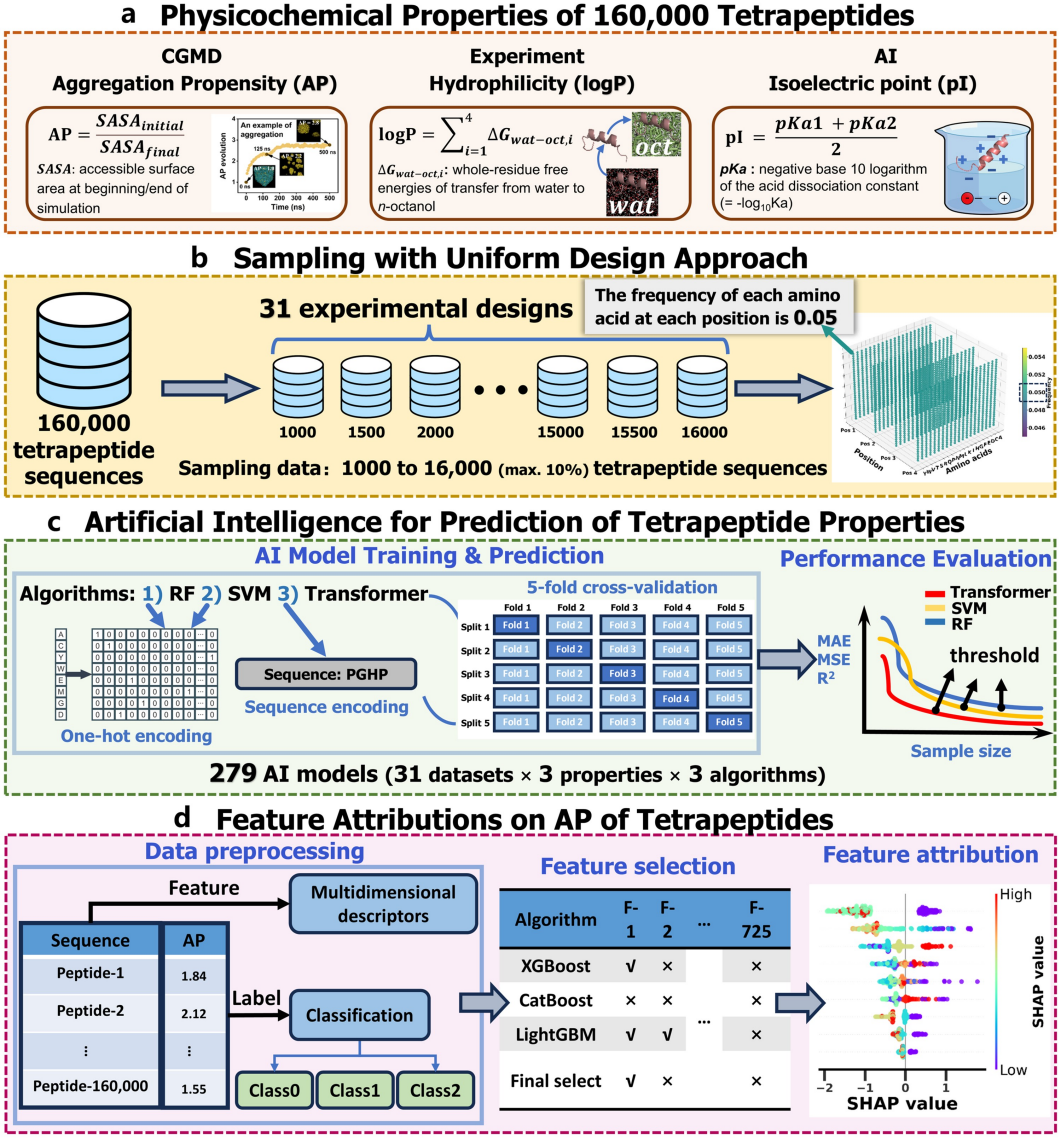
Workflow of integrated UD and AI framework for predicting the physicochemical properties of tetrapeptides. (a) Generation of physicochemical properties for 160 000 tetrapeptides, including AP, logP, and pI, using CGMD simulations, experimental methods, and AI predictions, respectively. (b) Sampling of training datasets using the UD approach, ensuring that the frequency of each amino acid at each position is 0.05. This process results in 31 experimental design datasets, with sample size incrementing by 500 from 1000 to 16 000. (c) Training of AI model to predict tetrapeptide properties (AP, logP, and pI) using RF, SVM, and transformer models. For RF and SVM models, tetrapeptide sequences are preprocessed using one-hot encoding, while the transformer model employs inputs of peptide sequences directly. Five-folds cross-validation is embedded during training. Model performance is evaluated using metrics of MAE, MSE, and *R*^2^. Learning curve analysis is conducted to assess data utilization efficiency and identify performance inflection points. (d) Feature attribution analysis for the AP of tetrapeptides. Multidimensional descriptors are extracted as features, and labels are classified into three categories to build interpretative models. SHAP analysis is adopted to elucidate the contribution of individual features to the predictive outcome, thereby enhancing model interpretability and identifying critical factors influencing tetrapeptide self-assembly behavior.

This study introduces an innovative methodology that integrates uniform design (UD) and AI to optimize predictive models for tetrapeptide properties (Fig. 1). By strategically selecting peptide sequences, we aim to ensure that the data used for training AI models adequately represents the overall distribution of the complete peptide sequence space. To achieve this, 31 sets of UD samples are generated, maintaining an amino acid occupation frequency of 0.05 (equivalent to 1/20) at each position, thereby providing balanced and representative training data for AI models, including random forest (RF) (Ho, 1998), support vector machine (SVM) (Hearst *et al.* 1998), and transformer models (Vaswani *et al.* 2017). The performance of the AI models is evaluated using both fixed and nonfixed testing datasets (see Section 2.4), and the optimal sample size for predicting each physicochemical property, including aggregation propensity (AP), hydrophilicity (logP), and isoelectric point (pI), is determined.

To gain deeper insights into the factors influencing the aggregation behavior of tetrapeptides, this study conducts an extensive analysis of the AP values of 160 000 tetrapeptide sequences. We employed tree-based machine learning (ML) models, including XGBoost (Chen and Guestrin, 2016), LightGBM (Ke *et al.* 2017), and CatBoost (Prokhorenkova *et al.* 2018), in conjunction with Shapley Additive Explanations (SHAP) analysis (Lundberg and Lee, 2017; Hsueh *et al.* 2023; Štrumbelj and Kononenko, 2014) to identify the critical physicochemical characteristics and their interactions that affect the AP of tetrapeptides. The insights derived from these analyses may potentially be extrapolated to peptides of varying lengths, thereby enhancing the experimental design of self-assembling peptides across extended sequence space.

In summary, this research generates high-quality physicochemical property data for all 160,000 tetrapeptides (Supplementary Data 1, available at Github or Zenodo repository) and develops accurate predictive models. Through the proposed integration of UD and AI, this work provides valuable insights into the relationship between physicochemical properties and aggregation behaviors. The selected tetrapeptide samples form a solid foundation for future AI-driven predictions of additional properties, such as peptide-protein binding affinities, enzymatic selectivity, and electronic band gap, etc., holding significant potential for applications in peptide drug developments, catalysts, and semiconductor technologies.

## 2 Materials and methods

### 2.1 A mathematical framework

In this section, we set up a mathematical framework for the problem and present some key results that lead to the analysis methods, which, in turn, promote the deployment of UD in AI-related research.

Consider the problems of evaluating a *k*-peptide (*k* indicates the length of the peptide, i.e. the number of amino acids in the peptide chain) based on its 2D sequence, $S=(s_{1},\ldots ,s_{k})\in S$, where $s_{i}$ can only take the value of an amino acid out of q=20 in total, and assume one is interested in one of the peptide’s physicochemical properties at a given condition, as y∈Y. The goal of the peptide research is to derive the function f:|S→Y,y=f(s). However, due to the natural process of deriving those properties via computations (MD simulations or AI predictions), random errors will be produced during the process. Without loss of generality, the model can be represented as y=f(s)+ϵ, where ϵ is a random error with mean 0 and unknown variance $\sigma^{2}$.

Naturally, one could go through all the *k*-peptides ($N=q^{k}$, *N* is the total number of peptides of the same length *k*) and thus the function *f* can be fully known. However, it is unpractical due to the enormous sequence space, especially when the *k* is larger than 3 (i.e. *N* $>20^{3}=8000$). Therefore, a practical problem arises: how can one select points from all the possible *k*-peptides and approximate the function *f*? Such a problem can be decomposed into the following:

1. What sampling method should be used?
2. How many sample points *n* or what sampling fraction sf=n/N are required?
3. Which analysis method is appropriate?
4. If the chosen method produces an overly complex model, how can it be simplified without losing predictive power?

For Question 1, we adopted UD, a method developed by Fan (1980) to handle high-cost experiments where even orthogonal designs (requiring $q^{2}$ samples) are infeasible. UD uses number-theoretic techniques to approximate a uniform distribution in the unit hypercube, with equal frequency of each amino acid (level) across samples. Its uniformity is formalized as:

Lemma 1 (The uniformity of a uniform design). Let $X={\left[0,1\right]}^{s}$ be the s-dimensional unit hypercube and $P=\{x_{1},\ldots ,x_{n}\}$ as a set of n design points in X. Then a uniform design constructed using Good Lattice Points will converge to the uniform distribution in ${\left[0,1\right]}^{s}$.

Subsequent development on the UD lead to optimality under a discrepancy measure as it is not unique.

Lastly, we established the optimality of UD in this study, under the mean square error, $E[{\left(\hat{f}(x|X(D),Y(D))-Y|X=x\right)}^{2}]$ with X(D),Y(D) being the design matrix and observed responses from the Design *D*.

Theorem 1 (The optimality of the uniform design). *For a consistent estimator* $\hat{f}$*, if the function f is totally unknown and n is sufficiently large, then the uniform design will yield the predictor with the lowest MSE among all possible designs.*

Note that *f* needs to be assumed to be totally unknown. Otherwise, suppose *f* can be represented as g°h, where *g* is totally unknown and *h* is known. Then following the similar approach in the above proof, one can show that $h^{-1}(D)$ is asymptotically better than *D* in MSE.

Theorem 1 indicates that for a *de novo* design, to achieve a certain level of prediction accuracy, UD requires the minimal sample size, among all possible sampling methods. Meanwhile, if there is prior information, UD can also be adapted to it by considering an inverse transformation promoted by the prior information.

For Questions 2–4, detailed discussions and corresponding answers are presented in the “Results and Discussion” section. And more prove of theories can be found in the Supplementary Section 3.1, available as supplementary data at *Bioinformatics* online.

### 2.2 Calculations of physicochemical properties

#### 2.2.1 Aggregation propensity—AP

The AP values of tetrapeptides are calculated based on the results of CGMD simulations, performed using the open-source package GROMACS (Abraham *et al.* 2015) and the Martini force field (version 2.2) (Marrink et al. 2007, Monticelli *et al.* 2008, De Jong *et al.* 2013). The formula for AP is defined as


(1) $$AP=\frac{\mathrm{SAS}A_{\text{initial}}}{\mathrm{SAS}A_{\text{final}}}$$

Here, $\mathrm{SAS}A_{\text{initial}}$ and $\mathrm{SAS}A_{\text{final}}$ denote the solvent-accessible surface area of tetrapeptides in the simulation box at the beginning and end of the CGMD simulation, respectively (Batra *et al.* 2022). For nonaggregating tetrapeptides, the AP values remain at 1, while for aggregating peptides, the AP values will gradually increase over 1 (Liu *et al.* 2023). According to prior research (Liu *et al.* 2023, Wang *et al.* 2024), an AP value ranging from 1.5 to 2 signifies favorable aggregation, facilitating optimal self-assembly behavior. However, when the AP value exceeds 2, the tetrapeptides may exhibit excessive aggregation, resulting in precipitation. Details of CGMD simulation can be found in the Supplementary Section 3.2, available as supplementary data at *Bioinformatics* online.

#### 2.2.2 Hydrophilicity—logP

The logP value of peptides reflects hydrophilicity, indicating their tendency to partition between octanol and water. Hydrophilic peptides (larger logP values) readily interact with water molecules through hydrogen bonding, whereas hydrophobic peptides (smaller logP values) tend to avoid such interactions (Monticelli *et al.* 2008, Frederix *et al.* 2011). The hydrophilicities of 160 000 tetrapeptide sequences are experimentally determined and calculated using the following equation:


(2) $$\mathrm{logP}=\sum_{i=1}^{4}\Delta G_{\text{wat-oct},i}$$

In this equation, $\Delta G_{\text{wat-oct},i}$ (unit: kcal/mol) represents the Wimley–White whole-residue hydrophilicity of each amino acid (Wimley and White 1996, White and Wimley 1998), corresponding to the free energy change associated with transferring an amino acid from the aqueous phase to *n*-octanol phase.

If $\Delta G_{\text{wat-oct},i}>0$, the amino acid is more stable in the aqueous phase than in the *n*-octanol phase, indicating hydrophilicity and favoring interactions with water molecules. Conversely, if $\Delta G_{\text{wat-oct},i}<0$, the amino acid is hydrophobic and prefers to avoid interactions with water.

#### 2.2.3 Isoelectric point

The pI (Righetti 2000) of a peptide refers to the pH value at which the net charge of the peptide molecule is zero, resulting from the balance between positively charged basic amino acids and negatively charged acidic amino acids. This value is calculated using an online isoelectric point calculator 2.0 (Kozlowski 2021), which utilizes deep learning to predict the pI and pKa values of proteins and peptides. This tool can be accessed at http://www.ipc2-isoelectric-point.org.

### 2.3 Sampling with UD

We sampled subsets within the tetrapeptide sequence space using the UD method (Fan 1980). This approach is a type of space-filling design that aims to select experimental points that are uniformly distributed over the entire experimental domain (Fang *et al.* 2000, 2005). Our experimental domain, χ, is defined as the Cartesian product of four factors (A, B, C, D), with each factor representing a position in a tetrapeptide sequence at the level of 20 natural amino acids. Specifically, χ=$\left\{A_{1},A_{2},\ldots ,A_{20}\right\}$  ×  $\left\{B_{1},B_{2},\ldots ,B_{20}\right\}$  ×  $\left\{C_{1},C_{2},\ldots ,C_{20}\right\}$  ×  $\left\{D_{1},D_{2},\ldots ,D_{20}\right\}$, resulting in a sequence space of 160 000 possible combinations of tetrapeptides.

In this space, each combination of factor levels (4 factors, 20 levels each) is termed a level-combination, which can be regarded as a point in the experimental domain. We use the symbol “*n*” to denote the number of experimental runs, with each run producing a level-combination, i.e. a tetrapeptide sequence. A set of experimental designs can be represented as a matrix $U=x_{1},x_{2},\ldots ,x_{n}$, where $x_{j}$  ∈  χ and *n* equals to 1000 to 16 000 with an increment of 500.

**Figure 2:**
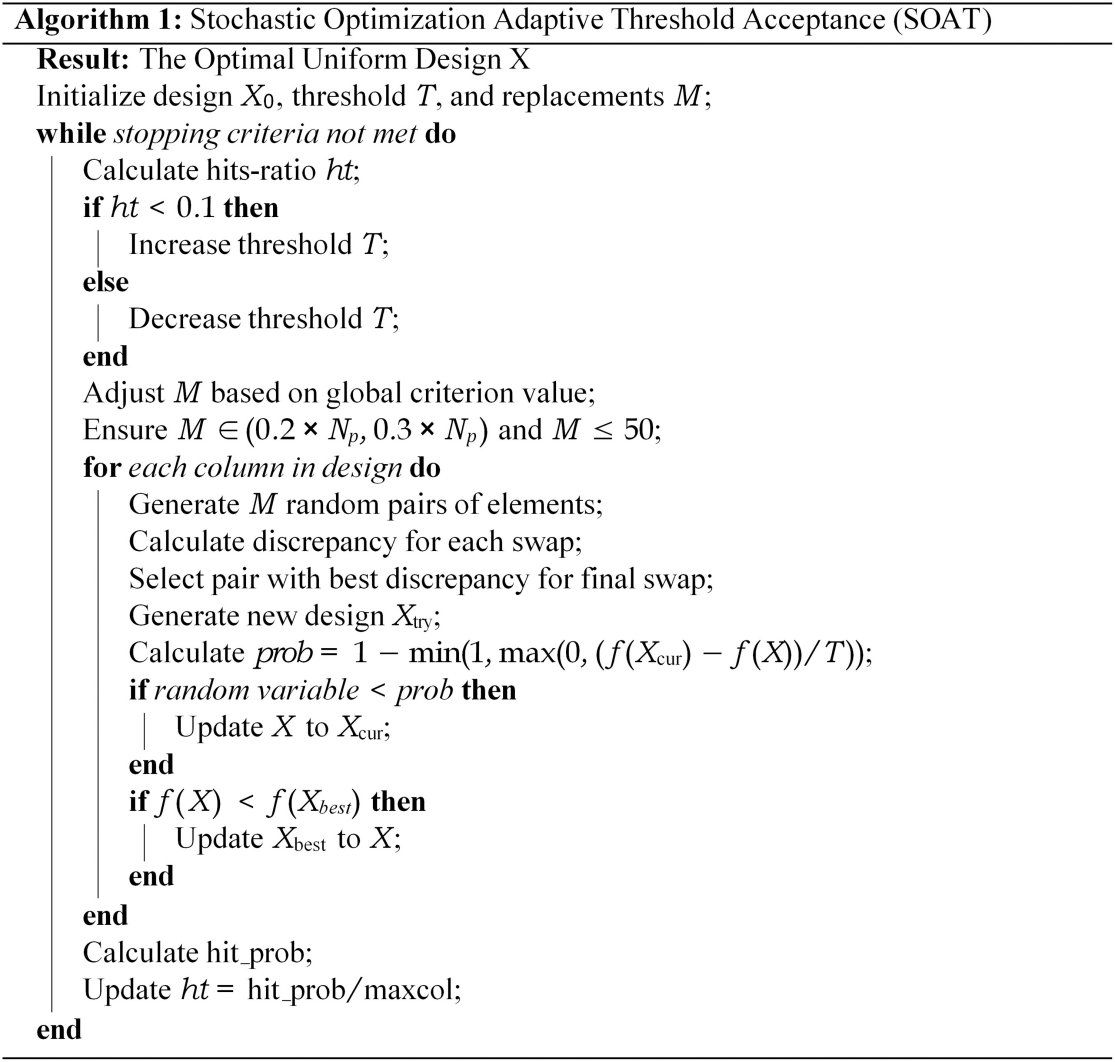
Algorithm of the stochastic optimization adaptive threshold acceptance (SOAT) technique.

Sampling was performed using the GenUD function from the R package UniDOE (Zhang *et al.* 2018), which utilizes the stochastic optimization adaptive threshold acceptance (SOAT) algorithm (Fig. 2). Unlike traditional fixed-threshold methods (Fang and Lin 2003), SOAT dynamically adjusts thresholds to escape local optima, efficiently generating designs with minimal deviation from uniformity.

The optimization objective was mixture L2-discrepancy (MD2), a metric proposed by Zhou *et al.* (2013) that integrates the advantages of centralized L2-discrepancy and wrap-around L2-discrepancy (Hickernell 1998). It also satisfies the eight uniformity evaluation criteria established by Fang *et al.* (2005), making it particularly suitable for amino acid sequence spaces, as it preserves the uniformity while accounting for the periodic boundary requirements. The MD2 formula is


(3) $$\begin{array}{c} MD2(P)={\left(\frac{19}{12}\right)}^{s}-\frac{2}{n}\sum_{i=1}^{n}\prod_{j=1}^{s}\left(\frac{5}{3}-\frac{1}{4}\left|x_{ij}-\frac{1}{2}\right|-\frac{1}{4}{\left|x_{ij}-\frac{1}{2}\right|}^{2}\right)\\ +\frac{1}{n^{2}}\sum_{i=1}^{n}\sum_{k=1}^{n}\prod_{j=1}^{s}(\frac{15}{8}-\frac{1}{4}\left|x_{ij}-\frac{1}{2}\right|-\frac{1}{4}\left|x_{kj}-\frac{1}{2}\right|\\ -\frac{3}{4}\left|x_{ij}-x_{kj}\right|+\frac{1}{2}{\left|x_{ij}-x_{kj}\right|}^{2}) \end{array}$$

where *n* is the number of experimental runs (1000 to 16 000 with increment of 500), *s* (=4) is the factor of the experiment.

**Figure 3:**
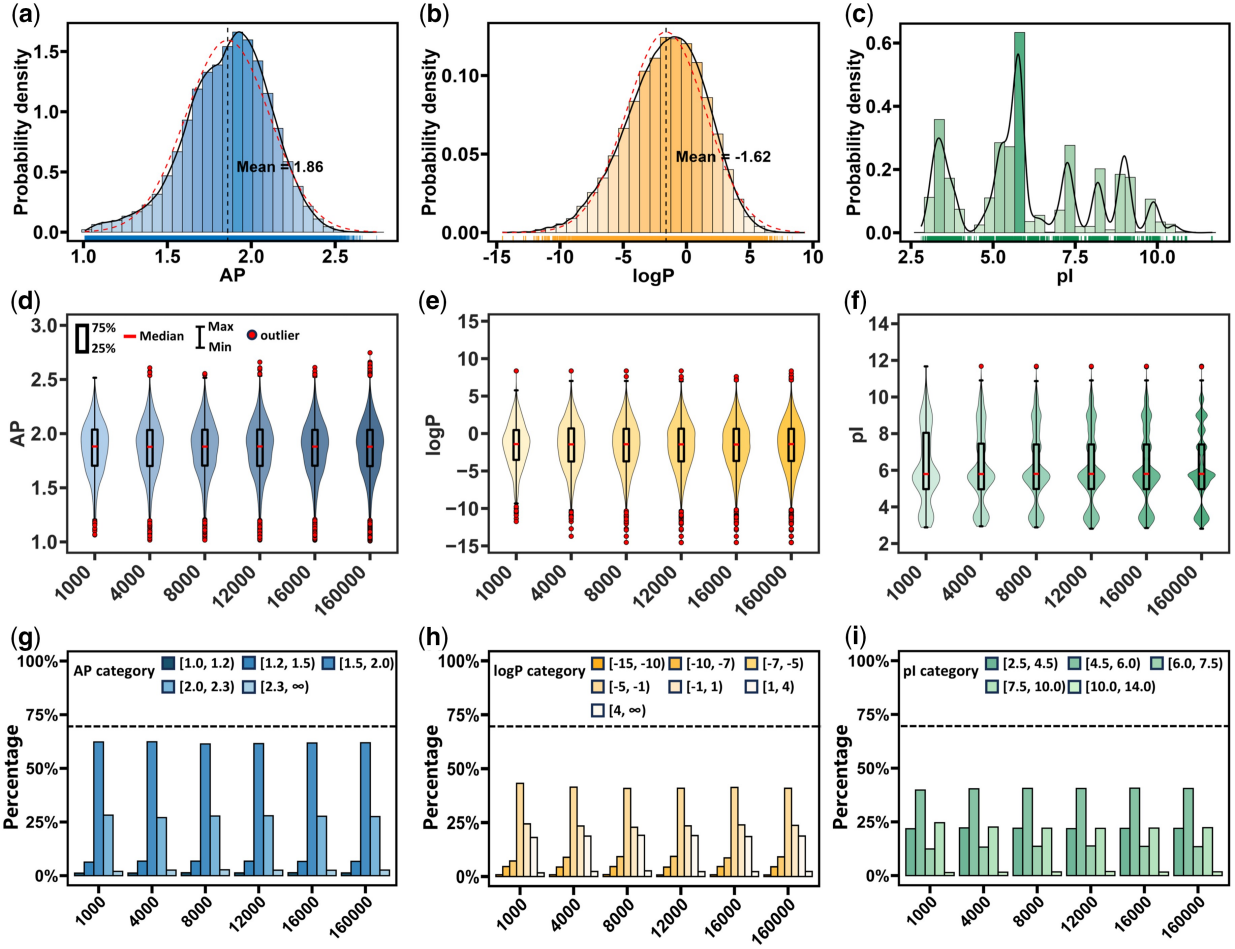
Comparison of distribution patterns of UD sampling data and overall data. (a–c) Histograms and density plots of AP, logP, and pI of 160 000 tetrapeptide sequences. The red dashed lines represent the fitted normal distribution, while the black solid lines indicate the actual density distribution of the data. The color gradient in the histograms reflects density, with darker colors corresponding to higher density. The carpet plots at the bottom of each chart display the distribution of individual data points. (d–f) Violin plots comparing the distribution of AP, logP, and pI properties between UD samples (sizes of 1000, 4000, 8000, 12 000, and 16 000) with the overall population data. (g–i) Proportions of sampled data relative to the overall dataset, after segmenting AP, logP, pI into defined intervals.

MD2 was minimized via SOAT (Fig. 2), which uses nested loops to adjust thresholds and swap design points, probabilistically accepting suboptimal designs to avoid local optima while preserving uniformity. This sampling technique maximizes the coverage of the design space with a minimal number of samples, ensuring each subset accurately represents the full sequence space and providing high-quality training data for subsequent AI modeling. The strict per-position, per-amino acid frequency uniformity achieved by UD represents a key distinction from conventional space-filling designs (e.g. Latin Hypercube, Sobol, and Halton sequences), which do not offer such a guarantee, as confirmed by our comparative analysis (Figs 1–3, available as supplementary data at *Bioinformatics* online).

### 2.4 Training and testing of AI models

This study employs two classical ML algorithms, i.e. RF and SVM, and one deep learning algorithm, i.e. transformer, to predict three properties of tetrapeptides, namely AP, logP, and pI. For RF and SVM, tetrapeptide sequences were converted to 80-dimensional vectors via one-hot encoding. Hyperparameter optimization involved random search over 50 combinations, evaluated via five-fold cross-validation; the optimal hyperparameter configurations were selected based on validation set RMSE. The hyperparameter search space for RF and SVM is shown in Table 1, available as supplementary data at *Bioinformatics* online.

Given the potential instability and convergence issues arising from variations in training dataset size (1000–16 000 sequences), the transformer model was optimized accordingly: a linear warmup learning rate strategy was implemented, starting at 0.001 and gradually increasing to 0.2 during the warm-up phase, combined with a stochastic gradient descent optimizer (Bottou *et al.* 2018) with a momentum of 0.9. These adjustments aim to accelerate convergence, reduce oscillations during training, and enhance the model’s adaptability to different data scales, thereby enabling robust property prediction for tetrapeptide sequences. The transformer architecture consisted of a 6-layer encoder with 8 attention heads per layer (each of dimension 64), a feed-forward dimension of 2048, and a token embedding dimension of 512. This was followed by a 5-layer multilayer perceptron (MLP) regressor with hidden dimensions of 512, 256, 64, and 32, culminating in a single output neuron. Batch normalization and dropout (rate = 0.3) were applied within the MLP to mitigate overfitting on our limited, variable-sized datasets.

Training and validation data were split at 80% and 20%, respectively (training dataset in Supplementary Data 2). To thoroughly assess the generalization and predictive stability, two distinct testing set selection strategies were adopted after hyperparameter optimization: (i) fixed testing sets consisting of 10 000 sequences randomly selected from the full tetrapeptide space excluding training data (Supplementary Data 3) and (ii) nonfixed testing sets comprising remaining sequences (144 000–159 000, depending on training size) from the complete space (Supplementary Data 4). A total of 279 models were trained (31 datasets × 3 properties × 3 algorithms) and evaluated using MAE, RMSE, MSE, and *R*^2^, with full metrics for training, validation, and testing sets provided in Supplementary Data 5–9.

### 2.5 Tree-based model

This work employs three gradient boosting decision tree (GBDT) models, i.e. XGBoost, LightGBM, and CatBoost to investigate the principal variables influencing the AP values of tetrapeptides. These models excel at handling nonlinear and large datasets, making them suitable for analyzing the full 160 000-sequence tetrapeptide space. We categorized the AP values into three classes based on a detailed understanding of peptide self-assembly behavior: Class 0 (AP ∈ [1, 1.5)), Class 1 (AP ∈ [1.5, 2)), and Class 2 (AP ∈ [2, 2.5]). Class 1 represents the optimal aggregation state, exhibiting the most promising characteristics for achieving self-assembly.

Feature extraction integrated 726 descriptors: physicochemical and structural characteristics from the R package “protr” (version 1.7-4) (Xiao *et al.* 2015); supplementary properties (net charge, hydrophobicity, Boman’s index, and others) from “peptides” (version 2.4.6) (Osorio *et al.* 2015); and experimentally determined logP values as well as AI-predicted pI values. These features are detailed in Supplementary Code 3 and Data 10.

A multimodel voting strategy is used to guarantee the selection of the most significant features. The average importance score for each feature is calculated using the built-in feature importance scoring functions of the XGBoost, LightGBM, and CatBoost models, in conjunction with five-fold cross-validation. A dynamic threshold (mean importance plus one standard deviation) is established to identify features deemed significant in each model. Ultimately, features that are recognized as significant by at least two models are selected, ensuring consistency and relevance across models. This strategy substantially reduces the feature set and eliminates redundancies.

**Figure 4:**
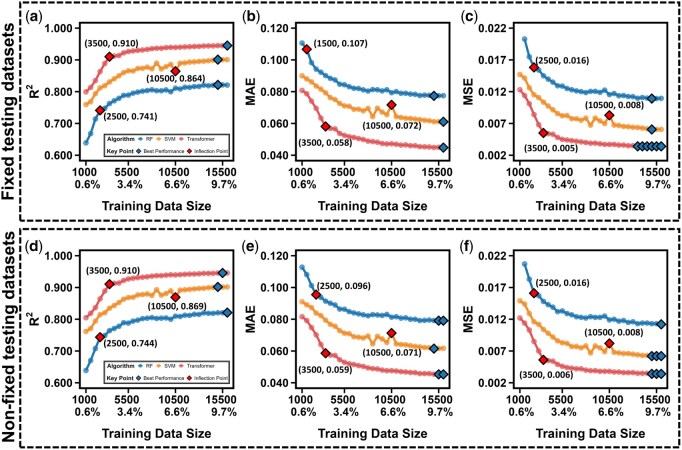
Learning curves of model performance. (a–c) Performance metrics (*R*^2^, MAE, MSE) of the RF, SVM, and transformer models in predicting AP using a fixed testing dataset, evaluated across varying training set size ranging from 0.625% (size 1000) to 10% (size 16 000) of the entire tetrapeptide sequence space (160 000 sequences). Blue diamonds mark the relative best performance points for each model, while red diamonds highlight inflection points where the performance trends change significantly. The inflection points are determined by the extreme value points of the second derivative, indicating significant curvature change in the learning curve. (d–f) Performance metrics (*R*^2^, MAE, MSE) of the RF, SVM, and transformer models in predicting AP using a nonfixed test set, evaluated across varying training set sizes.

Data imbalance was addressed via stratified sampling during preprocessing, ensuring balanced class distribution across the 80% training and 20% testing split of the 160 000 tetrapeptide sequences. For hyperparameter tuning, 50 combinations were evaluated using stratified five-fold cross-validation on the training set, with optimal settings selected based on negative log-loss to enhance robustness against imbalance (search space in Table 2, available as supplementary data at *Bioinformatics* online). Model performance was thoroughly assessed using six metrics (accuracy, precision, recall, F1 score, ROC AUC, PR AUC; Powers 2020), with the corresponding results provided in Supplementary Data 11. Visualizations (confusion matrices, ROC curves, and PR curves) are presented in Fig. 4, available as supplementary data at *Bioinformatics* online.

### 2.6 SHAP analysis

To quantitatively decode the mechanisms underlying tetrapeptide self-assembly, we applied Shapley Additive Explanations (SHAP) analysis (Lundberg and Lee 2017), a game-theoretic approach that quantifies how physicochemical and structural features influence AP. This analysis identifies critical features and dissects their roles in model predictions, including their overall impact, main effects, and interaction effects. In this study, SHAP analysis serves two primary purposes. First, it acts as a critical validation tool, confirming that our purely sequence-based AI models have learned to recognize fundamental physicochemical drivers of self-assembly without prior instruction, thereby enhancing model trustworthiness. Second, it provides novel, quantitative insights that refine our understanding beyond qualitative rules.

The SHAP framework proposes an additive decomposition of a model’s prediction f(x) for any instance, succinctly expressed as $f(x)=\varphi_{0}+\sum_{i}\varphi_{i}\sum_{i<j}\varphi_{ij}$, where $\varphi_{0}$ represents the baseline prediction (no features); $\varphi_{i}$ denotes main effect values, reflecting the average contribution of each feature *i* across various feature backgrounds; and $\varphi_{ij}$ represents interaction effect values, capturing the pure synergistic impact of features *i* and *j* (independent of their individual effects). This decomposition clarifies both individual and interactive feature impacts, translating complex ML predictions into interpretable scientific insights. These findings inform rational design strategies for peptide-based nanomaterials and deepen understanding of molecular self-assembly rules.

## 3 Results and discussion

### 3.1 Distribution of UD-sampled properties

**Figure 5:**
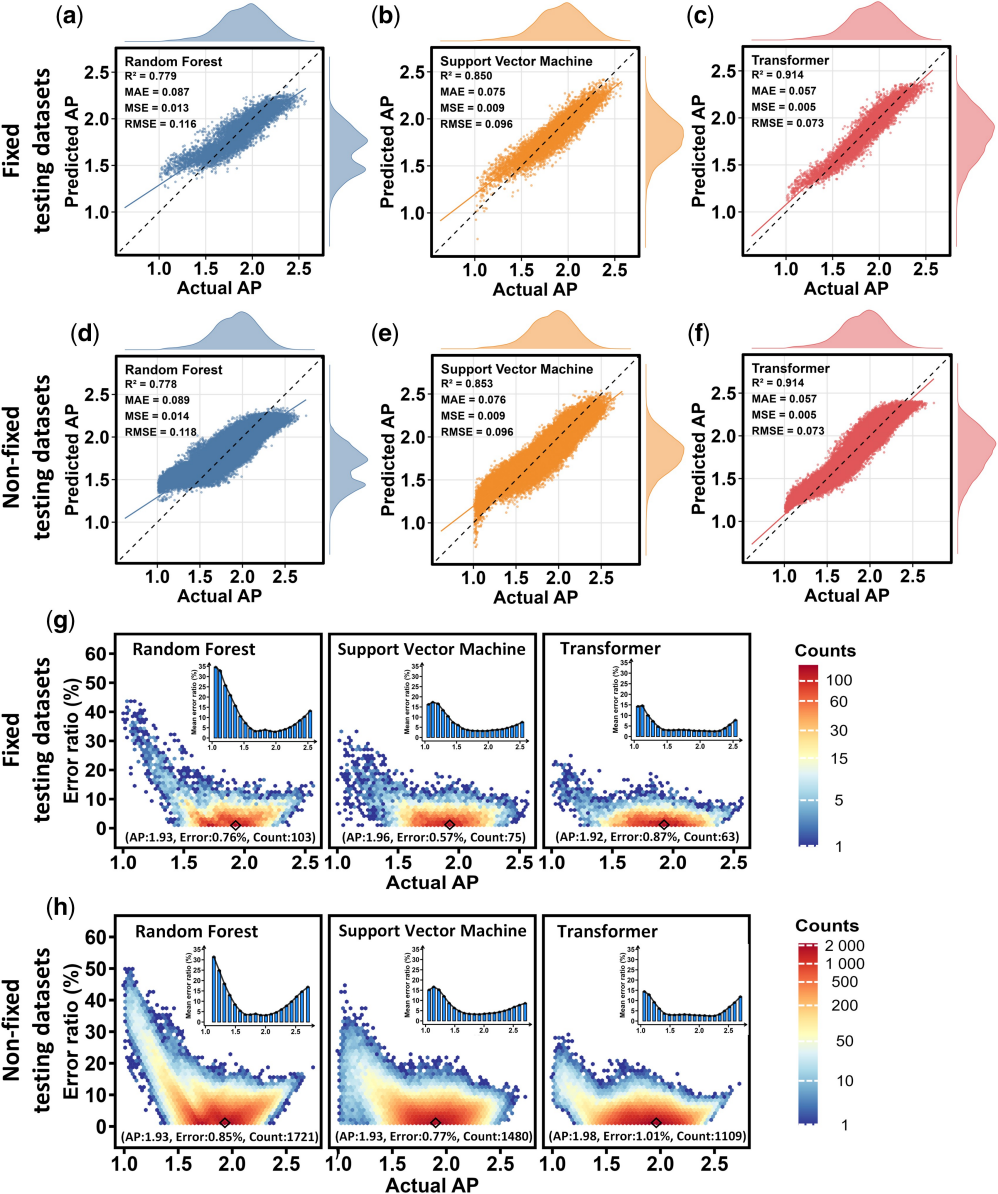
Cross-model prediction error analysis of AP. (a–f) The relationship between the actual and predicted AP values of the RF, SVM, and transformer models, evaluated using fixed and nonfixed testing dataset, with a training set of 4500. Each plot includes a linear regression line (solid line) and an ideal prediction line (dashed line). Density plots are attached to the edges of the plots to illustrate the distribution of predicted and actual values. (g–h) The relationship between actual AP values and corresponding prediction errors, visualized using hexagonal density plots for fixed and nonfixed testing dataset with the same training data. The prediction error is calculated as a percentage of the absolute difference between the actual and predicted AP value versus the actual AP values. The color gradient from blue to red indicates increasing point density, with the highest density points marked by black diamonds and corresponding coordinates labeled below. Histograms show the average prediction error rates for the RF, SVM, and transformer models across the AP value range.

To evaluate the effectiveness of the UD sampling method, we analyzed the distribution patterns of AP, logP, and pI across the sampled sequences with varying sizes (1000, 4000, 8000, 12 000, 16 000) and compared them with those of the entire tetrapeptide sequence space (Fig. 3). The AP values of the 160 000 tetrapeptides conform to a normal distribution with a mean of 1.86 (Fig. 3a), while logP values exhibit an approximately normal distribution centered at −1.62 (Fig. 3b). In contrast, the pI values display a more complex, multimodal distribution (Fig. 3c). The sampled distributions of AP and logP are highly consistent with the overall distributions (Fig. 3d and e). Although the distribution of pI values exhibits greater complexity, the sampled distribution increasingly aligns with the overall distribution as the sample size increases (Fig. 3f). Further analyses show that the proportions of AP, logP, and pI intervals in the sampled data from 31 experimental design sets closely approximate those in the total dataset of 160 000 sequences (Fig. 3g–i; Fig. 5a–c, available as supplementary data at *Bioinformatics* online).

**Figure 6:**
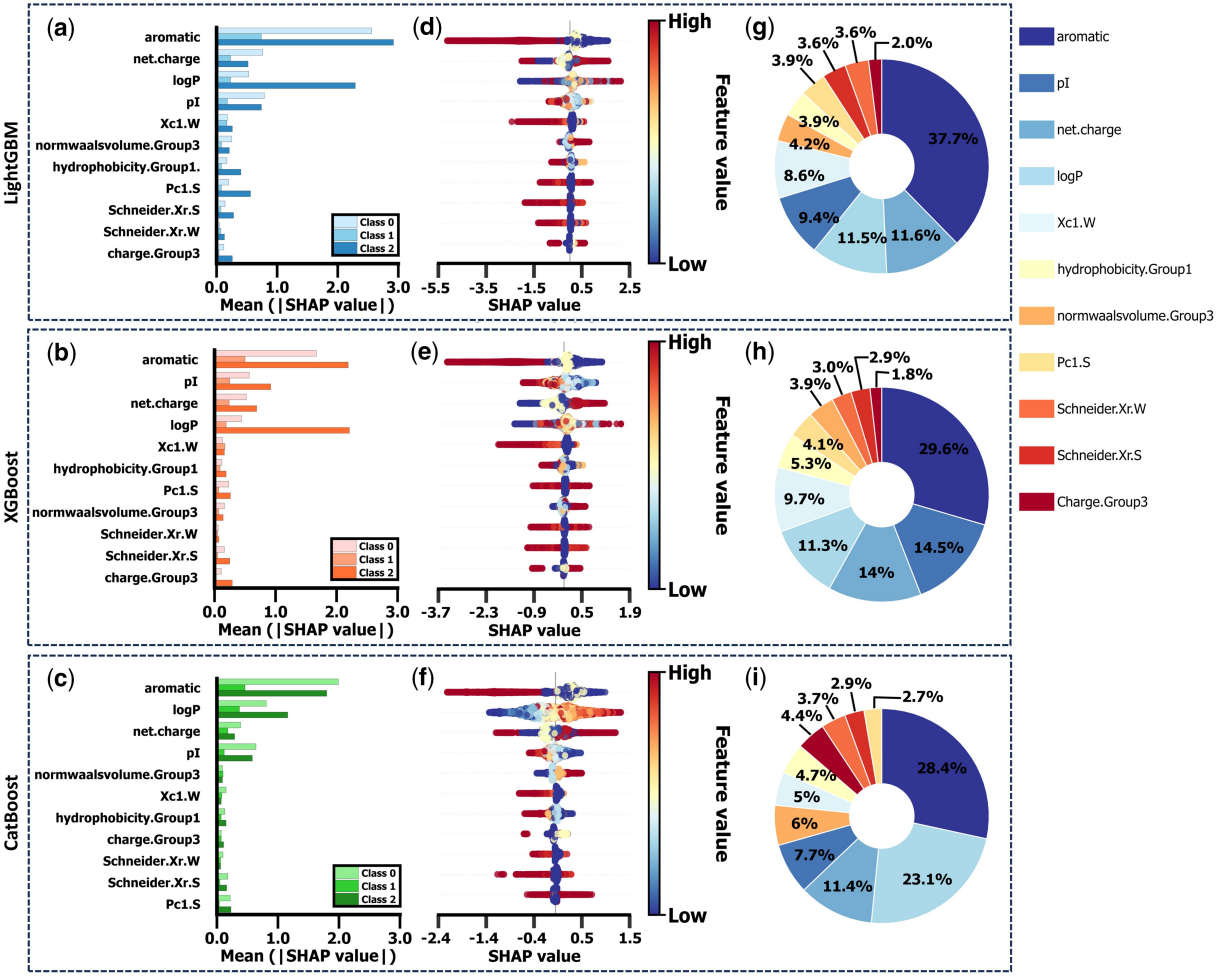
Comprehensive SHAP analysis for tetrapeptides’ AP values in classification models. (a–c) SHAP analysis using LightGBM, XGBoost, and CatBoost models from top to bottom. The summary plot displays the mean absolute SHAP values for 11 features, ranked in descending order of importance. Values represent the average impact of features on predictions using the testing set, which includes 32 000 tetrapeptide sequences (20% of the total 160 000 sequences), covering Class 0, Class 1, and Class 2. (d–f) Beeswarm plots illustrating the global distribution of features for Class 1 classification across the three models. Each point represents a sample’s SHAP value, with colors indicating feature values (red for high, blue for low). The horizontal position denotes the SHAP value magnitude (negative values indicate negative impact and positive values indicate positive impact), while vertical stacking indicates a higher density of SHAP values in that region. The order of features from top to bottom matches the order in panels (a–c). (g–i) Pie plots showing the percentage contribution of each feature to the predictions for Class 1 classification. The percentages are calculated based on each feature’s mean absolute SHAP value within each model.

These comparisons demonstrate that the sampled data from various sizes can adequately represent the characteristics of the entire dataset. This finding underscores the ability of the UD method to mitigate sampling bias and guarantee comprehensive coverage of sequence diversity by maintaining a uniform frequency of 0.05 for each amino acid at each position (Figs 5d and 6, available as supplementary data at *Bioinformatics* online). This approach, in turn, lays a robust foundation for AI models to achieve superior accuracy and generalization in predicting tetrapeptide properties and beyond.

It is noted that Theorem 1 establishes the optimality of the UD with an assumption of totally unknown *f*, which, in practice, this could be too strong. In this scenario, UD may no longer be the optimal design. However, one could always start with a UD and then adjust the design depending on the analysis results, which will be a subsequent research topic.

### 3.2 Performance evaluations of AI models

The transformer model consistently exhibits exceptional performance across all attributes (AP, logP, pI), as evaluated by metrics of MAE, MSE, and *R*^2^ (Fig. 4). Results of AP prediction for both fixed (Fig. 4a–c) and nonfixed (Fig. 4d–f) testing datasets demonstrate that the transformer model is more efficient at utilizing smaller (also larger) training datasets effectively compared to the RF and SVM models. Notably, the transformer model achieves significant performance (*R*^2^=0.91) gains with training datasets of approximately 3500–4500 data points (Tables 3 and 4, available as supplementary data at *Bioinformatics* online). This high performance is robust and reproducible, as confirmed by multiseed runs detailed in Table 5, available as supplementary data at *Bioinformatics* online. Beyond this range, performance gains plateau, indicating that additional data contribute minimally to further improvement. Thus, the performance convergence threshold for the transformer model in predicting tetrapeptide properties can be set at around 3500–4500 data points, corresponding to only 2.19%-2.81% of the entire tetrapeptide sequence space.

Conversely, the RF model reaches its performance inflection point with a smaller training dataset (about 2000–2500 data points) but exhibits relatively inferior performance compared to the transformer model. Meanwhile, the SVM model slightly outperforms the transformer in predicting the logP property (Fig. S7, available as supplementary data at *Bioinformatics* online) but underperforms in predicting the AP and pI (Fig. 4; Fig. 8, available as supplementary data at *Bioinformatics* online, respectively). These trends are consistent across both fixed and nonfixed testing sets.

The ability of AI models, particularly the transformer, to achieve robust performance with such a small fraction of the sequence space is rooted in the properties of the UD sampling strategy. UD is theoretically designed to maximize the diversity and representativeness of the selected subset. We quantitatively validated this by analyzing the Hamming distance between all training and testing sequences (Fig. 9 and Table 6, available as supplementary data at *Bioinformatics* online). The results show an extreme degree of separation: on average, sequences differ in 3.8 out of 4 positions, and critically, 100% of all training–testing pairs differ by at least two residues. This high degree of dissimilarity ensures that the model cannot rely on memorizing local sequence patterns or trivial single-residue variants; instead, it is compelled to learn the broader, underlying physicochemical principles that govern sequence–property relationships. This synergy between the UD sampling strategy and the advanced transformer model architecture, coupled with the accurate identification of performance inflection points, enables transformer to demonstrate superior learning efficiency and predictive capability on medium-sized data sets.

RF’s inferior performance compared to other methods could be partly attributed to the incomplete information of the problem, where properties of amino acids are not included in analysis datasets. That results in an underrepresentation of the actual problem. As a result, we intend to add properties of amino acids to the sequence of the peptides in our future research and thus the input of *f* will change from a matrix to a tensor (3D matrix) and those methods will be upgraded to their counter-parts in tensor analysis. It is expected that the performance gaps between RF and other methods will be diminished with the addition of extra variables and the required sample size will be lowered as well.

### 3.3 Error analysis of AI model

To elucidate the complex interplay among diverse error sources, sampling methods, tetrapeptide properties, and model architecture, we conducted a comprehensive error analysis (Fig. 5) on the RF, SVM, and transformer model trained with 4500 sequences, representing the convergence threshold for transformer model performance. The model performance with different size, property, and model type can be found in Figs 10–18, available as supplementary data at *Bioinformatics* online.

Regarding the AP, all models exhibit elevated error rates in the lower AP range (1 < AP<1.5) as well as the high AP range (AP>2.3, Fig. 5a–f). This phenomenon may be attributed to the inherent scarcity of tetrapeptides at extreme AP values, which limits model learning in these intervals. In the intermediate range (1.5 < AP<2), the error ratio stabilizes at around 2.5% (Fig. 5g and h), while the SVM and RF models show marginally higher error ratios of 3%–5% (Fig. 5g and h). This stable error ratio suggests that the models can effectively capture the features of the tetrapeptide sequences in this region. This capability may be related to the relatively higher density of sampled properties in this intermediate range, where abundant data improve model performance and reduce both bias and variance in the errors.

In the prediction of logP, the model exhibits an unusually high error rate in the logP interval from −1 to 1 (Fig. 19, available as supplementary data at *Bioinformatics* online). This phenomenon is related to an inherent limitation of the error calculation method: when the logP value approaches zero, it acts as a denominator, thereby inflating the error proportion at extreme logP values (−15 < AP −10 and AP>4, representing only 2.95% of the samples). Significant differences in model performance emerge in these sparse regimes, i.e. the RF model has an average error rate of 30%–40%, while the transformer has an average error rate of 10%–20%, and the SVM model approaches an error rate of zero (Fig. 19, available as supplementary data at *Bioinformatics* online). This discrepancy may reflect differences in how each model handles sparse data regions, particularly when predicting extreme logP values, where SVM demonstrates unexpectedly high accuracy.

The pI predictions exhibit stable error ranges across interval of 5–9 (Fig. 20, available as supplementary data at *Bioinformatics* online), with mean error ratios generally below 10% for all models. However, in the low pI range (from 2.5 to 4.5), the RF and SVM models show higher error rates. The transformer model also displays a marked increase in error within the range of 4–4.5 (Fig. 20, available as supplementary data at *Bioinformatics* online). These inconsistencies can be attributed to the multimodal complexity of the pI distribution and the varying abilities of the models to capture this complexity.

In summary, the transformer model, leveraging its self-attention mechanism, adeptly captures long-term dependencies of intricate features, achieving minimal prediction error within data-dense intervals. Despite elevated errors in extreme intervals, the transformer consistently outperforms SVM and RF models. While SVM excels at predicting logP values, it underperforms in predicting AP and pI. This disparity likely stems from the complex interplay between tetrapeptide properties and model features, where logP values exhibit more linear correlations with tetrapeptide sequences following one-hot encoding, favoring SVM, whereas AP and pI involve more complex nonlinear relationships that require the enhanced feature extraction abilities of the transformer. By integrating UD sampling, multimodel comparisons, and detailed error analyses, this study reveals the intricate challenges in predicting tetrapeptide properties, highlighting the importance of selecting appropriate algorithms based on specific properties and data characteristics. These findings provide valuable insights for future peptide–property prediction studies and guide the optimization of sampling strategies and model architectures to enhance performance.

### 3.4 Feature impact on aggregation

In this study, three integrated learning tree models, i.e. XGBoost, LightGBM, and CatBoost, combined with SHAP analyses are employed to systematically evaluate the key features influencing the aggregation of tetrapeptides. The robustness of these SHAP-based interpretations against potential confounding factors like feature correlation was comprehensively validated through multimodel consensus, bootstrap stability analysis (Table 7 and Fig. 21, available as supplementary data at *Bioinformatics* online), permutation importance correlation (Figs 22 and 23, available as supplementary data at *Bioinformatics* online), and partial dependence plots and individual conditional expectation (ICE) plots (Supplementary Section 4.1 and Fig. 24, available as supplementary data at *Bioinformatics* online). All three models exhibit superior performance in multiclass classification (Table 8 and Fig. 4, available as supplementary data at *Bioinformatics* online), with complete feature importance data and a summary visualization provided in Supplementary Data 12 and Fig. 25, available as supplementary data at *Bioinformatics* online, respectively. We pay special attention to the features affecting the ideal AP range (Class 1: 1.5 < AP<2) for achieving self-assembly, with analyses focusing on their total effect (SHAP value), main effect (SHAP main effect, SHAP${\;}_{\text{main}}$) and interactive effect (SHAP interactive effect, SHAP${\;}_{\text{inter}}$). The results of the SHAP analyses for other classes (Class 0: 1 < AP<1.5 and Class 2: AP>2) are presented in Figs 26–44, available as supplementary data at *Bioinformatics* online.

#### 3.4.1 *SHAP total effect*

The assessment of model performance reveals that all three models exhibit high accuracy, demonstrating their robust discriminative capabilities in multicategory classification tasks. Through a thorough analysis of the SHAP dependence results from the three models, we identified four principal features via intersection analysis: aromaticity, pI, net charge, and logP. Although the specific ranking of these features varies across the models (Fig. 6, Class 1), there is unanimous agreement that aromaticity exerts the most significant impact on the AP of tetrapeptides, corroborating prior findings by Wang *et al.* (2024).

**Figure 7:**
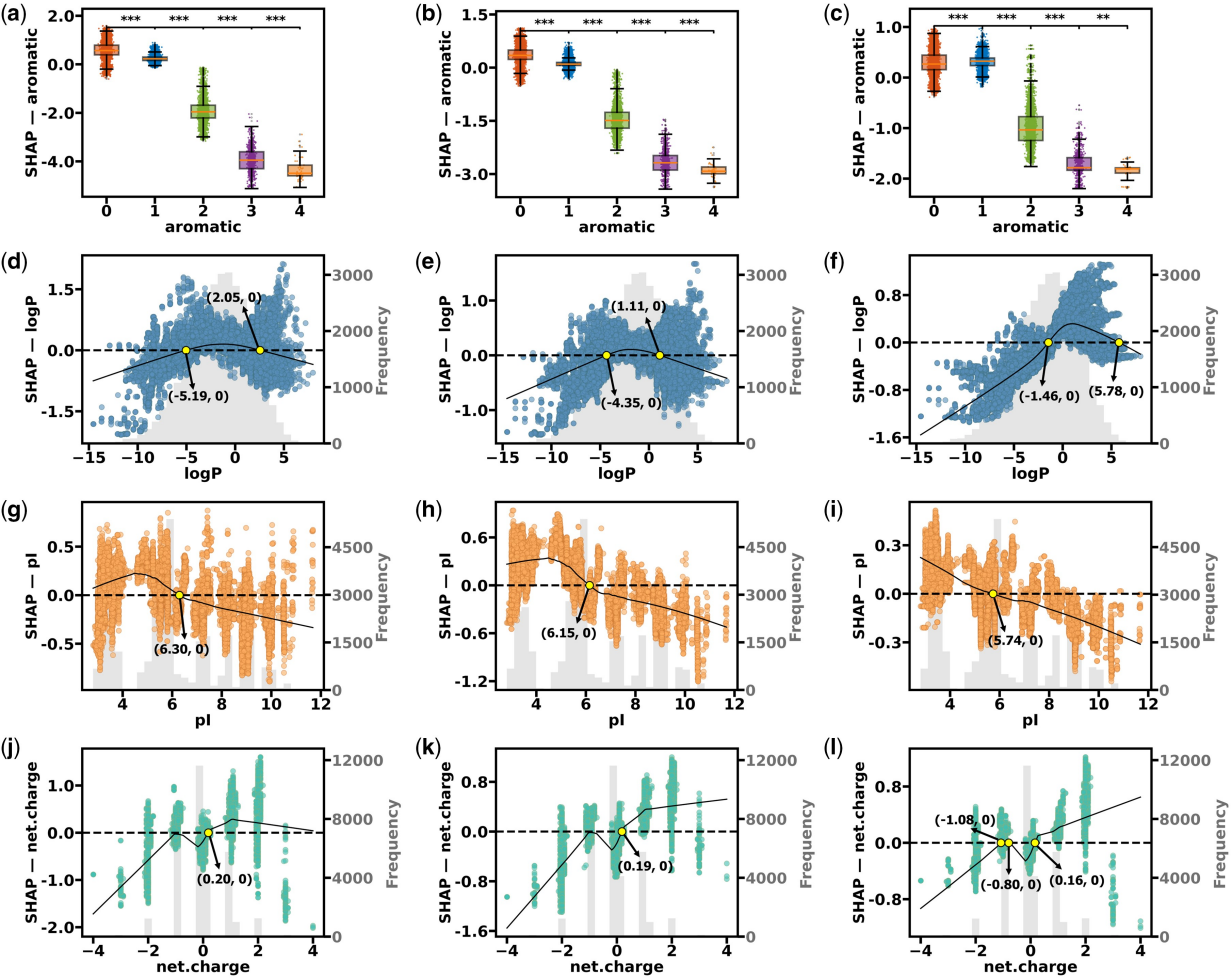
SHAP dependence plots for top features in Class 1 of aggregation. (a–c) Impact of aromatic residue count on Class 1 SHAP values for LightGBM, XGBoost, and CatBoost models. Box plots show SHAP value distributions for different aromatic residue counts (0, 1, 2, 3, and 4). Boxes represent interquartile ranges, middle lines indicate medians, and whiskers extend to 1.5 times the interquartile range. Asterisks (*) above box plots indicate statistical significance levels of SHAP value differences between adjacent groups. Significance levels are based on Mann–Whitney *U* tests with Bonferroni correction: ***P < .00025 (0.001/4), **P < .0025 (0.01/4), *P < .0125 (0.05/4), NS: *P* ≥ .0125 (not significant). (d–f) SHAP dependence plots for the logP feature in Class 1 for LightGBM, XGBoost, and CatBoost models. Gray histograms (referring to the right axes) reflect the frequency distribution of logP values in the testing dataset. Black LOWESS (locally weighted scatterplot smoothing) fitting curves show the overall trend between logP and SHAP values. Black dashed lines indicate the SHAP value of 0 baselines, while yellow markers highlight critical values where the impact of logP on predictions shifts from positive to negative (or vice versa). (g–i) SHAP dependence plots for the pI feature in Class 1 for LightGBM, XGBoost, and CatBoost models. These plots include scatter points, gray histograms, and black LOWESS curves, similar to panels (d–f). Yellow points mark critical values where the impact of pI shifts. (j–l) SHAP dependence plots for the net charge feature in Class 1 for LightGBM, XGBoost, and CatBoost models. These plots include scatter points, gray histograms, black LOWESS curves, and intersection annotations to comprehensively show how net charge affects model predictions.

*Aromaticity—total SHAP effect.* Total SHAP values (incorporating both main and interactive effects; Fig. 7) revealed a striking threshold effect of aromatic residues (F, H, W, Y) on Class 1 aggregation (optimal for self-assembly). Specifically, 0–1 aromatic residue contributed positively to ideal aggregate formation, whereas ≥2 residues hindered this process (Figs 6d–f and 7a–c). Statistical validation via Mann–Whitney *U* tests confirmed this transition: increasing aromatic residues from 1 to 2 caused a significant drop in SHAP values (XGBoost: 156.64%, LightGBM: 220%, CatBoost: 128.14%), with all *P*<.001. Significance persisted after Bonferroni correction (adjusted *P* < .00025), providing robust support for the threshold hypothesis.

*logP—total SHAP effect*. The effect of logP on the ideal aggregation state exhibits a distinct “window effect.” Specifically, moderate logP values facilitate Class 1 outcomes, while both excessively high or low logP values are detrimental to the optimal aggregation of tetrapeptides (Figs 6 and 7d–f). Cross-model comparison reveals consistent favorable ranges: LightGBM and XGBoost converge on logP = −5 to 2 kcal/mol, whereas CatBoost suggests a broader interval of −1.5 to 6 kcal/mol (Fig. 7d and e). The moderate hydrophilicity enhances intermolecular attraction between peptides in aqueous environment, prompting tetrapeptide molecules to form a hydrophobic core that promotes self-assembly. However, excessively low logP values may lead to solubility issues (i.e. precipitation), while extremely high logP values may result in solutions instead of self-assemblies.

*pI—total SHAP effect.* The relationship between the pI and AP of tetrapeptides highlights the delicate influence of pI (i.e. charge) to peptide aggregation processes. For ideal self-assembly (i.e. Class 1), lower pI values exert a beneficial effect on aggregate formation, while pI approaching 6 has minimal influence and high pI hinders this process (Figs 6 and 7g–i).

Examination of the SHAP dependence plots for pI in the LightGBM, XGBoost, and CatBoost models reveals that the inflection points are approximately situated at pI values of 6.3, 6.15, and 5.74, respectively. Below these thresholds, pI positively influences aggregation (with simulation at pH = 7), with charge interactions accumulating to a peak around the inflection point. Beyond this range, increasing pI diminishes its beneficial effect and eventually impedes Class 1 outcomes (Fig. 7g–i).

This behavior is likely linked to the dynamic alteration in the charge shielding effect: at low pI conditions (below 6.15), negative charges (induced by external pH = 7) promote the ordering and formation of stable aggregated structures, probably mediated by the directional hydrogen bonding between water molecules and peptides. Conversely, with elevated pI values (above 6.3), the excessive positive charges on the peptide chain results in solvation, thereby impeding the formation of stable aggregates.

*Net charge—total SHAP effect.* Regarding net charge, high net charge values positively contribute to Class 1 formation, while lower net charge values adversely affect it (Figs 6 and 7j–l). The SHAP dependence plot of net charge corroborates this pattern; the LOWESS smoothing curve indicates that as net charge value increases, its average SHAP effect on Class 1 also rises. At a net charge value of approximately 0.20, all three models exhibit an inflection point where the SHAP value transitions from negative to positive, signifying that the positive contribution of net charge to Class 1. However, when the net charge value exceeds 3, almost all scatter points are clustered on the side with negative SHAP values, although the LOWESS curve remains positive (Fig. 7j–l). This discrepancy may arise from sparse data points in this region, where local smoothing and boundary effects elevate the LOWESS curve above the actual SHAP values. The negative SHAP values at very high net charge values (>3) suggest that local instability can arise under these extremely high-charge conditions.

#### 3.4.2 *SHAP main effect*

Detailed analysis of feature independent contributions (SHAP${\;}_{\text{main}}$), including their patterns and underlying mechanisms, is provided in the Supplementary Section 4.2 and Fig. 44, available as supplementary data at *Bioinformatics* online (Class 1 only). These results complement the total effects by isolating individual feature impacts.

#### 3.4.3 *SHAP interactive effect*

Here, we focus on interactive effects between key features, which illuminate their combined influence on Class 1 aggregation.

**Figure 8:**
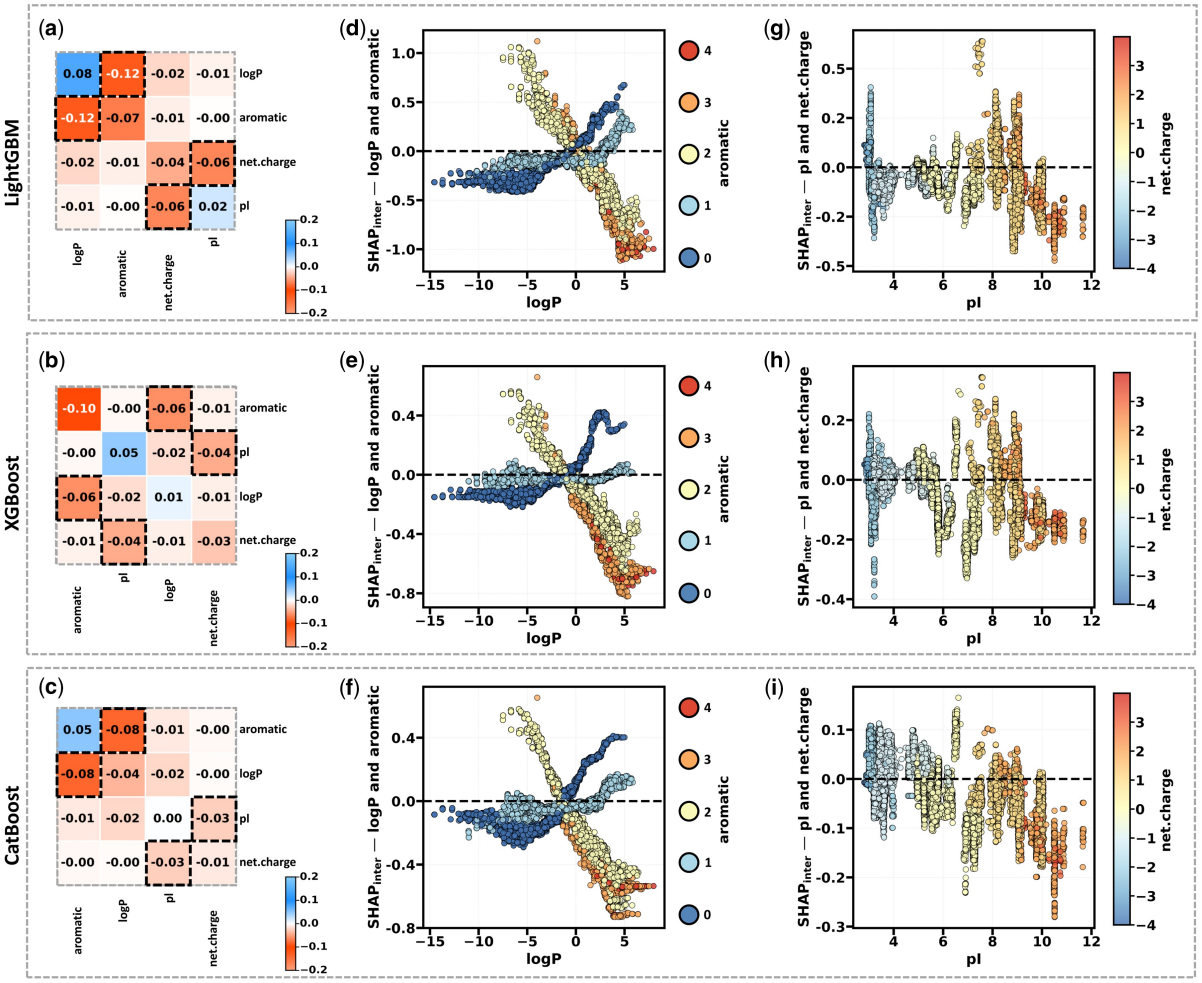
Interactive effect of SHAP plots for top features in Class 1 of AP. (a–c) Heatmaps of SHAP interaction values for top features in Class 1 for LightGBM, XGBoost, and CatBoost models (from top to bottom). Off-diagonal elements quantify the magnitude and direction of pairwise feature interactions, while diagonal elements represent the strength and direction of individual feature main effects (positive in blue, negative in orange). These heatmaps depict interactions among the top 4 features, while the complete interaction heatmap of all 11 features is provided in Fig. 20, available as supplementary data at *Bioinformatics* online. (d–f) SHAP interaction effect dependence plots for logP and aromatic features. The color gradient illustrates how the interaction between logP and aromatic features affects model predictions, with vertical distribution reflecting the degree and direction of their influence. (g–i) SHAP interaction effect dependence plots for pI and net charge features. Color gradients illustrate shifts in interaction impacts, while the vertical distribution of points highlights the influence degree and direction of the interactions on SHAP values.

*Aromatic—logP.* To systematically quantify the impact of interaction effects among key features on the formation of desirable aggregation patterns in tetrapeptide sequences, we employed two complementary methods of SHAP interaction analysis: feature interaction heatmaps based on SHAP values and dependence plots of SHAP interaction effects. The heatmaps indicate that the most pronounced interaction effect is between aromatic residues and logP (interaction values of −0.12, −0.10, and −0.08 in the LightGBM, XGBoost, and CatBoost models, respectively), followed by the interaction between net charge and pI (interaction intensities of −0.05, −0.04, and −0.03), as illustrated in Fig. 8a–c.

The dependence plots for the aromatic–logP interaction effect from the three models demonstrate that when logP is below −1 kcal/mol, an increase in aromatic residues in tetrapeptides leads to a higher SHAP interaction effect. Specifically, the effect ranges from −0.5 to 0 for sequences with no aromatic residues, −0.2 to 0 for those with one aromatic residue, and 0–0.5 for those with two or more aromatic residues. This interaction effect diminishes as logP approaches −1, becoming negligible around −1 regardless of the number of aromatic residues (Fig. 8d–f).

Conversely, when logP exceeds −1, the interaction effect is inverted. For tetrapeptides without aromatic residues, the interaction effect rises significantly with increasing logP. In contrast, the interaction effect increases more gradually for those with one aromatic residue. For tetrapeptides with two or more aromatic residues, the interaction effect is negative and decreases significantly with higher logP. This indicates that at low logP, more aromatic residues enhance aggregation, whereas at high logP, fewer aromatic residues are favorable for aggregate formation, and an excess may destabilize the aggregate structure.

*Net charge—pI.* The dependence plot of the net charge–pI interaction effect reveals a distinct color gradient trend, indicating significant variation in net charge values across different pI intervals (Fig. 8g–i). Specifically, increasing pI values correlate with a progressive transition in net charge from negative to positive.

Within the pI range below 6, the system’s charge distribution is predominantly negative (under external pH = 7), resulting in negative interaction effects. This suggests that in acidic environments, negative charges impede the development of Class 1 aggregates. As the pI value increases from 6 to 8, the charge state progressively approaches neutrality, leading to an equilibrium in charge distribution and a gradual shift toward positive net charge. During this transition, the interaction effect between net charge and pI exhibits no substantial positive or negative impact.

In the elevated pI range above 8, the solution becomes increasingly alkaline, resulting in less protonation of acidic amino acid side chains and a transition toward positive charge. The plot manifests as orange and red hues; however, the interaction impact remains negative. This indicates that the interplay between high pI and high net charge may result in an uneven charge distribution, thereby influencing the stability of the aggregation morphology.

*Interactions among other features.* Our analysis indicates that, alongside primary features, secondary variables subtly influence the aggregation tendency of tetrapeptides. Variables such as Pc1.S (amphiphilic pseudoamino acid composition for serine) and normwaalsvolume. Group 3 (normalized van der Waals volume Group 3), though individually minor in impact, offer supplementary dimensions for refining aggregation behavior. While the individual effects of these traits are small, their combined influence may be pivotal in accurately regulating the aggregation process, particularly when optimizing self-assembly behavior to achieve specific functions. These findings underscore the necessity of evaluating a comprehensive array of structural and physicochemical features when creating and optimizing peptide-based materials to achieve precise control over aggregation behavior.

In summary, we have established a comprehensive framework for studying the complex feature interaction network influencing the self-assembly behavior of tetrapeptides by combining multimodel ensemble methods with SHAP explanations. This methodology enhances the understanding of existing data and guides the future design of specialized self-assembling peptide materials with desirable functions.

## 4 Conclusion

This study investigates various physicochemical properties of the tetrapeptide sequence space using UD sampling approach, generating a high-quality, low-bias dataset for AI model training. The sampling technique maintains an equal frequency (1/20=5%) for each amino acid at every position, minimizing bias in amino acid type and position. Statistical analyses confirm that UD sampling produces representative subsets highly consistent with the theoretical sequence space in terms of AP, logP, and pI distributions.

In model performance evaluations, combining UD sampling with the transformer architecture demonstrates significant advantages and broad applicability. The technique achieves a performance inflection point at reduced training set sizes (3500–4500 sequences, 2.2%–2.8%) for predicting AP, logP, and pI, highlighting the framework’s efficiency in predicting peptide properties with limited data. This reduces data acquisition and computational resource requirements while maintaining high accuracy. This study indicates that the sampling fraction sf=n/N<3% should be the practical threshold. Such a synergy between sampling and analysis grants us a path to reach the theoretical bound.

Importantly, the proposed framework is designed with inherent scalability for longer peptide chains (e.g. 8–20 residues), which are critical for biomedical applications. Mathematically, the UD algorithm minimizes discrepancy independent of dimensionality, ensuring uniform amino acid coverage even as sequence length increases (as demonstrated in Fig. 6, available as supplementary data at *Bioinformatics* online). However, as sequence length grows, the computational burden of high-throughput molecular simulations remains a constraint, even for optimized sample sets. Consequently, future advancements must prioritize maximizing data efficiency to extract robust predictive power from limited, high-fidelity datasets.

Detailed error analysis reveals challenges in predicting extreme values and highlights the need for enhanced sampling density or targeted tuning techniques in these regions. These findings emphasize the importance of considering data attributes, problem complexity, computational efficiency, and model capability when selecting and optimizing predictive models.

Additionally, SHAP analysis confirms the model’s ability to capture essential biophysics from data and, more importantly, translates this understanding into novel quantitative design rules. These include a precise aromaticity threshold for optimal self-assembly, a defined hydrophobicity window, and nonadditive feature interactions, providing actionable guidance for the rational design of self-assembling peptides.

In summary, this work presents a comprehensive framework for examining and predicting tetrapeptide characteristics. It enhances understanding of the molecular principles governing self-assembly and offers quantitative insights for designing peptides with customized aggregation properties. The demonstrated synergy between UD and transformer establishes a sample-efficient paradigm for peptide property prediction. These findings advance the discovery of novel biomolecules and address challenges in biomedicine and materials science. Building on this paradigm, future work could explore integrating distilled knowledge from large-scale protein models into the UD-informed framework, potentially further reducing the sample size required to predict properties of longer peptides and extending the data-efficiency advantage showcased here.

## Supplementary Material

btag036_Supplementary_Data

## Data Availability

Supporting information including the supplementary data, code, results are available at the Github repository (https://github.com/JiaqiBenWang/UD-AI-Peptide), from Zenodo (https://doi.org/10.5281/zenodo.17984124), or from the author. The supporting information and the supplementary file of “List of Abbreviations and Acronyms” are available at *Bioinformatics* online.
