# Supplementary material for: Uniform design-embedded predictions of (tetra-)peptide physicochemical properties
Source: Bioinformatics. 2026 Jan 19;42(3):btag036. doi: 10.1093/bioinformatics/btag036 (PMC13032896; doi:10.1093/bioinformatics/btag036)
Supplement: btag036_Supplementary_Data [file btag036_supplementary_data.zip › List of Abbreviations and Acronyms.pdf]

List of Abbreviations and Acronyms

Uniform Design-Embedded Predictions of (Tetra-)Peptide Physicochemical Properties

Zhihui Zhu[2], Huapeng Liu[3], Xuechen Li[1], Haojin Zhou[3]*, Jiaqi Wang[1]*

*: Corresponding authors:

Haojin.Zhou@xjtlu.edu.cn; benwang@hku.hk

1. Department of Chemistry, State Key Laboratory of Synthetic Chemistry, The University of Hong Kong, Pokfulam 999077, Hong Kong SAR, China.
2. ZJU-Hangzhou Global Scientific and Technological Innovation Center, Zhejiang University, Hangzhou 311215, Zhejiang, China.
3. Wisdom Lake Academy of Pharmacy, Xi'an Jiangtong-Liverpool University, Suzhou 215123, Jiangsu, China.

This document provides a comprehensive list of abbreviations and acronyms used in the main text and supplementary materials of the manuscript.

Table 1. List of abbreviations and acronyms used in the main text and supplementary materials

**Abbreviation**	**Full Term**
AI	Artificial intelligence
AP	Aggregation propensity
CatBoost	Categorical Boosting
CGMD	Coarse-grained molecular dynamics
DL	Deep learning
LightGBM	Light Gradient Boosting Machine
logP	Hydrophilicity
LOWESS	Locally weighted scatterplot smoothing
MAE	Mean absolute error
MD	Molecular dynamics
MD2	Mixture L2-Discrepancy
ML	Machine learning
MSE	Mean square error
PRAUC	Precision-Recall area under the curve
pI	Isoelectric point
R^2	Coefficient of determination
RF	Random forest
RMSE	Root mean square error
ROCAUC	Receiver operating characteristic area under the curve
SHAP	Shapley additive explanations
SOTA	Stochastic optimization adaptive threshold acceptance
SVM	Support vector machine
TA	Threshold acceptance
UD	Uniform design
XGBoost	eXtreme Gradient Boosting

Table 2. List of variable name abbreviations and descriptions used in Supplementary Data 10

Variable	Full Variable Name	Description
A	Alanine	The fraction of alanine within the tetrapeptide sequence.
R	Arginine	The fraction of arginine within the tetrapeptide sequence.
N	Asparagine	The fraction of asparagine within the tetrapeptide sequence.
D	Aspartic acid	The fraction of aspartic acid within the tetrapeptide sequence.
C	Cysteine	The fraction of cysteine within the tetrapeptide sequence.
E	Glutamic acid	The fraction of glutamic acid within the tetrapeptide sequence.
Q	Glutamine	The fraction of glutamine within the tetrapeptide sequence.
G	Glycine	The fraction of glycine within the tetrapeptide sequence.
H	Histidine	The fraction of histidine within the tetrapeptide sequence.
I	Isoleucine	The fraction of isoleucine within the tetrapeptide sequence.
L	Leucine	The fraction of leucine within the tetrapeptide sequence.
K	Lysine	The fraction of lysine within the tetrapeptide sequence.
M	Methionine	The fraction of methionine within the tetrapeptide sequence.
F	Phenylalanine	The fraction of phenylalanine within the

		tetrapeptide sequence.
P	Proline	The fraction of proline within the tetrapeptide sequence.
S	Serine	The fraction of serine within the tetrapeptide sequence.
T	Threonine	The fraction of threonine within the tetrapeptide sequence.
W	Tryptophan	The fraction of tryptophan within the tetrapeptide sequence.
Y	Tyrosine	The fraction of tyrosine within the tetrapeptide sequence.
V	Valine	The fraction of valine within the tetrapeptide sequence.
AA	Alanine-Alanine	The fraction of the dipeptide "Alanine-Alanine" in the sequence.
RA	Arginine-Alanine	The fraction of the dipeptide "Arginine-Alanine" in the sequence.
NA	Asparagine-Alanine	The fraction of the dipeptide "Asparagine-Alanine" in the sequence.
DA	Aspartic acid-Alanine	The fraction of the dipeptide "Aspartic acid-Alanine" in the sequence.
CA	Cysteine-Alanine	The fraction of the dipeptide "Cysteine-Alanine" in the sequence.
EA	Glutamic acid-Alanine	The fraction of the dipeptide "Glutamic acid-Alanine" in the sequence.
QA	Glutamine-Alanine	The fraction of the

		dipeptide "Glutamine-Alanine" in the sequence.
GA	Glycine-Alanine	The fraction of the dipeptide "Glycine-Alanine" in the sequence.
HA	Histidine-Alanine	The fraction of the dipeptide "Histidine-Alanine" in the sequence.
IA	Isoleucine-Alanine	The fraction of the dipeptide "Isoleucine-Alanine" in the sequence.
LA	Leucine-Alanine	The fraction of the dipeptide "Leucine-Alanine" in the sequence.
KA	Lysine-Alanine	The fraction of the dipeptide "Lysine-Alanine" in the sequence.
MA	Methionine-Alanine	The fraction of the dipeptide "Methionine-Alanine" in the sequence.
FA	Phenylalanine-Alanine	The fraction of the dipeptide "Phenylalanine-Alanine" in the sequence.
PA	Proline-Alanine	The fraction of the dipeptide "Proline-Alanine" in the sequence.
SA	Serine-Alanine	The fraction of the dipeptide "Serine-Alanine" in the sequence.
TA	Threonine-Alanine	The fraction of the dipeptide "Threonine-Alanine" in the sequence.
WA	Tryptophan-Alanine	The fraction of the

		dipeptide "Tryptophan-Alanine" in the sequence.
YA	Tyrosine-Alanine	The fraction of the dipeptide "Tyrosine-Alanine" in the sequence.
VA	Valine-Alanine	The fraction of the dipeptide "Valine-Alanine" in the sequence.
AR	Alanine-Arginine	The fraction of the dipeptide "Alanine-Arginine" in the sequence.
RR	Arginine-Arginine	The fraction of the dipeptide "Arginine-Arginine" in the sequence.
NR	Asparagine-Arginine	The fraction of the dipeptide "Asparagine-Arginine" in the sequence.
DR	Aspartic acid-Arginine	The fraction of the dipeptide "Aspartic acid-Arginine" in the sequence.
CR	Cysteine-Arginine	The fraction of the dipeptide "Cysteine-Arginine" in the sequence.
ER	Glutamic acid-Arginine	The fraction of the dipeptide "Glutamic acid-Arginine" in the sequence.
QR	Glutamine-Arginine	The fraction of the dipeptide "Glutamine-Arginine" in the sequence.
GR	Glycine-Arginine	The fraction of the dipeptide "Glycine-Arginine" in the sequence.
HR	Histidine-Arginine	The fraction of the

		dipeptide "Histidine-Arginine" in the sequence.
IR	Isoleucine-Arginine	The fraction of the dipeptide "Isoleucine-Arginine" in the sequence.
LR	Leucine-Arginine	The fraction of the dipeptide "Leucine-Arginine" in the sequence.
KR	Lysine-Arginine	The fraction of the dipeptide "Lysine-Arginine" in the sequence.
MR	Methionine-Arginine	The fraction of the dipeptide "Methionine-Arginine" in the sequence.
FR	Phenylalanine-Arginine	The fraction of the dipeptide "Phenylalanine-Arginine" in the sequence.
PR	Proline-Arginine	The fraction of the dipeptide "Proline-Arginine" in the sequence.
SR	Serine-Arginine	The fraction of the dipeptide "Serine-Arginine" in the sequence.
TR	Threonine-Arginine	The fraction of the dipeptide "Threonine-Arginine" in the sequence.
WR	Tryptophan-Arginine	The fraction of the dipeptide "Tryptophan-Arginine" in the sequence.
YR	Tyrosine-Arginine	The fraction of the dipeptide "Tyrosine-Arginine" in the sequence.
VR	Valine-Arginine	The fraction of the

		dipeptide "Valine-Arginine" in the sequence.
AN	Alanine-Asparagine	The fraction of the dipeptide "Alanine-Asparagine" in the sequence.
RN	Arginine-Asparagine	The fraction of the dipeptide "Arginine-Asparagine" in the sequence.
NN	Asparagine-Asparagine	The fraction of the dipeptide "Asparagine-Asparagine" in the sequence.
DN	Aspartic acid-Asparagine	The fraction of the dipeptide "Aspartic acid-Asparagine" in the sequence.
CN	Cysteine-Asparagine	The fraction of the dipeptide "Cysteine-Asparagine" in the sequence.
EN	Glutamic acid-Asparagine	The fraction of the dipeptide "Glutamic acid-Asparagine" in the sequence.
QN	Glutamine-Asparagine	The fraction of the dipeptide "Glutamine-Asparagine" in the sequence.
GN	Glycine-Asparagine	The fraction of the dipeptide "Glycine-Asparagine" in the sequence.
HN	Histidine-Asparagine	The fraction of the dipeptide "Histidine-Asparagine" in the sequence.
IN	Isoleucine-Asparagine	The fraction of the dipeptide "Isoleucine-Asparagine" in the sequence.
LN	Leucine-Asparagine	The fraction of the

		dipeptide "Leucine-Asparagine" in the sequence.
KN	Lysine-Asparagine	The fraction of the dipeptide "Lysine-Asparagine" in the sequence.
MN	Methionine-Asparagine	The fraction of the dipeptide "Methionine-Asparagine" in the sequence.
FN	Phenylalanine-Asparagine	The fraction of the dipeptide "Phenylalanine-Asparagine" in the sequence.
PN	Proline-Asparagine	The fraction of the dipeptide "Proline-Asparagine" in the sequence.
SN	Serine-Asparagine	The fraction of the dipeptide "Serine-Asparagine" in the sequence.
TN	Threonine-Asparagine	The fraction of the dipeptide "Threonine-Asparagine" in the sequence.
WN	Tryptophan-Asparagine	The fraction of the dipeptide "Tryptophan-Asparagine" in the sequence.
YN	Tyrosine-Asparagine	The fraction of the dipeptide "Tyrosine-Asparagine" in the sequence.
VN	Valine-Asparagine	The fraction of the dipeptide "Valine-Asparagine" in the sequence.
AD	Alanine-Aspartic acid	The fraction of the dipeptide "Alanine-Aspartic acid" in the sequence.
RD	Arginine-Aspartic acid	The fraction of the

		dipeptide "Arginine-Aspartic acid" in the sequence.
ND	Asparagine-Aspartic acid	The fraction of the dipeptide "Asparagine-Aspartic acid" in the sequence.
DD	Aspartic acid-Aspartic acid	The fraction of the dipeptide "Aspartic acid-Aspartic acid" in the sequence.
CD	Cysteine-Aspartic acid	The fraction of the dipeptide "Cysteine-Aspartic acid" in the sequence.
ED	Glutamic acid-Aspartic acid	The fraction of the dipeptide "Glutamic acid-Aspartic acid" in the sequence.
QD	Glutamine-Aspartic acid	The fraction of the dipeptide "Glutamine-Aspartic acid" in the sequence.
GD	Glycine-Aspartic acid	The fraction of the dipeptide "Glycine-Aspartic acid" in the sequence.
HD	Histidine-Aspartic acid	The fraction of the dipeptide "Histidine-Aspartic acid" in the sequence.
ID	Isoleucine-Aspartic acid	The fraction of the dipeptide "Isoleucine-Aspartic acid" in the sequence.
LD	Leucine-Aspartic acid	The fraction of the dipeptide "Leucine-Aspartic acid" in the sequence.
KD	Lysine-Aspartic acid	The fraction of the dipeptide "Lysine-Aspartic acid" in the sequence.
MD	Methionine-Aspartic acid	The fraction of the dipeptide

		"Methionine-Aspartic acid" in the sequence.
FD	Phenylalanine-Aspartic acid	The fraction of the dipeptide "Phenylalanine-Aspartic acid" in the sequence.
PD	Proline-Aspartic acid	The fraction of the dipeptide "Proline-Aspartic acid" in the sequence.
SD	Serine-Aspartic acid	The fraction of the dipeptide "Serine-Aspartic acid" in the sequence.
TD	Threonine-Aspartic acid	The fraction of the dipeptide "Threonine-Aspartic acid" in the sequence.
WD	Tryptophan-Aspartic acid	The fraction of the dipeptide "Tryptophan-Aspartic acid" in the sequence.
YD	Tyrosine-Aspartic acid	The fraction of the dipeptide "Tyrosine-Aspartic acid" in the sequence.
VD	Valine-Aspartic acid	The fraction of the dipeptide "Valine-Aspartic acid" in the sequence.
AC	Alanine-Cysteine	The fraction of the dipeptide "Alanine-Cysteine" in the sequence.
RC	Arginine-Cysteine	The fraction of the dipeptide "Arginine-Cysteine" in the sequence.
NC	Asparagine-Cysteine	The fraction of the dipeptide "Asparagine-Cysteine" in the sequence.
DC	Aspartic acid-Cysteine	The fraction of the dipeptide "Aspartic acid-Cysteine" in the sequence.

CC	Cysteine-Cysteine	The fraction of the dipeptide "Cysteine-Cysteine" in the sequence.
EC	Glutamic acid-Cysteine	The fraction of the dipeptide "Glutamic acid-Cysteine" in the sequence.
QC	Glutamine-Cysteine	The fraction of the dipeptide "Glutamine-Cysteine" in the sequence.
GC	Glycine-Cysteine	The fraction of the dipeptide "Glycine-Cysteine" in the sequence.
HC	Histidine-Cysteine	The fraction of the dipeptide "Histidine-Cysteine" in the sequence.
IC	Isoleucine-Cysteine	The fraction of the dipeptide "Isoleucine-Cysteine" in the sequence.
LC	Leucine-Cysteine	The fraction of the dipeptide "Leucine-Cysteine" in the sequence.
KC	Lysine-Cysteine	The fraction of the dipeptide "Lysine-Cysteine" in the sequence.
MC	Methionine-Cysteine	The fraction of the dipeptide "Methionine-Cysteine" in the sequence.
FC	Phenylalanine-Cysteine	The fraction of the dipeptide "Phenylalanine-Cysteine" in the sequence.
PC	Proline-Cysteine	The fraction of the dipeptide "Proline-Cysteine" in the sequence.

SC	Serine-Cysteine	The fraction of the dipeptide "Serine-Cysteine" in the sequence.
TC	Threonine-Cysteine	The fraction of the dipeptide "Threonine-Cysteine" in the sequence.
WC	Tryptophan-Cysteine	The fraction of the dipeptide "Tryptophan-Cysteine" in the sequence.
YC	Tyrosine-Cysteine	The fraction of the dipeptide "Tyrosine-Cysteine" in the sequence.
VC	Valine-Cysteine	The fraction of the dipeptide "Valine-Cysteine" in the sequence.
AE	Alanine-Glutamic acid	The fraction of the dipeptide "Alanine-Glutamic acid" in the sequence.
RE	Arginine-Glutamic acid	The fraction of the dipeptide "Arginine-Glutamic acid" in the sequence.
NE	Asparagine-Glutamic acid	The fraction of the dipeptide "Asparagine-Glutamic acid" in the sequence.
DE	Aspartic acid-Glutamic acid	The fraction of the dipeptide "Aspartic acid-Glutamic acid" in the sequence.
CE	Cysteine-Glutamic acid	The fraction of the dipeptide "Cysteine-Glutamic acid" in the sequence.
EE	Glutamic acid-Glutamic acid	The fraction of the dipeptide "Glutamic acid-Glutamic acid" in the sequence.

QE	Glutamine-Glutamic acid	The fraction of the dipeptide "Glutamine-Glutamic acid" in the sequence.
GE	Glycine-Glutamic acid	The fraction of the dipeptide "Glycine-Glutamic acid" in the sequence.
HE	Histidine-Glutamic acid	The fraction of the dipeptide "Histidine-Glutamic acid" in the sequence.
IE	Isoleucine-Glutamic acid	The fraction of the dipeptide "Isoleucine-Glutamic acid" in the sequence.
LE	Leucine-Glutamic acid	The fraction of the dipeptide "Leucine-Glutamic acid" in the sequence.
KE	Lysine-Glutamic acid	The fraction of the dipeptide "Lysine-Glutamic acid" in the sequence.
ME	Methionine-Glutamic acid	The fraction of the dipeptide "Methionine-Glutamic acid" in the sequence.
FE	Phenylalanine-Glutamic acid	The fraction of the dipeptide "Phenylalanine-Glutamic acid" in the sequence.
PE	Proline-Glutamic acid	The fraction of the dipeptide "Proline-Glutamic acid" in the sequence.
SE	Serine-Glutamic acid	The fraction of the dipeptide "Serine-Glutamic acid" in the sequence.
TE	Threonine-Glutamic acid	The fraction of the dipeptide "Threonine-Glutamic acid" in the sequence.

WE	Tryptophan-Glutamic acid	The fraction of the dipeptide "Tryptophan-Glutamic acid" in the sequence.
YE	Tyrosine-Glutamic acid	The fraction of the dipeptide "Tyrosine-Glutamic acid" in the sequence.
VE	Valine-Glutamic acid	The fraction of the dipeptide "Valine-Glutamic acid" in the sequence.
GE	Glycine-Glutamic acid	The fraction of the dipeptide "Glycine-Glutamic acid" in the sequence.
HE	Histidine-Glutamic acid	The fraction of the dipeptide "Histidine-Glutamic acid" in the sequence.
IE	Isoleucine-Glutamic acid	The fraction of the dipeptide "Isoleucine-Glutamic acid" in the sequence.
LE	Leucine-Glutamic acid	The fraction of the dipeptide "Leucine-Glutamic acid" in the sequence.
KE	Lysine-Glutamic acid	The fraction of the dipeptide "Lysine-Glutamic acid" in the sequence.
ME	Methionine-Glutamic acid	The fraction of the dipeptide "Methionine-Glutamic acid" in the sequence.
FE	Phenylalanine-Glutamic acid	The fraction of the dipeptide "Phenylalanine-Glutamic acid" in the sequence.
PE	Proline-Glutamic acid	The fraction of the dipeptide "Proline-Glutamic acid" in the sequence.

SE	Serine-Glutamic acid	The fraction of the dipeptide "Serine-Glutamic acid" in the sequence.
TE	Threonine-Glutamic acid	The fraction of the dipeptide "Threonine-Glutamic acid" in the sequence.
WE	Tryptophan-Glutamic acid	The fraction of the dipeptide "Tryptophan-Glutamic acid" in the sequence.
YE	Tyrosine-Glutamic acid	The fraction of the dipeptide "Tyrosine-Glutamic acid" in the sequence.
VE	Valine-Glutamic acid	The fraction of the dipeptide "Valine-Glutamic acid" in the sequence.
AQ	Alanine-Glutamine	The fraction of the dipeptide "Alanine-Glutamine" in the sequence.
RQ	Arginine-Glutamine	The fraction of the dipeptide "Arginine-Glutamine" in the sequence.
NQ	Asparagine-Glutamine	The fraction of the dipeptide "Asparagine-Glutamine" in the sequence.
DQ	Aspartic acid-Glutamine	The fraction of the dipeptide "Aspartic acid-Glutamine" in the sequence.
CQ	Cysteine-Glutamine	The fraction of the dipeptide "Cysteine-Glutamine" in the sequence.
EQ	Glutamic acid-Glutamine	The fraction of the dipeptide "Glutamic acid-Glutamine" in the sequence.

QQ	Glutamine-Glutamine	The fraction of the dipeptide "Glutamine-Glutamine" in the sequence.
GQ	Glycine-Glutamine	The fraction of the dipeptide "Glycine-Glutamine" in the sequence.
HQ	Histidine-Glutamine	The fraction of the dipeptide "Histidine-Glutamine" in the sequence.
IQ	Isoleucine-Glutamine	The fraction of the dipeptide "Isoleucine-Glutamine" in the sequence.
LQ	Leucine-Glutamine	The fraction of the dipeptide "Leucine-Glutamine" in the sequence.
KQ	Lysine-Glutamine	The fraction of the dipeptide "Lysine-Glutamine" in the sequence.
MQ	Methionine-Glutamine	The fraction of the dipeptide "Methionine-Glutamine" in the sequence.
FQ	Phenylalanine-Glutamine	The fraction of the dipeptide "Phenylalanine-Glutamine" in the sequence.
PQ	Proline-Glutamine	The fraction of the dipeptide "Proline-Glutamine" in the sequence.
SQ	Serine-Glutamine	The fraction of the dipeptide "Serine-Glutamine" in the sequence.
TQ	Threonine-Glutamine	The fraction of the dipeptide "Threonine-Glutamine" in the sequence.

WQ	Tryptophan-Glutamine	The fraction of the dipeptide "Tryptophan-Glutamine" in the sequence.
YQ	Tyrosine-Glutamine	The fraction of the dipeptide "Tyrosine-Glutamine" in the sequence.
VQ	Valine-Glutamine	The fraction of the dipeptide "Valine-Glutamine" in the sequence.
AG	Alanine-Glycine	The fraction of the dipeptide "Alanine-Glycine" in the sequence.
RG	Arginine-Glycine	The fraction of the dipeptide "Arginine-Glycine" in the sequence.
NG	Asparagine-Glycine	The fraction of the dipeptide "Asparagine-Glycine" in the sequence.
DG	Aspartic acid-Glycine	The fraction of the dipeptide "Aspartic acid-Glycine" in the sequence.
CG	Cysteine-Glycine	The fraction of the dipeptide "Cysteine-Glycine" in the sequence.
EG	Glutamic acid-Glycine	The fraction of the dipeptide "Glutamic acid-Glycine" in the sequence.
QG	Glutamine-Glycine	The fraction of the dipeptide "Glutamine-Glycine" in the sequence.
GG	Glycine-Glycine	The fraction of the dipeptide "Glycine-Glycine" in the sequence.

HG	Histidine-Glycine	The fraction of the dipeptide "Histidine-Glycine" in the sequence.
IG	Isoleucine-Glycine	The fraction of the dipeptide "Isoleucine-Glycine" in the sequence.
LG	Leucine-Glycine	The fraction of the dipeptide "Leucine-Glycine" in the sequence.
KG	Lysine-Glycine	The fraction of the dipeptide "Lysine-Glycine" in the sequence.
MG	Methionine-Glycine	The fraction of the dipeptide "Methionine-Glycine" in the sequence.
FG	Phenylalanine-Glycine	The fraction of the dipeptide "Phenylalanine-Glycine" in the sequence.
PG	Proline-Glycine	The fraction of the dipeptide "Proline-Glycine" in the sequence.
SG	Serine-Glycine	The fraction of the dipeptide "Serine-Glycine" in the sequence.
TG	Threonine-Glycine	The fraction of the dipeptide "Threonine-Glycine" in the sequence.
WG	Tryptophan-Glycine	The fraction of the dipeptide "Tryptophan-Glycine" in the sequence.
YG	Tyrosine-Glycine	The fraction of the dipeptide "Tyrosine-Glycine" in the sequence.

VG	Valine-Glycine	The fraction of the dipeptide "Valine-Glycine" in the sequence.
AH	Alanine-Histidine	The fraction of the dipeptide "Alanine-Histidine" in the sequence.
RH	Arginine-Histidine	The fraction of the dipeptide "Arginine-Histidine" in the sequence.
NH	Asparagine-Histidine	The fraction of the dipeptide "Asparagine-Histidine" in the sequence.
DH	Aspartic acid-Histidine	The fraction of the dipeptide "Aspartic acid-Histidine" in the sequence.
CH	Cysteine-Histidine	The fraction of the dipeptide "Cysteine-Histidine" in the sequence.
EH	Glutamic acid-Histidine	The fraction of the dipeptide "Glutamic acid-Histidine" in the sequence.
QH	Glutamine-Histidine	The fraction of the dipeptide "Glutamine-Histidine" in the sequence.
GH	Glycine-Histidine	The fraction of the dipeptide "Glycine-Histidine" in the sequence.
HH	Histidine-Histidine	The fraction of the dipeptide "Histidine-Histidine" in the sequence.
IH	Isoleucine-Histidine	The fraction of the dipeptide "Isoleucine-Histidine" in the sequence.

LH	Leucine-Histidine	The fraction of the dipeptide "Leucine-Histidine" in the sequence.
KH	Lysine-Histidine	The fraction of the dipeptide "Lysine-Histidine" in the sequence.
MH	Methionine-Histidine	The fraction of the dipeptide "Methionine-Histidine" in the sequence.
FH	Phenylalanine-Histidine	The fraction of the dipeptide "Phenylalanine-Histidine" in the sequence.
PH	Proline-Histidine	The fraction of the dipeptide "Proline-Histidine" in the sequence.
SH	Serine-Histidine	The fraction of the dipeptide "Serine-Histidine" in the sequence.
TH	Threonine-Histidine	The fraction of the dipeptide "Threonine-Histidine" in the sequence.
WH	Tryptophan-Histidine	The fraction of the dipeptide "Tryptophan-Histidine" in the sequence.
YH	Tyrosine-Histidine	The fraction of the dipeptide "Tyrosine-Histidine" in the sequence.
VH	Valine-Histidine	The fraction of the dipeptide "Valine-Histidine" in the sequence.
AI	Alanine-Isoleucine	The fraction of the dipeptide "Alanine-Isoleucine" in the sequence.

RI	Arginine-Isoleucine	The fraction of the dipeptide "Arginine-Isoleucine" in the sequence.
NI	Asparagine-Isoleucine	The fraction of the dipeptide "Asparagine-Isoleucine" in the sequence.
DI	Aspartic acid-Isoleucine	The fraction of the dipeptide "Aspartic acid-Isoleucine" in the sequence.
CI	Cysteine-Isoleucine	The fraction of the dipeptide "Cysteine-Isoleucine" in the sequence.
EI	Glutamic acid-Isoleucine	The fraction of the dipeptide "Glutamic acid-Isoleucine" in the sequence.
QI	Glutamine-Isoleucine	The fraction of the dipeptide "Glutamine-Isoleucine" in the sequence.
GI	Glycine-Isoleucine	The fraction of the dipeptide "Glycine-Isoleucine" in the sequence.
HI	Histidine-Isoleucine	The fraction of the dipeptide "Histidine-Isoleucine" in the sequence.
II	Isoleucine-Isoleucine	The fraction of the dipeptide "Isoleucine-Isoleucine" in the sequence.
LI	Leucine-Isoleucine	The fraction of the dipeptide "Leucine-Isoleucine" in the sequence.
KI	Lysine-Isoleucine	The fraction of the dipeptide "Lysine-Isoleucine" in the sequence.

MI	Methionine-Isoleucine	The fraction of the dipeptide "Methionine-Isoleucine" in the sequence.
FI	Phenylalanine-Isoleucin e	The fraction of the dipeptide "Phenylalanine-Isoleucine " in the sequence.
PI	Proline-Isoleucine	The fraction of the dipeptide "Proline-Isoleucine" in the sequence.
SI	Serine-Isoleucine	The fraction of the dipeptide "Serine-Isoleucine" in the sequence.
TI	Threonine-Isoleucine	The fraction of the dipeptide "Threonine-Isoleucine" in the sequence.
WI	Tryptophan-Isoleucine	The fraction of the dipeptide "Tryptophan-Isoleucine" in the sequence.
YI	Tyrosine-Isoleucine	The fraction of the dipeptide "Tyrosine-Isoleucine" in the sequence.
VI	Valine-Isoleucine	The fraction of the dipeptide "Valine-Isoleucine" in the sequence.
AL	Alanine-Leucine	The fraction of the dipeptide "Alanine-Leucine" in the sequence.
RL	Arginine-Leucine	The fraction of the dipeptide "Arginine-Leucine" in the sequence.
NL	Asparagine-Leucine	The fraction of the dipeptide "Asparagine-Leucine" in the sequence.

DL	Aspartic acid-Leucine	The fraction of the dipeptide "Aspartic acid-Leucine" in the sequence.
CL	Cysteine-Leucine	The fraction of the dipeptide "Cysteine-Leucine" in the sequence.
EL	Glutamic acid-Leucine	The fraction of the dipeptide "Glutamic acid-Leucine" in the sequence.
QL	Glutamine-Leucine	The fraction of the dipeptide "Glutamine-Leucine" in the sequence.
GL	Glycine-Leucine	The fraction of the dipeptide "Glycine-Leucine" in the sequence.
HL	Histidine-Leucine	The fraction of the dipeptide "Histidine-Leucine" in the sequence.
IL	Isoleucine-Leucine	The fraction of the dipeptide "Isoleucine-Leucine" in the sequence.
LL	Leucine-Leucine	The fraction of the dipeptide "Leucine-Leucine" in the sequence.
KL	Lysine-Leucine	The fraction of the dipeptide "Lysine-Leucine" in the sequence.
ML	Methionine-Leucine	The fraction of the dipeptide "Methionine-Leucine" in the sequence.
FL	Phenylalanine-Leucine	The fraction of the dipeptide "Phenylalanine-Leucine" in the sequence.

PL	Proline-Leucine	The fraction of the dipeptide "Proline-Leucine" in the sequence.
SL	Serine-Leucine	The fraction of the dipeptide "Serine-Leucine" in the sequence.
TL	Threonine-Leucine	The fraction of the dipeptide "Threonine-Leucine" in the sequence.
WL	Tryptophan-Leucine	The fraction of the dipeptide "Tryptophan-Leucine" in the sequence.
YL	Tyrosine-Leucine	The fraction of the dipeptide "Tyrosine-Leucine" in the sequence.
VL	Valine-Leucine	The fraction of the dipeptide "Valine-Leucine" in the sequence.
AK	Alanine-Lysine	The fraction of the dipeptide "Alanine-Lysine" in the sequence.
RK	Arginine-Lysine	The fraction of the dipeptide "Arginine-Lysine" in the sequence.
NK	Asparagine-Lysine	The fraction of the dipeptide "Asparagine-Lysine" in the sequence.
DK	Aspartic acid-Lysine	The fraction of the dipeptide "Aspartic acid-Lysine" in the sequence.
CK	Cysteine-Lysine	The fraction of the dipeptide "Cysteine-Lysine" in the sequence.

EK	Glutamic acid-Lysine	The fraction of the dipeptide "Glutamic acid-Lysine" in the sequence.
QK	Glutamine-Lysine	The fraction of the dipeptide "Glutamine-Lysine" in the sequence.
GK	Glycine-Lysine	The fraction of the dipeptide "Glycine-Lysine" in the sequence.
HK	Histidine-Lysine	The fraction of the dipeptide "Histidine-Lysine" in the sequence.
IK	Isoleucine-Lysine	The fraction of the dipeptide "Isoleucine-Lysine" in the sequence.
LK	Leucine-Lysine	The fraction of the dipeptide "Leucine-Lysine" in the sequence.
KK	Lysine-Lysine	The fraction of the dipeptide "Lysine-Lysine" in the sequence.
MK	Methionine-Lysine	The fraction of the dipeptide "Methionine-Lysine" in the sequence.
FK	Phenylalanine-Lysine	The fraction of the dipeptide "Phenylalanine-Lysine" in the sequence.
PK	Proline-Lysine	The fraction of the dipeptide "Proline-Lysine" in the sequence.
SK	Serine-Lysine	The fraction of the dipeptide "Serine-Lysine" in the sequence.
TK	Threonine-Lysine	The fraction of the dipeptide "Threonine-Lysine" in the

		sequence.
WK	Tryptophan-Lysine	The fraction of the dipeptide "Tryptophan-Lysine" in the sequence.
YK	Tyrosine-Lysine	The fraction of the dipeptide "Tyrosine-Lysine" in the sequence.
VK	Valine-Lysine	The fraction of the dipeptide "Valine-Lysine" in the sequence.
AM	Alanine-Methionine	The fraction of the dipeptide "Alanine-Methionine" in the sequence.
RM	Arginine-Methionine	The fraction of the dipeptide "Arginine-Methionine" in the sequence.
NM	Asparagine-Methionine	The fraction of the dipeptide "Asparagine-Methionine" in the sequence.
DM	Aspartic acid-Methionine	The fraction of the dipeptide "Aspartic acid-Methionine" in the sequence.
CM	Cysteine-Methionine	The fraction of the dipeptide "Cysteine-Methionine" in the sequence.
EM	Glutamic acid-Methionine	The fraction of the dipeptide "Glutamic acid-Methionine" in the sequence.
QM	Glutamine-Methionine	The fraction of the dipeptide "Glutamine-Methionine" in the sequence.
GM	Glycine-Methionine	The fraction of the dipeptide "Glycine-Methionine" in the sequence.

HM	Histidine-Methionine	The fraction of the dipeptide "Histidine-Methionine" in the sequence.
IM	Isoleucine-Methionine	The fraction of the dipeptide "Isoleucine-Methionine" in the sequence.
LM	Leucine-Methionine	The fraction of the dipeptide "Leucine-Methionine" in the sequence.
KM	Lysine-Methionine	The fraction of the dipeptide "Lysine-Methionine" in the sequence.
MM	Methionine-Methionine	The fraction of the dipeptide "Methionine-Methionine" in the sequence.
FM	Phenylalanine-Methionine	The fraction of the dipeptide "Phenylalanine-Methionine" in the sequence.
PM	Proline-Methionine	The fraction of the dipeptide "Proline-Methionine" in the sequence.
SM	Serine-Methionine	The fraction of the dipeptide "Serine-Methionine" in the sequence.
TM	Threonine-Methionine	The fraction of the dipeptide "Threonine-Methionine" in the sequence.
WM	Tryptophan-Methionine	The fraction of the dipeptide "Tryptophan-Methionine" in the sequence.
YM	Tyrosine-Methionine	The fraction of the dipeptide "Tyrosine-Methionine" in the sequence.

VM	Valine-Methionine	The fraction of the dipeptide "Valine-Methionine" in the sequence.
AF	Alanine-Phenylalanine	The fraction of the dipeptide "Alanine-Phenylalanine" in the sequence.
RF	Arginine-Phenylalanine	The fraction of the dipeptide "Arginine-Phenylalanine" in the sequence.
NF	Asparagine-Phenylalanine	The fraction of the dipeptide "Asparagine-Phenylalanine" in the sequence.
DF	Aspartic acid-Phenylalanine	The fraction of the dipeptide "Aspartic acid-Phenylalanine" in the sequence.
CF	Cysteine-Phenylalanine	The fraction of the dipeptide "Cysteine-Phenylalanine" in the sequence.
EF	Glutamic acid-Phenylalanine	The fraction of the dipeptide "Glutamic acid-Phenylalanine" in the sequence.
QF	Glutamine-Phenylalanine	The fraction of the dipeptide "Glutamine-Phenylalanine" in the sequence.
GF	Glycine-Phenylalanine	The fraction of the dipeptide "Glycine-Phenylalanine" in the sequence.
HF	Histidine-Phenylalanine	The fraction of the dipeptide "Histidine-Phenylalanine" in the sequence.
IF	Isoleucine-Phenylalanine	The fraction of the dipeptide "Isoleucine-Phenylalanine" in the sequence.

LF	Leucine-Phenylalanine	The fraction of the dipeptide "Leucine-Phenylalanine" in the sequence.
KF	Lysine-Phenylalanine	The fraction of the dipeptide "Lysine-Phenylalanine" in the sequence.
MF	Methionine-Phenylalanine	The fraction of the dipeptide "Methionine-Phenylalanine" in the sequence.
FF	Phenylalanine-Phenylalanine	The fraction of the dipeptide "Phenylalanine-Phenylalanine" in the sequence.
PF	Proline-Phenylalanine	The fraction of the dipeptide "Proline-Phenylalanine" in the sequence.
SF	Serine-Phenylalanine	The fraction of the dipeptide "Serine-Phenylalanine" in the sequence.
TF	Threonine-Phenylalanine	The fraction of the dipeptide "Threonine-Phenylalanine" in the sequence.
WF	Tryptophan-Phenylalanine	The fraction of the dipeptide "Tryptophan-Phenylalanine" in the sequence.
YF	Tyrosine-Phenylalanine	The fraction of the dipeptide "Tyrosine-Phenylalanine" in the sequence.
VF	Valine-Phenylalanine	The fraction of the dipeptide "Valine-Phenylalanine" in the sequence.
AP	Alanine-Proline	The fraction of the dipeptide "Alanine-Proline" in the sequence.

RP	Arginine-Proline	The fraction of the dipeptide "Arginine-Proline" in the sequence.
NP	Asparagine-Proline	The fraction of the dipeptide "Asparagine-Proline" in the sequence.
DP	Aspartic acid-Proline	The fraction of the dipeptide "Aspartic acid-Proline" in the sequence.
CP	Cysteine-Proline	The fraction of the dipeptide "Cysteine-Proline" in the sequence.
EP	Glutamic acid-Proline	The fraction of the dipeptide "Glutamic acid-Proline" in the sequence.
QP	Glutamine-Proline	The fraction of the dipeptide "Glutamine-Proline" in the sequence.
GP	Glycine-Proline	The fraction of the dipeptide "Glycine-Proline" in the sequence.
HP	Histidine-Proline	The fraction of the dipeptide "Histidine-Proline" in the sequence.
IP	Isoleucine-Proline	The fraction of the dipeptide "Isoleucine-Proline" in the sequence.
LP	Leucine-Proline	The fraction of the dipeptide "Leucine-Proline" in the sequence.
KP	Lysine-Proline	The fraction of the dipeptide "Lysine-Proline" in the sequence.
MP	Methionine-Proline	The fraction of the

		dipeptide "Methionine-Proline" in the sequence.
FP	Phenylalanine-Proline	The fraction of the dipeptide "Phenylalanine-Proline" in the sequence.
PP	Proline-Proline	The fraction of the dipeptide "Proline-Proline" in the sequence.
SP	Serine-Proline	The fraction of the dipeptide "Serine-Proline" in the sequence.
TP	Threonine-Proline	The fraction of the dipeptide "Threonine-Proline" in the sequence.
WP	Tryptophan-Proline	The fraction of the dipeptide "Tryptophan-Proline" in the sequence.
YP	Tyrosine-Proline	The fraction of the dipeptide "Tyrosine-Proline" in the sequence.
VP	Valine-Proline	The fraction of the dipeptide "Valine-Proline" in the sequence.
AS	Alanine-Serine	The fraction of the dipeptide "Alanine-Serine" in the sequence.
RS	Arginine-Serine	The fraction of the dipeptide "Arginine-Serine" in the sequence.
NS	Asparagine-Serine	The fraction of the dipeptide "Asparagine-Serine" in the sequence.
DS	Aspartic acid-Serine	The fraction of the dipeptide "Aspartic acid-Serine" in the

		sequence.
CS	Cysteine-Serine	The fraction of the dipeptide "Cysteine-Serine" in the sequence.
ES	Glutamic acid-Serine	The fraction of the dipeptide "Glutamic acid-Serine" in the sequence.
QS	Glutamine-Serine	The fraction of the dipeptide "Glutamine-Serine" in the sequence.
GS	Glycine-Serine	The fraction of the dipeptide "Glycine-Serine" in the sequence.
HS	Histidine-Serine	The fraction of the dipeptide "Histidine-Serine" in the sequence.
IS	Isoleucine-Serine	The fraction of the dipeptide "Isoleucine-Serine" in the sequence.
LS	Leucine-Serine	The fraction of the dipeptide "Leucine-Serine" in the sequence.
KS	Lysine-Serine	The fraction of the dipeptide "Lysine-Serine" in the sequence.
MS	Methionine-Serine	The fraction of the dipeptide "Methionine-Serine" in the sequence.
FS	Phenylalanine-Serine	The fraction of the dipeptide "Phenylalanine-Serine" in the sequence.
PS	Proline-Serine	The fraction of the dipeptide "Proline-Serine" in the sequence.
SS	Serine-Serine	The fraction of the

		dipeptide "Serine-Serine" in the sequence.
TS	Threonine-Serine	The fraction of the dipeptide "Threonine-Serine" in the sequence.
WS	Tryptophan-Serine	The fraction of the dipeptide "Tryptophan-Serine" in the sequence.
YS	Tyrosine-Serine	The fraction of the dipeptide "Tyrosine-Serine" in the sequence.
VS	Valine-Serine	The fraction of the dipeptide "Valine-Serine" in the sequence.
SP	Serine-Proline	The fraction of the dipeptide "Serine-Proline" in the sequence.
TP	Threonine-Proline	The fraction of the dipeptide "Threonine-Proline" in the sequence.
WP	Tryptophan-Proline	The fraction of the dipeptide "Tryptophan-Proline" in the sequence.
YP	Tyrosine-Proline	The fraction of the dipeptide "Tyrosine-Proline" in the sequence.
VP	Valine-Proline	The fraction of the dipeptide "Valine-Proline" in the sequence.
AS	Alanine-Serine	The fraction of the dipeptide "Alanine-Serine" in the sequence.
RS	Arginine-Serine	The fraction of the dipeptide "Arginine-Serine" in the sequence.
NS	Asparagine-Serine	The fraction of the

		dipeptide "Asparagine-Serine" in the sequence.
DS	Aspartic acid-Serine	The fraction of the dipeptide "Aspartic acid-Serine" in the sequence.
CS	Cysteine-Serine	The fraction of the dipeptide "Cysteine-Serine" in the sequence.
ES	Glutamic acid-Serine	The fraction of the dipeptide "Glutamic acid-Serine" in the sequence.
QS	Glutamine-Serine	The fraction of the dipeptide "Glutamine-Serine" in the sequence.
GS	Glycine-Serine	The fraction of the dipeptide "Glycine-Serine" in the sequence.
HS	Histidine-Serine	The fraction of the dipeptide "Histidine-Serine" in the sequence.
IS	Isoleucine-Serine	The fraction of the dipeptide "Isoleucine-Serine" in the sequence.
LS	Leucine-Serine	The fraction of the dipeptide "Leucine-Serine" in the sequence.
KS	Lysine-Serine	The fraction of the dipeptide "Lysine-Serine" in the sequence.
MS	Methionine-Serine	The fraction of the dipeptide "Methionine-Serine" in the sequence.
FS	Phenylalanine-Serine	The fraction of the dipeptide

		"Phenylalanine-Serine" in the sequence.
PS	Proline-Serine	The fraction of the dipeptide "Proline-Serine" in the sequence.
SS	Serine-Serine	The fraction of the dipeptide "Serine-Serine" in the sequence.
TS	Threonine-Serine	The fraction of the dipeptide "Threonine-Serine" in the sequence.
WS	Tryptophan-Serine	The fraction of the dipeptide "Tryptophan-Serine" in the sequence.
YS	Tyrosine-Serine	The fraction of the dipeptide "Tyrosine-Serine" in the sequence.
VS	Valine-Serine	The fraction of the dipeptide "Valine-Serine" in the sequence.
AT	Alanine-Threonine	The fraction of the dipeptide "Alanine-Threonine" in the sequence.
RT	Arginine-Threonine	The fraction of the dipeptide "Arginine-Threonine" in the sequence.
NT	Asparagine-Threonine	The fraction of the dipeptide "Asparagine-Threonine" in the sequence.
DT	Aspartic acid-Threonine	The fraction of the dipeptide "Aspartic acid-Threonine" in the sequence.
CT	Cysteine-Threonine	The fraction of the dipeptide "Cysteine-Threonine" in the sequence.
ET	Glutamic	The fraction of the

	acid-Threonine	dipeptide "Glutamic acid-Threonine" in the sequence.
QT	Glutamine-Threonine	The fraction of the dipeptide "Glutamine-Threonine" in the sequence.
GT	Glycine-Threonine	The fraction of the dipeptide "Glycine-Threonine" in the sequence.
HT	Histidine-Threonine	The fraction of the dipeptide "Histidine-Threonine" in the sequence.
IT	Isoleucine-Threonine	The fraction of the dipeptide "Isoleucine-Threonine" in the sequence.
LT	Leucine-Threonine	The fraction of the dipeptide "Leucine-Threonine" in the sequence.
KT	Lysine-Threonine	The fraction of the dipeptide "Lysine-Threonine" in the sequence.
MT	Methionine-Threonine	The fraction of the dipeptide "Methionine-Threonine" in the sequence.
FT	Phenylalanine-Threonine	The fraction of the dipeptide "Phenylalanine-Threonine" in the sequence.
PT	Proline-Threonine	The fraction of the dipeptide "Proline-Threonine" in the sequence.
ST	Serine-Threonine	The fraction of the dipeptide "Serine-Threonine" in the sequence.
TT	Threonine-Threonine	The fraction of the

		dipeptide "Threonine-Threonine" in the sequence.
WT	Tryptophan-Threonine	The fraction of the dipeptide "Tryptophan-Threonine" in the sequence.
YT	Tyrosine-Threonine	The fraction of the dipeptide "Tyrosine-Threonine" in the sequence.
VT	Valine-Threonine	The fraction of the dipeptide "Valine-Threonine" in the sequence.
AW	Alanine-Tryptophan	The fraction of the dipeptide "Alanine-Tryptophan" in the sequence.
RW	Arginine-Tryptophan	The fraction of the dipeptide "Arginine-Tryptophan" in the sequence.
NW	Asparagine-Tryptophan	The fraction of the dipeptide "Asparagine-Tryptophan" in the sequence.
DW	Aspartic acid-Tryptophan	The fraction of the dipeptide "Aspartic acid-Tryptophan" in the sequence.
CW	Cysteine-Tryptophan	The fraction of the dipeptide "Cysteine-Tryptophan" in the sequence.
EW	Glutamic acid-Tryptophan	The fraction of the dipeptide "Glutamic acid-Tryptophan" in the sequence.
QW	Glutamine-Tryptophan	The fraction of the dipeptide "Glutamine-Tryptophan" in the sequence.
GW	Glycine-Tryptophan	The fraction of the

		dipeptide "Glycine-Tryptophan" in the sequence.
HW	Histidine-Tryptophan	The fraction of the dipeptide "Histidine-Tryptophan" in the sequence.
IW	Isoleucine-Tryptophan	The fraction of the dipeptide "Isoleucine-Tryptophan" in the sequence.
LW	Leucine-Tryptophan	The fraction of the dipeptide "Leucine-Tryptophan" in the sequence.
KW	Lysine-Tryptophan	The fraction of the dipeptide "Lysine-Tryptophan" in the sequence.
MW	Methionine-Tryptophan	The fraction of the dipeptide "Methionine-Tryptophan" in the sequence.
FW	Phenylalanine-Tryptophan	The fraction of the dipeptide "Phenylalanine-Tryptophan" in the sequence.
PW	Proline-Tryptophan	The fraction of the dipeptide "Proline-Tryptophan" in the sequence.
SW	Serine-Tryptophan	The fraction of the dipeptide "Serine-Tryptophan" in the sequence.
TW	Threonine-Tryptophan	The fraction of the dipeptide "Threonine-Tryptophan" in the sequence.
WW	Tryptophan-Tryptophan	The fraction of the dipeptide "Tryptophan-Tryptophan" in the sequence.
YW	Tyrosine-Tryptophan	The fraction of the

		dipeptide "Tyrosine-Tryptophan" in the sequence.
VW	Valine-Tryptophan	The fraction of the dipeptide "Valine-Tryptophan" in the sequence.
AY	Alanine-Tyrosine	The fraction of the dipeptide "Alanine-Tyrosine" in the sequence.
RY	Arginine-Tyrosine	The fraction of the dipeptide "Arginine-Tyrosine" in the sequence.
NY	Asparagine-Tyrosine	The fraction of the dipeptide "Asparagine-Tyrosine" in the sequence.
DY	Aspartic acid-Tyrosine	The fraction of the dipeptide "Aspartic acid-Tyrosine" in the sequence.
CY	Cysteine-Tyrosine	The fraction of the dipeptide "Cysteine-Tyrosine" in the sequence.
EY	Glutamic acid-Tyrosine	The fraction of the dipeptide "Glutamic acid-Tyrosine" in the sequence.
QY	Glutamine-Tyrosine	The fraction of the dipeptide "Glutamine-Tyrosine" in the sequence.
GY	Glycine-Tyrosine	The fraction of the dipeptide "Glycine-Tyrosine" in the sequence.
HY	Histidine-Tyrosine	The fraction of the dipeptide "Histidine-Tyrosine" in the sequence.
IY	Isoleucine-Tyrosine	The fraction of the

		dipeptide "Isoleucine-Tyrosine" in the sequence.
LY	Leucine-Tyrosine	The fraction of the dipeptide "Leucine-Tyrosine" in the sequence.
KY	Lysine-Tyrosine	The fraction of the dipeptide "Lysine-Tyrosine" in the sequence.
MY	Methionine-Tyrosine	The fraction of the dipeptide "Methionine-Tyrosine" in the sequence.
FY	Phenylalanine-Tyrosine	The fraction of the dipeptide "Phenylalanine-Tyrosine" in the sequence.
PY	Proline-Tyrosine	The fraction of the dipeptide "Proline-Tyrosine" in the sequence.
SY	Serine-Tyrosine	The fraction of the dipeptide "Serine-Tyrosine" in the sequence.
TY	Threonine-Tyrosine	The fraction of the dipeptide "Threonine-Tyrosine" in the sequence.
WY	Tryptophan-Tyrosine	The fraction of the dipeptide "Tryptophan-Tyrosine" in the sequence.
YY	Tyrosine-Tyrosine	The fraction of the dipeptide "Tyrosine-Tyrosine" in the sequence.
VY	Valine-Tyrosine	The fraction of the dipeptide "Valine-Tyrosine" in the sequence.
AV	Alanine-Valine	The fraction of the

		dipeptide "Alanine-Valine" in the sequence.
RV	Arginine-Valine	The fraction of the dipeptide "Arginine-Valine" in the sequence.
NV	Asparagine-Valine	The fraction of the dipeptide "Asparagine-Valine" in the sequence.
DV	Aspartic acid-Valine	The fraction of the dipeptide "Aspartic acid-Valine" in the sequence.
CV	Cysteine-Valine	The fraction of the dipeptide "Cysteine-Valine" in the sequence.
EV	Glutamic acid-Valine	The fraction of the dipeptide "Glutamic acid-Valine" in the sequence.
QV	Glutamine-Valine	The fraction of the dipeptide "Glutamine-Valine" in the sequence.
GV	Glycine-Valine	The fraction of the dipeptide "Glycine-Valine" in the sequence.
HV	Histidine-Valine	The fraction of the dipeptide "Histidine-Valine" in the sequence.
IV	Isoleucine-Valine	The fraction of the dipeptide "Isoleucine-Valine" in the sequence.
LV	Leucine-Valine	The fraction of the dipeptide "Leucine-Valine" in the sequence.
KV	Lysine-Valine	The fraction of the

		dipeptide "Lysine-Valine" in the sequence.
MV	Methionine-Valine	The fraction of the dipeptide "Methionine-Valine" in the sequence.
FV	Phenylalanine-Valine	The fraction of the dipeptide "Phenylalanine-Valine" in the sequence.
PV	Proline-Valine	The fraction of the dipeptide "Proline-Valine" in the sequence.
SV	Serine-Valine	The fraction of the dipeptide "Serine-Valine" in the sequence.
TV	Threonine-Valine	The fraction of the dipeptide "Threonine-Valine" in the sequence.
WV	Tryptophan-Valine	The fraction of the dipeptide "Tryptophan-Valine" in the sequence.
YV	Tyrosine-Valine	The fraction of the dipeptide "Tyrosine-Valine" in the sequence.
VV	Valine-Valine	The fraction of the dipeptide "Valine-Valine" in the sequence.
	Tyrosine-Tyrosine	The fraction of the dipeptide "Tyrosine-Tyrosine" in the sequence.
	Valine-Tyrosine	The fraction of the dipeptide "Valine-Tyrosine" in the sequence.
AV	Alanine-Valine	The fraction of the dipeptide "Alanine-Valine" in the sequence.
RV	Arginine-Valine	The fraction of the

		dipeptide "Arginine-Valine" in the sequence.
NV	Asparagine-Valine	The fraction of the dipeptide "Asparagine-Valine" in the sequence.
DV	Aspartic acid-Valine	The fraction of the dipeptide "Aspartic acid-Valine" in the sequence.
CV	Cysteine-Valine	The fraction of the dipeptide "Cysteine-Valine" in the sequence.
EV	Glutamic acid-Valine	The fraction of the dipeptide "Glutamic acid-Valine" in the sequence.
QV	Glutamine-Valine	The fraction of the dipeptide "Glutamine-Valine" in the sequence.
GV	Glycine-Valine	The fraction of the dipeptide "Glycine-Valine" in the sequence.
HV	Histidine-Valine	The fraction of the dipeptide "Histidine-Valine" in the sequence.
IV	Isoleucine-Valine	The fraction of the dipeptide "Isoleucine-Valine" in the sequence.
LV	Leucine-Valine	The fraction of the dipeptide "Leucine-Valine" in the sequence.
KV	Lysine-Valine	The fraction of the dipeptide "Lysine-Valine" in the sequence.
MV	Methionine-Valine	The fraction of the dipeptide

		"Methionine-Valine" in the sequence.
FV	Phenylalanine-Valine	The fraction of the dipeptide "Phenylalanine-Valine" in the sequence.
PV	Proline-Valine	The fraction of the dipeptide "Proline-Valine" in the sequence.
MoreauBroto_CIDH920105.lag1	Moreau-Broto autocorrelation - CIDH920105 - lag 1	Moreau-Broto autocorrelation of normalized average hydrophobicity scales with lag 1
MoreauBroto_CIDH920105.lag2	Moreau-Broto autocorrelation - CIDH920105 - lag 2	Moreau-Broto autocorrelation of normalized average hydrophobicity scales with lag 2
MoreauBroto_CIDH920105.lag3	Moreau-Broto autocorrelation - CIDH920105 - lag 3	Moreau-Broto autocorrelation of normalized average hydrophobicity scales with lag 3
MoreauBroto_BHAR880101.lag1	Moreau-Broto autocorrelation - BHAR880101 - lag 1	Moreau-Broto autocorrelation of average flexibility indices with lag 1
MoreauBroto_BHAR880101.lag2	Moreau-Broto autocorrelation - BHAR880101 - lag 2	Moreau-Broto autocorrelation of average flexibility indices with lag 2
MoreauBroto_BHAR880101.lag3	Moreau-Broto autocorrelation - BHAR880101 - lag 3	Moreau-Broto autocorrelation of average flexibility indices with lag 3
MoreauBroto_CHAM820101.lag1	Moreau-Broto autocorrelation - CHAM820101 - lag 1	Moreau-Broto autocorrelation of polarizability parameter with lag 1
MoreauBroto_CHAM820101.lag2	Moreau-Broto autocorrelation - CHAM820101 - lag 2	Moreau-Broto autocorrelation of polarizability parameter with lag 2

MoreauBroto_CHAM820101.lag3	Moreau-Broto autocorrelation - CHAM820101 - lag 3	Moreau-Broto autocorrelation of polarizability parameter with lag 3
MoreauBroto_CHAM820102.lag1	Moreau-Broto autocorrelation - CHAM820102 - lag 1	Moreau-Broto autocorrelation of free energy of solution in water with lag 1
MoreauBroto_CHAM820102.lag2	Moreau-Broto autocorrelation - CHAM820102 - lag 2	Moreau-Broto autocorrelation of free energy of solution in water with lag 2
MoreauBroto_CHAM820102.lag3	Moreau-Broto autocorrelation - CHAM820102 - lag 3	Moreau-Broto autocorrelation of free energy of solution in water with lag 3
MoreauBroto_CHOC760101.lag1	Moreau-Broto autocorrelation - CHOC760101 - lag 1	Moreau-Broto autocorrelation of residue accessible surface area in tripeptide with lag 1
MoreauBroto_CHOC760101.lag2	Moreau-Broto autocorrelation - CHOC760101 - lag 2	Moreau-Broto autocorrelation of residue accessible surface area in tripeptide with lag 2
MoreauBroto_CHOC760101.lag3	Moreau-Broto autocorrelation - CHOC760101 - lag 3	Moreau-Broto autocorrelation of residue accessible surface area in tripeptide with lag 3
MoreauBroto_BIGC670101.lag1	Moreau-Broto autocorrelation - BIGC670101 - lag 1	Moreau-Broto autocorrelation of residue volume with lag 1
MoreauBroto_BIGC670101.lag2	Moreau-Broto autocorrelation - BIGC670101 - lag 2	Moreau-Broto autocorrelation of residue volume with lag 2
MoreauBroto_BIGC670101.lag3	Moreau-Broto autocorrelation - BIGC670101 - lag 3	Moreau-Broto autocorrelation of residue volume with lag 3
MoreauBroto_CHAM810101.lag1	Moreau-Broto autocorrelation - CHAM810101 - lag 1	Moreau-Broto autocorrelation of steric parameter with lag 1
MoreauBroto_CHAM810101.lag2	Moreau-Broto autocorrelation - CHAM810101 - lag 2	Moreau-Broto autocorrelation of steric parameter with lag 2
MoreauBroto_CHAM810101	Moreau-Broto	Moreau-Broto

.lag3	autocorrelation - CHAM810101 - lag 3	autocorrelation of steric parameter with lag 3
Moran_CIDH920105.lag1	Moran autocorrelation - CIDH920105 - lag 1	Moran autocorrelation of normalized average hydrophobicity scales with lag 1
Moran_CIDH920105.lag2	Moran autocorrelation - CIDH920105 - lag 2	Moran autocorrelation of normalized average hydrophobicity scales with lag 2
Moran_CIDH920105.lag3	Moran autocorrelation - CIDH920105 - lag 3	Moran autocorrelation of normalized average hydrophobicity scales with lag 3
Moran_BHAR880101.lag1	Moran autocorrelation - BHAR880101 - lag 1	Moran autocorrelation of average flexibility indices with lag 1
Moran_BHAR880101.lag2	Moran autocorrelation - BHAR880101 - lag 2	Moran autocorrelation of average flexibility indices with lag 2
Moran_BHAR880101.lag3	Moran autocorrelation - BHAR880101 - lag 3	Moran autocorrelation of average flexibility indices with lag 3
Moran_CHAM820101.lag1	Moran autocorrelation - CHAM820101 - lag 1	Moran autocorrelation of polarizability parameter with lag 1
Moran_CHAM820101.lag2	Moran autocorrelation - CHAM820101 - lag 2	Moran autocorrelation of polarizability parameter with lag 2
Moran_CHAM820101.lag3	Moran autocorrelation - CHAM820101 - lag 3	Moran autocorrelation of polarizability parameter with lag 3
Moran_CHAM820102.lag1	Moran autocorrelation - CHAM820102 - lag 1	Moran autocorrelation of free energy of solution in water with lag 1
Moran_CHAM820102.lag2	Moran autocorrelation - CHAM820102 - lag 2	Moran autocorrelation of free energy of solution in water with lag 2
Moran_CHAM820102.lag3	Moran autocorrelation - CHAM820102 - lag 3	Moran autocorrelation of free energy of solution in water with lag 3
Moran_CHOC760101.lag1	Moran autocorrelation - CHOC760101 - lag 1	Moran autocorrelation of residue accessible surface area in tripeptide with lag

		1
Moran_CHOC760101.lag2	Moran autocorrelation - CHOC760101 - lag 2	Moran autocorrelation of residue accessible surface area in tripeptide with lag 2
Moran_CHOC760101.lag3	Moran autocorrelation - CHOC760101 - lag 3	Moran autocorrelation of residue accessible surface area in tripeptide with lag 3
Moran_BIGC670101.lag1	Moran autocorrelation - BIGC670101 - lag 1	Moran autocorrelation of residue volume with lag 1
Moran_BIGC670101.lag2	Moran autocorrelation - BIGC670101 - lag 2	Moran autocorrelation of residue volume with lag 2
Moran_BIGC670101.lag3	Moran autocorrelation - BIGC670101 - lag 3	Moran autocorrelation of residue volume with lag 3
Moran_CHAM810101.lag1	Moran autocorrelation - CHAM810101 - lag 1	Moran autocorrelation of steric parameter with lag 1
Moran_CHAM810101.lag2	Moran autocorrelation - CHAM810101 - lag 2	Moran autocorrelation of steric parameter with lag 2
Moran_CHAM810101.lag3	Moran autocorrelation - CHAM810101 - lag 3	Moran autocorrelation of steric parameter with lag 3
Geary_CIDH920105.lag1	Geary autocorrelation - CIDH920105 - lag 1	Geary autocorrelation of normalized average hydrophobicity scales with lag 1
Geary_CIDH920105.lag2	Geary autocorrelation - CIDH920105 - lag 2	Geary autocorrelation of normalized average hydrophobicity scales with lag 2
Geary_CIDH920105.lag3	Geary autocorrelation - CIDH920105 - lag 3	Geary autocorrelation of normalized average hydrophobicity scales with lag 3
Geary_BHAR880101.lag1	Geary autocorrelation - BHAR880101 - lag 1	Geary autocorrelation of average flexibility indices with lag 1
Geary_BHAR880101.lag2	Geary autocorrelation - BHAR880101 - lag 2	Geary autocorrelation of average flexibility indices with lag 2
Geary_BHAR880101.lag3	Geary autocorrelation - BHAR880101 - lag 3	Geary autocorrelation of average flexibility indices with lag 3
Geary_CHAM820101.lag1	Geary autocorrelation - CHAM820101 - lag 1	Geary autocorrelation of polarizability parameter

		with lag 1
Geary_CHAM820101.lag2	Geary autocorrelation - CHAM820101 - lag 2	Geary autocorrelation of polarizability parameter with lag 2
Geary_CHAM820101.lag3	Geary autocorrelation - CHAM820101 - lag 3	Geary autocorrelation of polarizability parameter with lag 3
Geary_CHAM820102.lag1	Geary autocorrelation - CHAM820102 - lag 1	Geary autocorrelation of free energy of solution in water with lag 1
Geary_CHAM820102.lag2	Geary autocorrelation - CHAM820102 - lag 2	Geary autocorrelation of free energy of solution in water with lag 2
Geary_CHAM820102.lag3	Geary autocorrelation - CHAM820102 - lag 3	Geary autocorrelation of free energy of solution in water with lag 3
Geary_CHOC760101.lag1	Geary autocorrelation - CHOC760101 - lag 1	Geary autocorrelation of residue accessible surface area in tripeptide with lag 1
Geary_CHOC760101.lag2	Geary autocorrelation - CHOC760101 - lag 2	Geary autocorrelation of residue accessible surface area in tripeptide with lag 2
Geary_CHOC760101.lag3	Geary autocorrelation - CHOC760101 - lag 3	Geary autocorrelation of residue accessible surface area in tripeptide with lag 3
Geary_BIGC670101.lag1	Geary autocorrelation - BIGC670101 - lag 1	Geary autocorrelation of residue volume with lag 1
Geary_BIGC670101.lag2	Geary autocorrelation - BIGC670101 - lag 2	Geary autocorrelation of residue volume with lag 2
Geary_BIGC670101.lag3	Geary autocorrelation - BIGC670101 - lag 3	Geary autocorrelation of residue volume with lag 3
Geary_CHAM810101.lag1	Geary autocorrelation - CHAM810101 - lag 1	Geary autocorrelation of steric parameter with lag 1
Geary_CHAM810101.lag2	Geary autocorrelation - CHAM810101 - lag 2	Geary autocorrelation of steric parameter with lag 2
Geary_CHAM810101.lag3	Geary autocorrelation - CHAM810101 - lag 3	Geary autocorrelation of steric parameter with lag 3
hydrophobicity.Group1	Hydrophobicity Group 1	The fraction of polar amino acids (R, K, E, D, Q, N) within the sequence.
hydrophobicity.Group2	Hydrophobicity Group 2	The fraction of neutral

		amino acids (G, A, S, T, P, H, Y) within the sequence.
hydrophobicity.Group3	Hydrophobicity Group 3	The fraction of hydrophobic amino acids (C, L, V, I, M, F, W) within the sequence.
normwaalsvolume.Group1	Normalized van der Waals Volume Group 1	The fraction of amino acids with normalized van der Waals volume 0-2.78 (G, A, S, T, P, D, C) within the sequence.
normwaalsvolume.Group2	Normalized van der Waals Volume Group 2	The fraction of amino acids with normalized van der Waals volume 2.95-4.0 (N, V, E, Q, I, L) within the sequence.
normwaalsvolume.Group3	Normalized van der Waals Volume Group 3	The fraction of amino acids with normalized van der Waals volume 4.03-8.08 (M, H, K, F, R, Y, W) within the sequence.
polarity.Group1	Polarity Group 1	The fraction of amino acids with polarity 4.9-6.2 (L, I, F, W, C, M, V, Y) within the sequence.
polarity.Group2	Polarity Group 2	The fraction of amino acids with polarity 8.0-9.2 (P, A, T, G, S) within the sequence.
polarity.Group3	Polarity Group 3	The fraction of amino acids with polarity 10.4-13.0 (H, Q, R, K, N, E, D) within the sequence.
polarizability.Group1	Polarizability Group 1	The fraction of amino acids with polarizability 0-1.08 (G, A, S, D, T) within the sequence.
polarizability.Group2	Polarizability Group 2	The fraction of amino acids with polarizability 0.128-0.186 (C, P, N, V, E, Q, I, L) within the sequence.
polarizability.Group3	Polarizability Group 3	The fraction of amino

		acids with polarizability 0.219-0.409 (K, M, H, F, R, Y, W) within the sequence.
charge.Group1	Charge Group 1	The fraction of positively charged amino acids (K, R) within the sequence.
charge.Group2	Charge Group 2	The fraction of neutral amino acids (A, N, C, Q, G, H, I, L, M, F, P, S, T, W, Y, V) within the sequence.
charge.Group3	Charge Group 3	The fraction of negatively charged amino acids (D, E) within the sequence.
solventaccess.Group1	Solvent Accessibility Group 1	The fraction of buried amino acids (A, L, F, C, G, I, V, W) within the sequence.
solventaccess.Group2	Solvent Accessibility Group 2	The fraction of exposed amino acids (R, K, Q, E, N, D) within the sequence.
solventaccess.Group3	Solvent Accessibility Group 3	The fraction of intermediate solvent accessibility amino acids (M, S, P, T, H, Y) within the sequence.
prop1.Tr1221	Hydrophobicity Transition 1221	Transition frequency between polar (R, K, E, D, Q, N) and neutral (G, A, S, T, P, H, Y) amino acids
prop1.Tr1331	Hydrophobicity Transition 1331	Transition frequency between polar (R, K, E, D, Q, N) and hydrophobic (C, L, V, I, M, F, W) amino acids
prop1.Tr2332	Hydrophobicity Transition 2332	Transition frequency between neutral (G, A, S, T, P, H, Y) and hydrophobic (C, L, V, I, M, F, W) amino acids
prop2.Tr1221	Normalized van der Waals Volume Transition 1221	Transition frequency between small (0-2.78: G, A, S, T, P, D, C) and

		medium (2.95-4.0: N, V, E, Q, I, L) volume amino acids
prop2.Tr1331	Normalized van der Waals Volume Transition 1331	Transition frequency between small (0-2.78: G, A, S, T, P, D, C) and large (4.03-8.08: M, H, K, F, R, Y, W) volume amino acids
prop2.Tr2332	Normalized van der Waals Volume Transition 2332	Transition frequency between medium (2.95-4.0: N, V, E, Q, I, L) and large (4.03-8.08: M, H, K, F, R, Y, W) volume amino acids
prop3.Tr1221	Polarity Transition 1221	Transition frequency between low (4.9-6.2: L, I, F, W, C, M, V, Y) and medium (8.0-9.2: P, A, T, G, S) polarity amino acids
prop3.Tr1331	Polarity Transition 1331	Transition frequency between low (4.9-6.2: L, I, F, W, C, M, V, Y) and high (10.4-13.0: H, Q, R, K, N, E, D) polarity amino acids
prop3.Tr2332	Polarity Transition 2332	Transition frequency between medium (8.0-9.2: P, A, T, G, S) and high (10.4-13.0: H, Q, R, K, N, E, D) polarity amino acids
prop4.Tr1221	Polarizability Transition 1221	Transition frequency between low (0-1.08: G, A, S, D, T) and medium (0.128-0. 186: C, P, N, V, E, Q, I, L) polarizability amino acids
prop4.Tr1331	Polarizability Transition 1331	Transition frequency between low (0-1.08: G, A, S, D, T) and high (0.219-0.409: K, M, H, F, R, Y, W) polarizability amino acids
prop4.Tr2332	Polarizability Transition 2332	Transition frequency between medium (0.128-0. 186: C, P, N, V,

		E, Q, I, L) and high (0.219-0.409: K, M, H, F, R, Y, W) polarizability amino acids
prop5.Tr1221	Charge Transition 1221	Transition frequency between positive (K, R) and neutral (A, N, C, Q, G, H, I, L, M, F, P, S, T, W, Y, V) charged amino acids
prop5.Tr1331	Charge Transition 1331	Transition frequency between positive (K, R) and negative (D, E) charged amino acids
prop5.Tr2332	Charge Transition 2332	Transition frequency between neutral (A, N, C, Q, G, H, I, L, M, F, P, S, T, W, Y, V) and negative (D, E) charged amino acids
prop6.Tr1221	Secondary Structure Transition 1221	Transition frequency between helix-forming (E, A, L, M, Q, K, R, H) and strand-forming (V, I, Y, C, W, F, T) amino acids
prop6.Tr1331	Secondary Structure Transition 1331	Transition frequency between helix-forming (E, A, L, M, Q, K, R, H) and coil-forming (G, N, P, S, D) amino acids
prop6.Tr2332	Secondary Structure Transition 2332	Transition frequency between strand-forming (V, I, Y, C, W, F, T) and coil-forming (G, N, P, S, D) amino acids
prop7.Tr1221	Solvent Accessibility Transition 1221	Transition frequency between buried (A, L, F, C, G, I, V, W) and exposed (R, K, Q, E, N, D) amino acids
prop7.Tr1331	Solvent Accessibility Transition 1331	Transition frequency between buried (A, L, F, C, G, I, V, W) and intermediate (M, S, P, T, H, Y) solvent accessibility

		amino acids
prop7.Tr2332	Solvent Accessibility Transition 2332	Transition frequency between exposed (R, K, Q, E, N, D) and intermediate (M, S, P, T, H, Y) solvent accessibility amino acids
prop1.G1.residue0	Hydrophobicity Group 1 Residue 0%	Percentage of polar amino acids (R, K, E, D, Q, N) at 0% of the sequence
prop1.G1.residue25	Hydrophobicity Group 1 Residue 25%	Percentage of polar amino acids (R, K, E, D, Q, N) at 25% of the sequence
prop1.G1.residue50	Hydrophobicity Group 1 Residue 50%	Percentage of polar amino acids (R, K, E, D, Q, N) at 50% of the sequence
prop1.G1.residue75	Hydrophobicity Group 1 Residue 75%	Percentage of polar amino acids (R, K, E, D, Q, N) at 75% of the sequence
prop1.G1.residue100	Hydrophobicity Group 1 Residue 100%	Percentage of polar amino acids (R, K, E, D, Q, N) at 100% of the sequence
prop1.G2.residue0	Hydrophobicity Group 2 Residue 0%	Percentage of neutral amino acids (G, A, S, T, P, H, Y) at 0% of the sequence
prop1.G2.residue25	Hydrophobicity Group 2 Residue 25%	Percentage of neutral amino acids (G, A, S, T, P, H, Y) at 25% of the sequence
prop1.G2.residue50	Hydrophobicity Group 2 Residue 50%	Percentage of neutral amino acids (G, A, S, T, P, H, Y) at 50% of the sequence
prop1.G2.residue75	Hydrophobicity Group 2 Residue 75%	Percentage of neutral amino acids (G, A, S, T, P, H, Y) at 75% of the sequence
prop1.G2.residue100	Hydrophobicity Group 2 Residue 100%	Percentage of neutral amino acids (G, A, S, T, P, H, Y) at 100% of the sequence
prop1.G3.residue0	Hydrophobicity Group 3 Residue 0%	Percentage of hydrophobic amino acids (C, L, V, I, M,

		F, W) at 0% of the sequence
prop1.G3.residue25	Hydrophobicity Group 3 Residue 25%	Percentage of hydrophobic amino acids (C, L, V, I, M, F, W) at 25% of the sequence
prop1.G3.residue50	Hydrophobicity Group 3 Residue 50%	Percentage of hydrophobic amino acids (C, L, V, I, M, F, W) at 50% of the sequence
prop1.G3.residue75	Hydrophobicity Group 3 Residue 75%	Percentage of hydrophobic amino acids (C, L, V, I, M, F, W) at 75% of the sequence
prop1.G3.residue100	Hydrophobicity Group 3 Residue 100%	Percentage of hydrophobic amino acids (C, L, V, I, M, F, W) at 100% of the sequence
prop2.G1.residue0	Van der Waals Volume Group 1 Residue 0%	Percentage of amino acids with normalized van der Waals volume 0-2.78 (G, A, S, T, P, D, C) at 0% of the sequence
prop2.G1.residue25	Van der Waals Volume Group 1 Residue 25%	Percentage of amino acids with normalized van der Waals volume 0-2.78 (G, A, S, T, P, D, C) at 25% of the sequence
prop2.G1.residue50	Van der Waals Volume Group 1 Residue 50%	Percentage of amino acids with normalized van der Waals volume 0-2.78 (G, A, S, T, P, D, C) at 50% of the sequence
prop2.G1.residue75	Van der Waals Volume Group 1 Residue 75%	Percentage of amino acids with normalized van der Waals volume 0-2.78 (G, A, S, T, P, D, C) at 75% of the sequence
prop2.G1.residue100	Van der Waals Volume Group 1 Residue 100%	Percentage of amino acids with normalized van der Waals volume 0-2.78 (G, A, S, T, P, D, C) at 100% of the sequence
prop2.G2.residue0	Van der Waals Volume	Percentage of amino acids

	Group 2 Residue 0%	with normalized van der Waals volume 2.95-4.0 (N, V, E, Q, I, L) at 0% of the sequence
prop2.G2.residue25	Van der Waals Volume Group 2 Residue 25%	Percentage of amino acids with normalized van der Waals volume 2.95-4.0 (N, V, E, Q, I, L) at 25% of the sequence
prop2.G2.residue50	Van der Waals Volume Group 2 Residue 50%	Percentage of amino acids with normalized van der Waals volume 2.95-4.0 (N, V, E, Q, I, L) at 50% of the sequence
prop2.G2.residue75	Van der Waals Volume Group 2 Residue 75%	Percentage of amino acids with normalized van der Waals volume 2.95-4.0 (N, V, E, Q, I, L) at 75% of the sequence
prop2.G2.residue100	Van der Waals Volume Group 2 Residue 100%	Percentage of amino acids with normalized van der Waals volume 2.95-4.0 (N, V, E, Q, I, L) at 100% of the sequence
prop2.G3.residue0	Van der Waals Volume Group 3 Residue 0%	Percentage of amino acids with normalized van der Waals volume 4.03-8.08 (M, H, K, F, R, Y, W) at 0% of the sequence
prop2.G3.residue25	Van der Waals Volume Group 3 Residue 25%	Percentage of amino acids with normalized van der Waals volume 4.03-8.08 (M, H, K, F, R, Y, W) at 25% of the sequence
prop2.G3.residue50	Van der Waals Volume Group 3 Residue 50%	Percentage of amino acids with normalized van der Waals volume 4.03-8.08 (M, H, K, F, R, Y, W) at 50% of the sequence
prop2.G3.residue75	Van der Waals Volume Group 3 Residue 75%	Percentage of amino acids with normalized van der Waals volume 4.03-8.08 (M, H, K, F, R, Y, W) at 75% of the sequence

prop2.G3.residue100	Van der Waals Volume Group 3 Residue 100%	Percentage of amino acids with normalized van der Waals volume 4.03-8.08 (M, H, K, F, R, Y, W) at 100% of the sequence
prop3.G1.residue0	Polarity Group 1 Residue 0%	Percentage of amino acids with polarity 4.9-6.2 (L, I, F, W, C, M, V, Y) at 0% of the sequence
prop3.G1.residue25	Polarity Group 1 Residue 25%	Percentage of amino acids with polarity 4.9-6.2 (L, I, F, W, C, M, V, Y) at 25% of the sequence
prop3.G1.residue50	Polarity Group 1 Residue 50%	Percentage of amino acids with polarity 4.9-6.2 (L, I, F, W, C, M, V, Y) at 50% of the sequence
prop3.G1.residue75	Polarity Group 1 Residue 75%	Percentage of amino acids with polarity 4.9-6.2 (L, I, F, W, C, M, V, Y) at 75% of the sequence
prop3.G1.residue100	Polarity Group 1 Residue 100%	Percentage of amino acids with polarity 4.9-6.2 (L, I, F, W, C, M, V, Y) at 100% of the sequence
prop3.G2.residue0	Polarity Group 2 Residue 0%	Percentage of amino acids with polarity 8.0-9.2 (P, A, T, G, S) at 0% of the sequence
prop3.G2.residue25	Polarity Group 2 Residue 25%	Percentage of amino acids with polarity 8.0-9.2 (P, A, T, G, S) at 25% of the sequence
prop3.G2.residue50	Polarity Group 2 Residue 50%	Percentage of amino acids with polarity 8.0-9.2 (P, A, T, G, S) at 50% of the sequence
prop3.G2.residue75	Polarity Group 2 Residue 75%	Percentage of amino acids with polarity 8.0-9.2 (P, A, T, G, S) at 75% of the sequence
prop3.G2.residue100	Polarity Group 2 Residue 100%	Percentage of amino acids with polarity 8.0-9.2 (P, A, T, G, S) at 100% of the

		sequence
prop3.G3.residue0	Polarity Group 3 Residue 0%	Percentage of amino acids with polarity 10.4-13.0 (H, Q, R, K, N, E, D) at 0% of the sequence
prop3.G3.residue25	Polarity Group 3 Residue 25%	Percentage of amino acids with polarity 10.4-13.0 (H, Q, R, K, N, E, D) at 25% of the sequence
prop3.G3.residue50	Polarity Group 3 Residue 50%	Percentage of amino acids with polarity 10.4-13.0 (H, Q, R, K, N, E, D) at 50% of the sequence
prop3.G3.residue75	Polarity Group 3 Residue 75%	Percentage of amino acids with polarity 10.4-13.0 (H, Q, R, K, N, E, D) at 75% of the sequence
prop3.G3.residue100	Polarity Group 3 Residue 100%	Percentage of amino acids with polarity 10.4-13.0 (H, Q, R, K, N, E, D) at 100% of the sequence
prop4.G1.residue0	Polarizability Group 1 Residue 0%	Percentage of amino acids with polarizability 0-1.08 (G, A, S, D, T) at 0% of the sequence
prop4.G1.residue25	Polarizability Group 1 Residue 25%	Percentage of amino acids with polarizability 0-1.08 (G, A, S, D, T) at 25% of the sequence
prop4.G1.residue50	Polarizability Group 1 Residue 50%	Percentage of amino acids with polarizability 0-1.08 (G, A, S, D, T) at 50% of the sequence
prop4.G1.residue75	Polarizability Group 1 Residue 75%	Percentage of amino acids with polarizability 0-1.08 (G, A, S, D, T) at 75% of the sequence
prop4.G1.residue100	Polarizability Group 1 Residue 100%	Percentage of amino acids with polarizability 0-1.08 (G, A, S, D, T) at 100% of the sequence
prop4.G2.residue0	Polarizability Group 2 Residue 0%	Percentage of amino acids with polarizability 0.128-0.186 (C, P, N, V, E,

		Q, I, L) at 0% of the sequence
prop4.G2.residue25	Polarizability Group 2 Residue 25%	Percentage of amino acids with polarizability 0.128-0. 186 (C, P, N, V, E, Q, I, L) at 25% of the sequence
prop4.G2.residue50	Polarizability Group 2 Residue 50%	Percentage of amino acids with polarizability 0.128-0. 186 (C, P, N, V, E, Q, I, L) at 50% of the sequence
prop4.G2.residue75	Polarizability Group 2 Residue 75%	Percentage of amino acids with polarizability 0.128-0. 186 (C, P, N, V, E, Q, I, L) at 75% of the sequence
prop4.G2.residue100	Polarizability Group 2 Residue 100%	Percentage of amino acids with polarizability 0.128-0. 186 (C, P, N, V, E, Q, I, L) at 100% of the sequence
prop4.G3.residue0	Polarizability Group 3 Residue 0%	Percentage of amino acids with polarizability 0.219-0.409 (K, M, H, F, R, Y, W) at 0% of the sequence
prop4.G3.residue25	Polarizability Group 3 Residue 25%	Percentage of amino acids with polarizability 0.219-0.409 (K, M, H, F, R, Y, W) at 25% of the sequence
prop4.G3.residue50	Polarizability Group 3 Residue 50%	Percentage of amino acids with polarizability 0.219-0.409 (K, M, H, F, R, Y, W) at 50% of the sequence
prop4.G3.residue75	Polarizability Group 3 Residue 75%	Percentage of amino acids with polarizability 0.219-0.409 (K, M, H, F, R, Y, W) at 75% of the sequence
prop4.G3.residue100	Polarizability Group 3 Residue 100%	Percentage of amino acids with polarizability

		0.219-0.409 (K, M, H, F, R, Y, W) at 100% of the sequence
prop5.G1.residue0	Charge Group 1 Residue 0%	Percentage of positively charged amino acids (K, R) at 0% of the sequence
prop5.G1.residue25	Charge Group 1 Residue 25%	Percentage of positively charged amino acids (K, R) at 25% of the sequence
prop5.G1.residue50	Charge Group 1 Residue 50%	Percentage of positively charged amino acids (K, R) at 50% of the sequence
prop5.G1.residue75	Charge Group 1 Residue 75%	Percentage of positively charged amino acids (K, R) at 75% of the sequence
prop5.G1.residue100	Charge Group 1 Residue 100%	Percentage of positively charged amino acids (K, R) at 100% of the sequence
prop5.G2.residue0	Charge Group 2 Residue 0%	Percentage of neutral amino acids (A, N, C, Q, G, H, I, L, M, F, P, S, T, W, Y, V) at 0% of the sequence
prop5.G2.residue25	Charge Group 2 Residue 25%	Percentage of neutral amino acids (A, N, C, Q, G, H, I, L, M, F, P, S, T, W, Y, V) at 25% of the sequence
prop5.G2.residue50	Charge Group 2 Residue 50%	Percentage of neutral amino acids (A, N, C, Q, G, H, I, L, M, F, P, S, T, W, Y, V) at 50% of the sequence
prop5.G2.residue75	Charge Group 2 Residue 75%	Percentage of neutral amino acids (A, N, C, Q, G, H, I, L, M, F, P, S, T, W, Y, V) at 75% of the sequence
prop5.G2.residue100	Charge Group 2 Residue 100%	Percentage of neutral amino acids (A, N, C, Q, G, H, I, L, M, F, P, S, T, W, Y, V) at 100% of the sequence

prop5.G3.residue0	Charge Group 3 Residue 0%	Percentage of negatively charged amino acids (D, E) at 0% of the sequence
prop5.G3.residue25	Charge Group 3 Residue 25%	Percentage of negatively charged amino acids (D, E) at 25% of the sequence
prop5.G3.residue50	Charge Group 3 Residue 50%	Percentage of negatively charged amino acids (D, E) at 50% of the sequence
prop5.G3.residue75	Charge Group 3 Residue 75%	Percentage of negatively charged amino acids (D, E) at 75% of the sequence
prop5.G3.residue100	Charge Group 3 Residue 100%	Percentage of negatively charged amino acids (D, E) at 100% of the sequence
prop7.G1.residue0	Solvent Accessibility Group 1 Residue 0%	Percentage of buried amino acids (A, L, F, C, G, I, V, W) at 0% of the sequence
prop7.G1.residue25	Solvent Accessibility Group 1 Residue 25%	Percentage of buried amino acids (A, L, F, C, G, I, V, W) at 25% of the sequence
prop7.G1.residue50	Solvent Accessibility Group 1 Residue 50%	Percentage of buried amino acids (A, L, F, C, G, I, V, W) at 50% of the sequence
prop7.G1.residue75	Solvent Accessibility Group 1 Residue 75%	Percentage of buried amino acids (A, L, F, C, G, I, V, W) at 75% of the sequence
prop7.G1.residue100	Solvent Accessibility Group 1 Residue 100%	Percentage of buried amino acids (A, L, F, C, G, I, V, W) at 100% of the sequence
prop7.G2.residue0	Solvent Accessibility Group 2 Residue 0%	Percentage of exposed amino acids (R, K, Q, E, N, D) at 0% of the sequence
prop7.G2.residue25	Solvent Accessibility Group 2 Residue 25%	Percentage of exposed amino acids (R, K, Q, E, N, D) at 25% of the sequence

prop7.G2.residue50	Solvent Accessibility Group 2 Residue 50%	Percentage of exposed amino acids (R, K, Q, E, N, D) at 50% of the sequence
prop7.G2.residue75	Solvent Accessibility Group 2 Residue 75%	Percentage of exposed amino acids (R, K, Q, E, N, D) at 75% of the sequence
prop7.G2.residue100	Solvent Accessibility Group 2 Residue 100%	Percentage of exposed amino acids (R, K, Q, E, N, D) at 100% of the sequence
prop7.G3.residue0	Solvent Accessibility Group 3 Residue 0%	Percentage of intermediate amino acids (M, S, P, T, H, Y) at 0% of the sequence
prop7.G3.residue25	Solvent Accessibility Group 3 Residue 25%	Percentage of intermediate amino acids (M, S, P, T, H, Y) at 25% of the sequence
prop7.G3.residue50	Solvent Accessibility Group 3 Residue 50%	Percentage of intermediate amino acids (M, S, P, T, H, Y) at 50% of the sequence
prop7.G3.residue75	Solvent Accessibility Group 3 Residue 75%	Percentage of intermediate amino acids (M, S, P, T, H, Y) at 75% of the sequence
prop7.G3.residue100	Solvent Accessibility Group 3 Residue 100%	Percentage of intermediate amino acids (M, S, P, T, H, Y) at 100% of the sequence
Schneider.lag1	Schneider Sequence-Order-Coupling Number (Lag 1)	The first-rank sequence-order-coupling number based on Schneider's distance matrix between amino acids
Schneider.lag2	Schneider Sequence-Order-Coupling Number (Lag 2)	The second-rank sequence-order-coupling number based on Schneider's distance matrix between amino acids
Schneider.lag3	Schneider Sequence-Order-Coupling Number (Lag 3)	The third-rank sequence-order-coupling number based on Schneider's distance

		matrix between amino acids
Grantham.lag1	Grantham Sequence-Order-Coupling Number (Lag 1)	The first-rank sequence-order-coupling number based on Grantham's distance matrix between amino acids
Grantham.lag2	Grantham Sequence-Order-Coupling Number (Lag 2)	The second-rank sequence-order-coupling number based on Grantham's distance matrix between amino acids
Grantham.lag3	Grantham Sequence-Order-Coupling Number (Lag 3)	The third-rank sequence-order-coupling number based on Grantham's distance matrix between amino acids
Schneider.Xr.A	Schneider Quasi-Sequence-Order for Alanine	Quasi-sequence-order descriptor for Alanine based on Schneider's matrix
Schneider.Xr.R	Schneider Quasi-Sequence-Order for Arginine	Quasi-sequence-order descriptor for Arginine based on Schneider's matrix
Schneider.Xr.N	Schneider Quasi-Sequence-Order for Asparagine	Quasi-sequence-order descriptor for Asparagine based on Schneider's matrix
Schneider.Xr.D	Schneider Quasi-Sequence-Order for Aspartic Acid	Quasi-sequence-order descriptor for Aspartic Acid based on Schneider's matrix
Schneider.Xr.C	Schneider Quasi-Sequence-Order for Cysteine	Quasi-sequence-order descriptor for Cysteine based on Schneider's matrix
Schneider.Xr.E	Schneider Quasi-Sequence-Order for Glutamic Acid	Quasi-sequence-order descriptor for Glutamic Acid based on Schneider's matrix

Schneider.Xr.Q	Schneider Quasi-Sequence-Order for Glutamine	Quasi-sequence-order descriptor for Glutamine based on Schneider's matrix
Schneider.Xr.G	Schneider Quasi-Sequence-Order for Glycine	Quasi-sequence-order descriptor for Glycine based on Schneider's matrix
Schneider.Xr.H	Schneider Quasi-Sequence-Order for Histidine	Quasi-sequence-order descriptor for Histidine based on Schneider's matrix
Schneider.Xr.I	Schneider Quasi-Sequence-Order for Isoleucine	Quasi-sequence-order descriptor for Isoleucine based on Schneider's matrix
Schneider.Xr.L	Schneider Quasi-Sequence-Order for Leucine	Quasi-sequence-order descriptor for Leucine based on Schneider's matrix
Schneider.Xr.K	Schneider Quasi-Sequence-Order for Lysine	Quasi-sequence-order descriptor for Lysine based on Schneider's matrix
Schneider.Xr.M	Schneider Quasi-Sequence-Order for Methionine	Quasi-sequence-order descriptor for Methionine based on Schneider's matrix
Schneider.Xr.F	Schneider Quasi-Sequence-Order for Phenylalanine	Quasi-sequence-order descriptor for Phenylalanine based on Schneider's matrix
Schneider.Xr.P	Schneider Quasi-Sequence-Order for Proline	Quasi-sequence-order descriptor for Proline based on Schneider's matrix
Schneider.Xr.S	Schneider Quasi-Sequence-Order for Serine	Quasi-sequence-order descriptor for Serine based on Schneider's matrix
Schneider.Xr.T	Schneider Quasi-Sequence-Order for Threonine	Quasi-sequence-order descriptor for Threonine based on Schneider's matrix
Schneider.Xr.W	Schneider	Quasi-sequence-order

	Quasi-Sequence-Order for Tryptophan	descriptor for Tryptophan based on Schneider's matrix
Schneider.Xr.Y	Schneider Quasi-Sequence-Order for Tyrosine	Quasi-sequence-order descriptor for Tyrosine based on Schneider's matrix
Schneider.Xr.V	Schneider Quasi-Sequence-Order for Valine	Quasi-sequence-order descriptor for Valine based on Schneider's matrix
Grantham.Xr.A	Grantham Quasi-Sequence-Order for Alanine	Quasi-sequence-order descriptor for Alanine based on Grantham's matrix
Grantham.Xr.R	Grantham Quasi-Sequence-Order for Arginine	Quasi-sequence-order descriptor for Arginine based on Grantham's matrix
Grantham.Xr.N	Grantham Quasi-Sequence-Order for Asparagine	Quasi-sequence-order descriptor for Asparagine based on Grantham's matrix
Grantham.Xr.D	Grantham Quasi-Sequence-Order for Aspartic Acid	Quasi-sequence-order descriptor for Aspartic Acid based on Grantham's matrix
Grantham.Xr.C	Grantham Quasi-Sequence-Order for Cysteine	Quasi-sequence-order descriptor for Cysteine based on Grantham's matrix
Grantham.Xr.E	Grantham Quasi-Sequence-Order for Glutamic Acid	Quasi-sequence-order descriptor for Glutamic Acid based on Grantham's matrix
Grantham.Xr.Q	Grantham Quasi-Sequence-Order for Glutamine	Quasi-sequence-order descriptor for Glutamine based on Grantham's matrix
Grantham.Xr.G	Grantham Quasi-Sequence-Order for Glycine	Quasi-sequence-order descriptor for Glycine based on Grantham's matrix
Grantham.Xr.H	Grantham Quasi-Sequence-Order	Quasi-sequence-order descriptor for Histidine

	for Histidine	based on Grantham's matrix
Grantham.Xr.I	Grantham Quasi-Sequence-Order for Isoleucine	Quasi-sequence-order descriptor for Isoleucine based on Grantham's matrix
Grantham.Xr.L	Grantham Quasi-Sequence-Order for Leucine	Quasi-sequence-order descriptor for Leucine based on Grantham's matrix
Grantham.Xr.K	Grantham Quasi-Sequence-Order for Lysine	Quasi-sequence-order descriptor for Lysine based on Grantham's matrix
Grantham.Xr.M	Grantham Quasi-Sequence-Order for Methionine	Quasi-sequence-order descriptor for Methionine based on Grantham's matrix
Grantham.Xr.F	Grantham Quasi-Sequence-Order for Phenylalanine	Quasi-sequence-order descriptor for Phenylalanine based on Grantham's matrix
Grantham.Xr.P	Grantham Quasi-Sequence-Order for Proline	Quasi-sequence-order descriptor for Proline based on Grantham's matrix
Grantham.Xr.S	Grantham Quasi-Sequence-Order for Serine	Quasi-sequence-order descriptor for Serine based on Grantham's matrix
Grantham.Xr.T	Grantham Quasi-Sequence-Order for Threonine	Quasi-sequence-order descriptor for Threonine based on Grantham's matrix
Grantham.Xr.W	Grantham Quasi-Sequence-Order for Tryptophan	Quasi-sequence-order descriptor for Tryptophan based on Grantham's matrix
Grantham.Xr.Y	Grantham Quasi-Sequence-Order for Tyrosine	Quasi-sequence-order descriptor for Tyrosine based on Grantham's matrix
Grantham.Xr.V	Grantham Quasi-Sequence-Order for Valine	Quasi-sequence-order descriptor for Valine based on Grantham's matrix

Schneider.Xd.1	Schneider 1st-rank Quasi-Sequence-Order	Quasi-sequence-order descriptor for 1st rank sequence-order-coupling, based on Schneider-Wrede matrix.
Schneider.Xd.2	Schneider 2nd-rank Quasi-Sequence-Order	Quasi-sequence-order descriptor for 2nd rank sequence-order-coupling, based on Schneider-Wrede matrix.
Schneider.Xd.3	Schneider 3rd-rank Quasi-Sequence-Order	Quasi-sequence-order descriptor for 3rd rank sequence-order-coupling, based on Schneider-Wrede matrix.
Grantham.Xd.1	Grantham 1st-rank Quasi-Sequence-Order	Quasi-sequence-order descriptor for 1st rank sequence-order-coupling, based on Grantham matrix.
Grantham.Xd.2	Grantham 2nd-rank Quasi-Sequence-Order	Quasi-sequence-order descriptor for 2nd rank sequence-order-coupling, based on Grantham matrix.
Grantham.Xd.3	Grantham 3rd-rank Quasi-Sequence-Order	Quasi-sequence-order descriptor for 3rd rank sequence-order-coupling, based on Grantham matrix.
Xc1.A	PseAAC for Alanine	Pseudo-amino acid composition for alanine based on hydrophobicity, hydrophilicity, and side chain mass
Xc1.R	PseAAC for Arginine	Pseudo-amino acid composition for arginine based on hydrophobicity, hydrophilicity, and side chain mass
Xc1.N	PseAAC for Asparagine	Pseudo-amino acid composition for asparagine based on hydrophobicity,

		hydrophilicity, and side chain mass
Xc1.D	PseAAC for Aspartic Acid	Pseudo-amino acid composition for aspartic acid based on hydrophobicity, hydrophilicity, and side chain mass
Xc1.C	PseAAC for Cysteine	Pseudo-amino acid composition for cysteine based on hydrophobicity, hydrophilicity, and side chain mass
Xc1.E	PseAAC for Glutamic Acid	Pseudo-amino acid composition for glutamic acid based on hydrophobicity, hydrophilicity, and side chain mass
Xc1.Q	PseAAC for Glutamine	Pseudo-amino acid composition for glutamine based on hydrophobicity, hydrophilicity, and side chain mass
Xc1.G	PseAAC for Glycine	Pseudo-amino acid composition for glycine based on hydrophobicity, hydrophilicity, and side chain mass
Xc1.H	PseAAC for Histidine	Pseudo-amino acid composition for histidine based on hydrophobicity, hydrophilicity, and side chain mass
Xc1.I	PseAAC for Isoleucine	Pseudo-amino acid composition for isoleucine based on hydrophobicity, hydrophilicity, and side chain mass
Xc1.L	PseAAC for Leucine	Pseudo-amino acid composition for leucine based on hydrophobicity, hydrophilicity, and side chain mass

Xc1.K	PseAAC for Lysine	Pseudo-amino acid composition for lysine based on hydrophobicity, hydrophilicity, and side chain mass
Xc1.M	PseAAC for Methionine	Pseudo-amino acid composition for methionine based on hydrophobicity, hydrophilicity, and side chain mass
Xc1.F	PseAAC for Phenylalanine	Pseudo-amino acid composition for phenylalanine based on hydrophobicity, hydrophilicity, and side chain mass
Xc1.P	PseAAC for Proline	Pseudo-amino acid composition for proline based on hydrophobicity, hydrophilicity, and side chain mass
Xc1.S	PseAAC for Serine	Pseudo-amino acid composition for serine based on hydrophobicity, hydrophilicity, and side chain mass
Xc1.T	PseAAC for Threonine	Pseudo-amino acid composition for threonine based on hydrophobicity, hydrophilicity, and side chain mass
Xc1.W	PseAAC for Tryptophan	Pseudo-amino acid composition for tryptophan based on hydrophobicity, hydrophilicity, and side chain mass
Xc1.Y	PseAAC for Tyrosine	Pseudo-amino acid composition for tyrosine based on hydrophobicity, hydrophilicity, and side chain mass
Xc1.V	PseAAC for Valine	Pseudo-amino acid

		composition for valine based on hydrophobicity, hydrophilicity, and side chain mass
Xc2.lambda.1	1st-tier Sequence Coupling	Pseudo-amino acid composition for 1st-rank sequence-order-coupling based on hydrophobicity, hydrophilicity, and side chain mass
Xc2.lambda.2	2nd-tier Sequence Coupling	Pseudo-amino acid composition for 2nd-rank sequence-order-coupling based on hydrophobicity, hydrophilicity, and side chain mass
Xc2.lambda.3	3rd-tier Sequence Coupling	Pseudo-amino acid composition for 3rd-rank sequence-order-coupling based on hydrophobicity, hydrophilicity, and side chain mass
Pc1.A	Amphiphilic pseudo-amino acid composition for alanine	Reflects the contribution of alanine to the sequence, weighted by hydrophobicity and hydrophilicity correlations.
Pc1.R	Amphiphilic pseudo-amino acid composition for arginine	Reflects the contribution of arginine to the sequence, weighted by hydrophobicity and hydrophilicity correlations.
Pc1.N	Amphiphilic pseudo-amino acid composition for asparagine	Reflects the contribution of asparagine to the sequence, weighted by hydrophobicity and hydrophilicity correlations.
Pc1.D	Amphiphilic pseudo-amino acid composition for aspartic acid	Reflects the contribution of aspartic acid to the sequence, weighted by hydrophobicity and

		hydrophilicity correlations.
Pc1.C	Amphiphilic pseudo-amino acid composition for cysteine	Reflects the contribution of cysteine to the sequence, weighted by hydrophobicity and hydrophilicity correlations.
Pc1.E	Amphiphilic pseudo-amino acid composition for glutamic acid	Reflects the contribution of glutamic acid to the sequence, weighted by hydrophobicity and hydrophilicity correlations.
Pc1.Q	Amphiphilic pseudo-amino acid composition for glutamine	Reflects the contribution of glutamine to the sequence, weighted by hydrophobicity and hydrophilicity correlations.
Pc1.G	Amphiphilic pseudo-amino acid composition for glycine	Reflects the contribution of glycine to the sequence, weighted by hydrophobicity and hydrophilicity correlations.
Pc1.H	Amphiphilic pseudo-amino acid composition for histidine	Reflects the contribution of histidine to the sequence, weighted by hydrophobicity and hydrophilicity correlations.
Pc1.I	Amphiphilic pseudo-amino acid composition for isoleucine	Reflects the contribution of isoleucine to the sequence, weighted by hydrophobicity and hydrophilicity correlations.
Pc1.L	Amphiphilic pseudo-amino acid composition for leucine	Reflects the contribution of leucine to the sequence, weighted by hydrophobicity and hydrophilicity correlations.

Pc1.K	Amphiphilic pseudo-amino acid composition for lysine	Reflects the contribution of lysine to the sequence, weighted by hydrophobicity and hydrophilicity correlations.
Pc1.M	Amphiphilic pseudo-amino acid composition for methionine	Reflects the contribution of methionine to the sequence, weighted by hydrophobicity and hydrophilicity correlations.
Pc1.F	Amphiphilic pseudo-amino acid composition for phenylalanine	Reflects the contribution of phenylalanine to the sequence, weighted by hydrophobicity and hydrophilicity correlations.
Pc1.P	Amphiphilic pseudo-amino acid composition for proline	Reflects the contribution of proline to the sequence, weighted by hydrophobicity and hydrophilicity correlations.
Pc1.S	Amphiphilic pseudo-amino acid composition for serine	Reflects the contribution of serine to the sequence, weighted by hydrophobicity and hydrophilicity correlations.
Pc1.T	Amphiphilic pseudo-amino acid composition for threonine	Reflects the contribution of threonine to the sequence, weighted by hydrophobicity and hydrophilicity correlations.
Pc1.W	Amphiphilic pseudo-amino acid composition for tryptophan	Reflects the contribution of tryptophan to the sequence, weighted by hydrophobicity and hydrophilicity correlations.
Pc1.Y	Amphiphilic pseudo-amino acid	Reflects the contribution of tyrosine to the

	composition for tyrosine	sequence, weighted by hydrophobicity and hydrophilicity correlations.
Pc1.V	Amphiphilic pseudo-amino acid composition for valine	Reflects the contribution of valine to the sequence, weighted by hydrophobicity and hydrophilicity correlations.
Pc2.Hydrophobicity.1	1st-rank sequence-order coupling factor for hydrophobicity	Reflects the correlation between adjacent residues based on hydrophobicity.
Pc2.Hydrophilicity.1	1st-rank sequence-order coupling factor for hydrophilicity	Reflects the correlation between adjacent residues based on hydrophilicity.
Pc2.Hydrophobicity.2	2nd-rank sequence-order coupling factor for hydrophobicity	Reflects the correlation between residues separated by one position based on hydrophobicity.
Pc2.Hydrophilicity.2	2nd-rank sequence-order coupling factor for hydrophilicity	Reflects the correlation between residues separated by one position based on hydrophilicity.
Pc2.Hydrophobicity.3	3rd-rank sequence-order coupling factor for hydrophobicity	Reflects the correlation between residues separated by two positions based on hydrophobicity.
Pc2.Hydrophilicity.3	3rd-rank sequence-order coupling factor for hydrophilicity	Reflects the correlation between residues separated by two positions based on hydrophilicity.
net.charge	Net Charge	This variable represents the theoretical net charge of a peptide sequence, which is calculated using the Henderson-Hasselbalch equation at a defined pH.
hydrophobicity.value	Hydrophobicity Index	This variable represents the GRAVY (Grand Average of Hydropathy) hydrophobicity index of an amino acids sequence,

		computed using one of the 38 scales from different sources.
boman.index	Boman Index	This variable represents the potential peptide interaction index proposed by Boman, which estimates the potential of a peptide to bind to membranes or other proteins.
tiny	Tiny	This variable represents the composition of tiny amino acids (A + C + G + S + T) in a peptide sequence.
small	Small	This variable represents the composition of small amino acids (A + B + C + D + G + N + P + S + T + V) in a peptide sequence.
aliphatic	Aliphatic	This variable represents the composition of aliphatic amino acids (A + I + L + V) in a peptide sequence.
aromatic	Aromatic	This variable represents the composition of aromatic amino acids (F + H + W + Y) in a peptide sequence.
nonpolar	Non-polar	This variable represents the composition of non-polar amino acids (A + C + F + G + I + L + M + P + V + W + Y) in a peptide sequence.
polar	Polar	This variable represents the composition of polar amino acids (D + E + H + K + N + Q + R + S + T + Z) in a peptide sequence.
charged	Charged	This variable represents the composition of

		charged amino acids (B + D + E + H + K + R + Z) in a peptide sequence.
basic	Basic	This variable represents the composition of basic amino acids (H + K + R) in a peptide sequence.
acidic	Acidic	This variable represents the composition of acidic amino acids (B + D + E + Z) in a peptide sequence.
molecular_weight	Molecular weight	This variable represents the molecular weight of a peptide sequence.
