# Supplementary material for: Uniform design-embedded predictions of (tetra-)peptide physicochemical properties
Source: Bioinformatics. 2026 Jan 19;42(3):btag036. doi: 10.1093/bioinformatics/btag036 (PMC13032896; doi:10.1093/bioinformatics/btag036)
Supplement: btag036_Supplementary_Data [file btag036_supplementary_data.zip › Supplementary Information.pdf]

Supporting Information for

Uniform Design-Embedded Predictions of (Tetra-)Peptide Physicochemical Properties

Zhihui Zhu[2], Huapeng Liu[3], Xuechen Li[1], Haojin Zhou[3]*, Jiaqi Wang[1]*

*: Corresponding authors:

Haojin.Zhou@xjtlu.edu.cn; benwang@hku.hk

1. Department of Chemistry, State Key Laboratory of Synthetic Chemistry, The University of Hong Kong, Pokfulam 999077, Hong Kong SAR, China.
2. ZJU-Hangzhou Global Scientific and Technological Innovation Center, Zhejiang University, Hangzhou 311215, Zhejiang, China.
3. Wisdom Lake Academy of Pharmacy, Xi'an Jiangtong-Liverpool University, Suzhou 215123, Jiangsu, China.

Section 1: Supplementary Figures

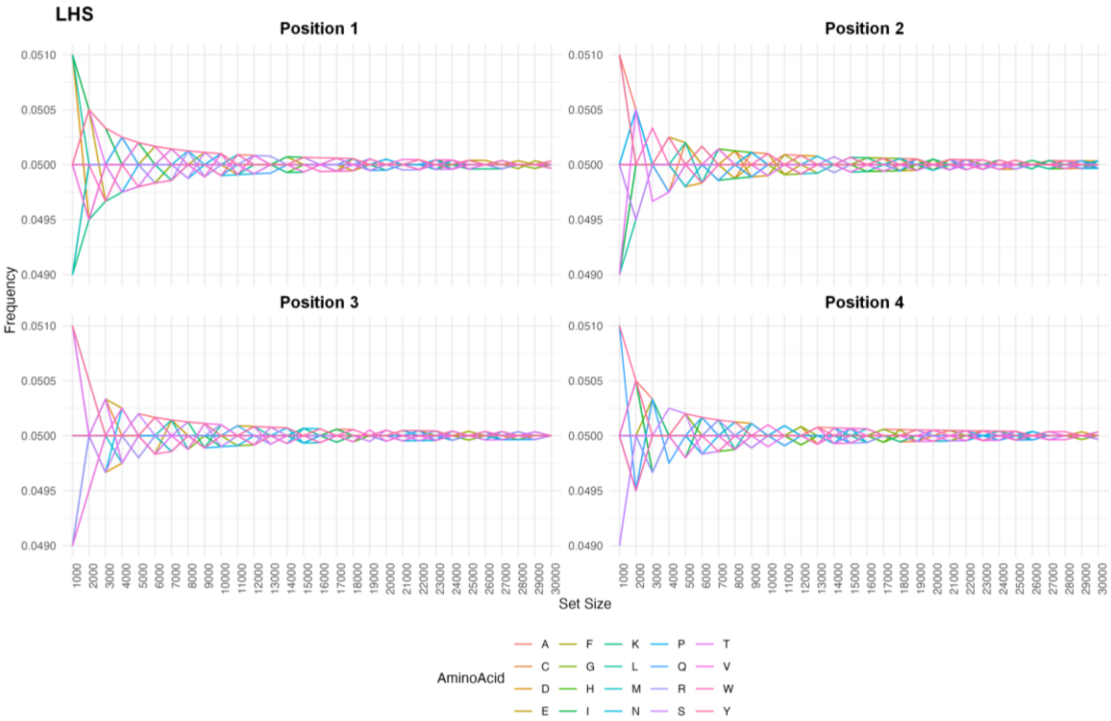

Figure S1. Marginal amino acid frequencies per position for peptide sequences sampled using Latin Hypercube Sampling (LHS).

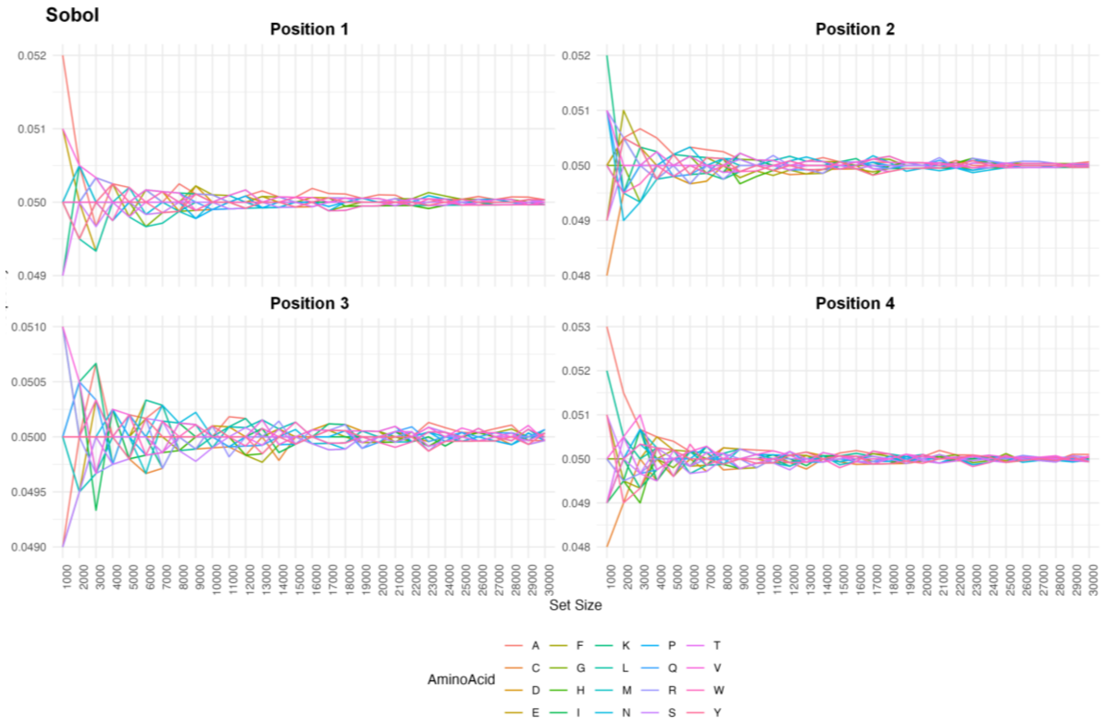

Figure S2. Marginal amino acid frequencies per position for peptide sequences sampled using Sobol sequences.

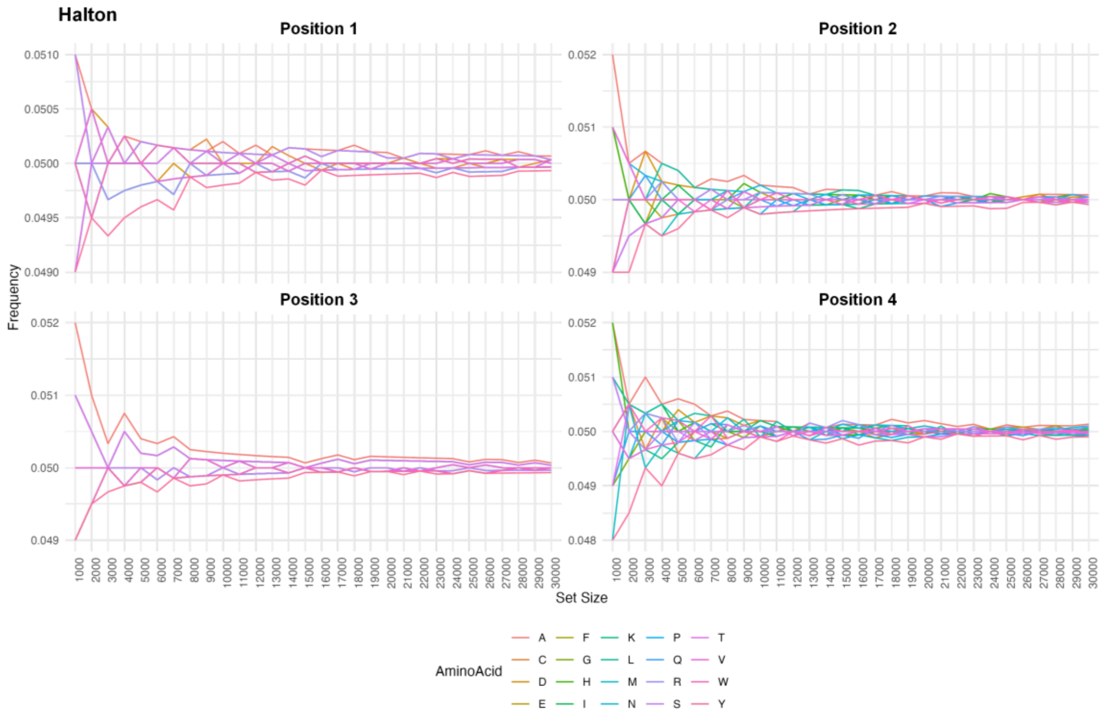

Figure S3. Marginal amino acid frequencies per position for peptide sequences sampled using Halton sequences.

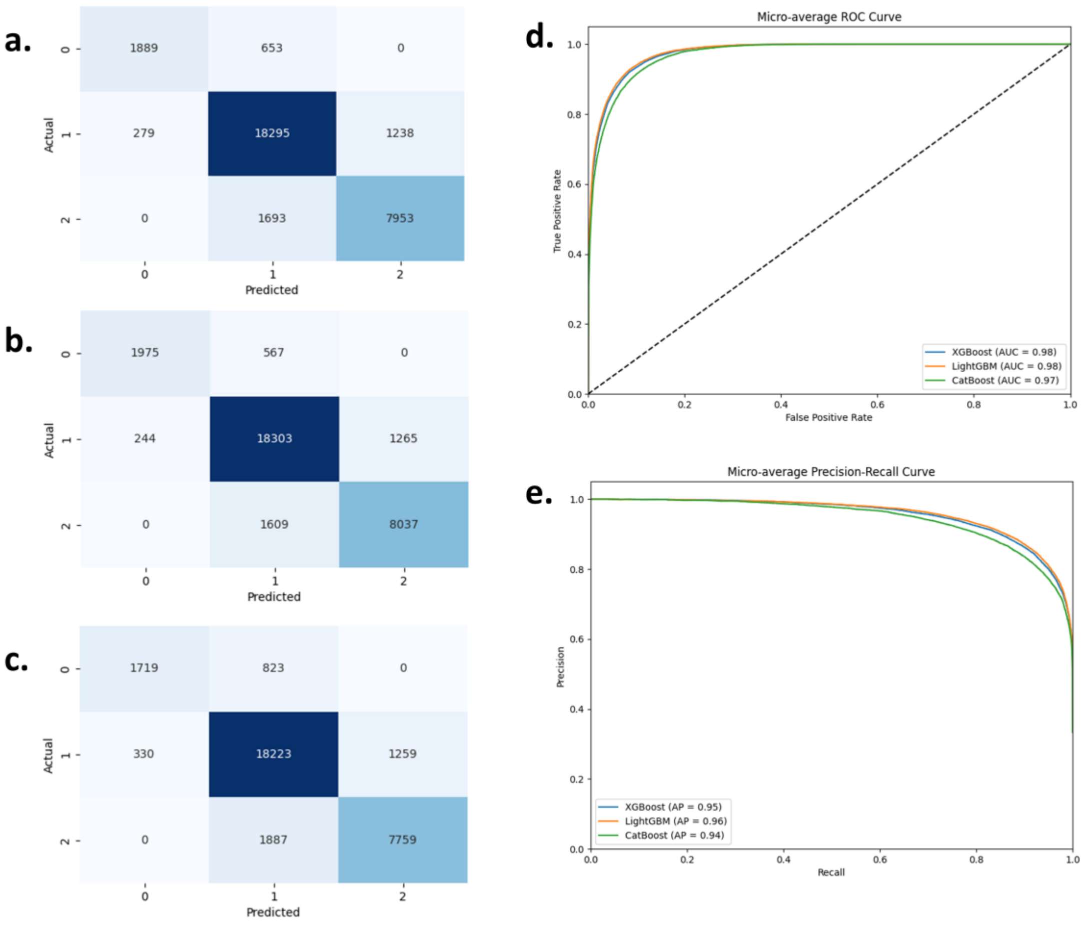

Figure S4. Performance Evaluation of Different tree Models for Multiclass classification. **a)** Confusion Matrix for LightGBM. It shows the performance of LightGBM in classifying three classes (0, 1, 2). The matrix indicates the number of true positive, false negative, and false positive predictions for each class. **b)** Confusion Matrix for XGBoost displaying the classification performance across the three classes. **c)** Confusion Matrix for CatBoost, presenting its classification performance for the three classes. **d)** Micro-average ROC Curve for all models, comparing their diagnostic ability. The Area Under the Curve (AUC) values of LightGBM, XGBoost, and CatBoost are 0.98, 0.98, and 0.97 respectively. **e)** Micro-average Precision-Recall Curve for all models. The values are given by LightGBM (0.96), XGBoost (0.95), and CatBoost (0.94).

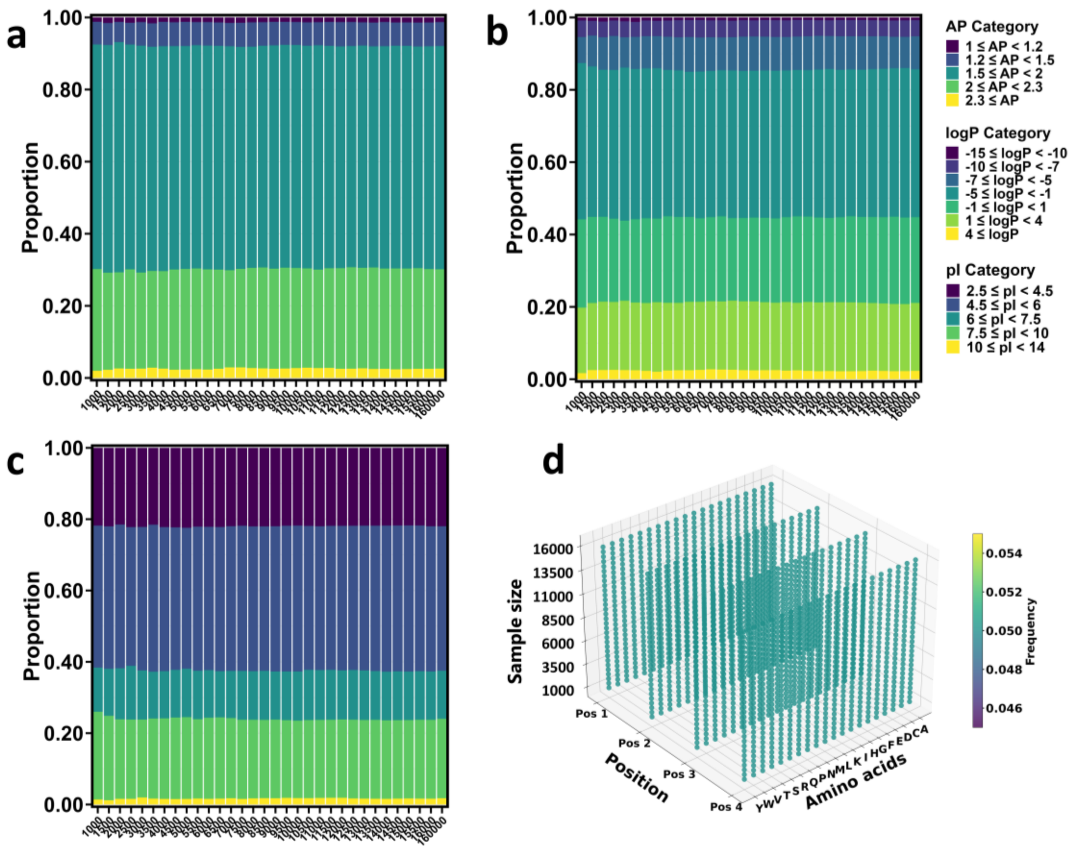

Figure S5. a-c) UD sampling of 1,000 to 16,000 samples (with each increase of 500 data) compared to the population data at different intervals of AP, logP, and pI attributes. **d)** The frequency of 20 amino acids at different positions in the tetrapeptide sequence for 1,000 to 16,000 tetrapeptide sequence data (increasing 500 at a time) under different sample numbers.

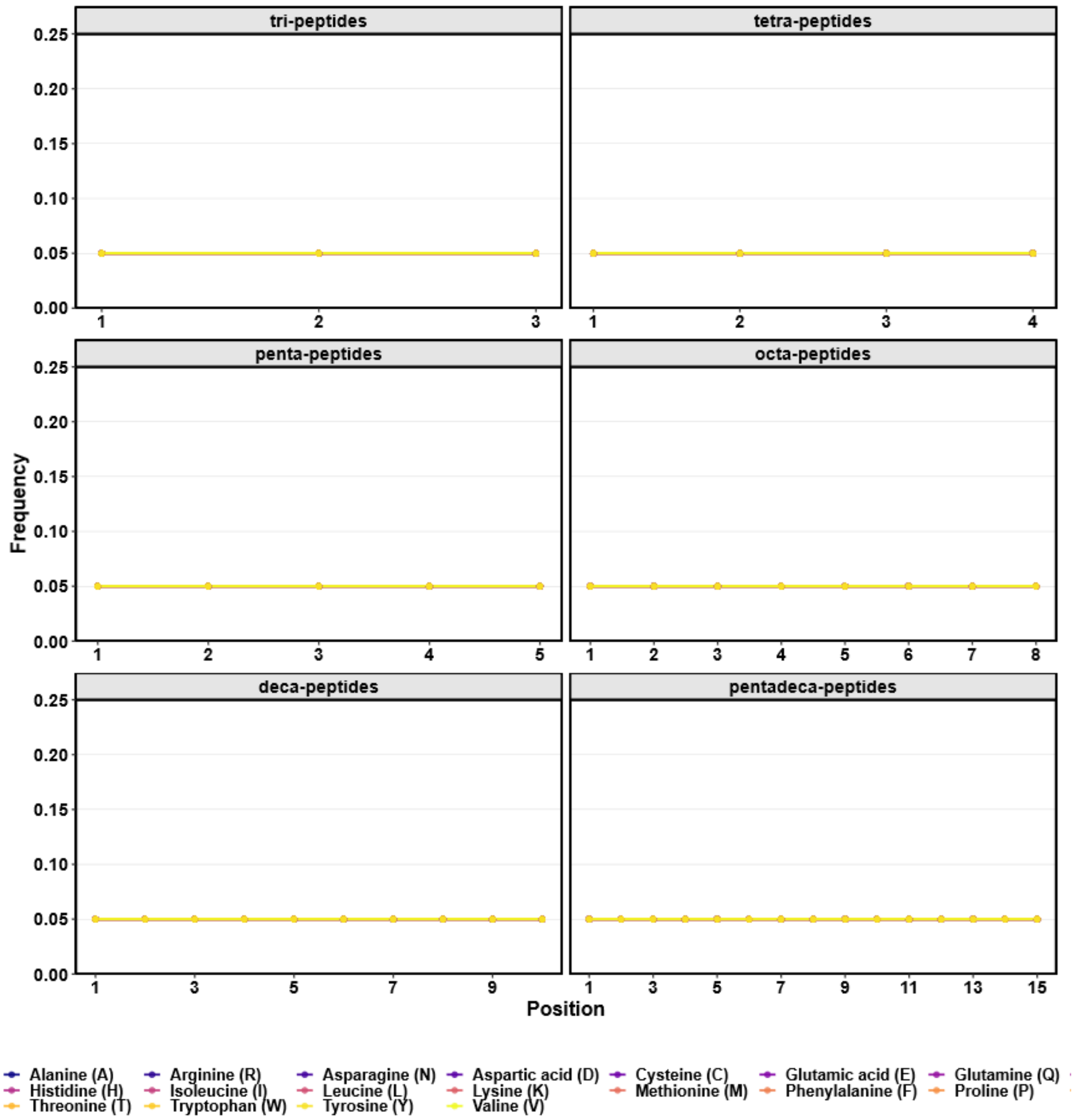

Figure S6. UD sampling ensures that every amino acid occurs at exactly 5% frequency in each position of tri-through pentadeca-peptides (n=1,000 sequences sampled length).

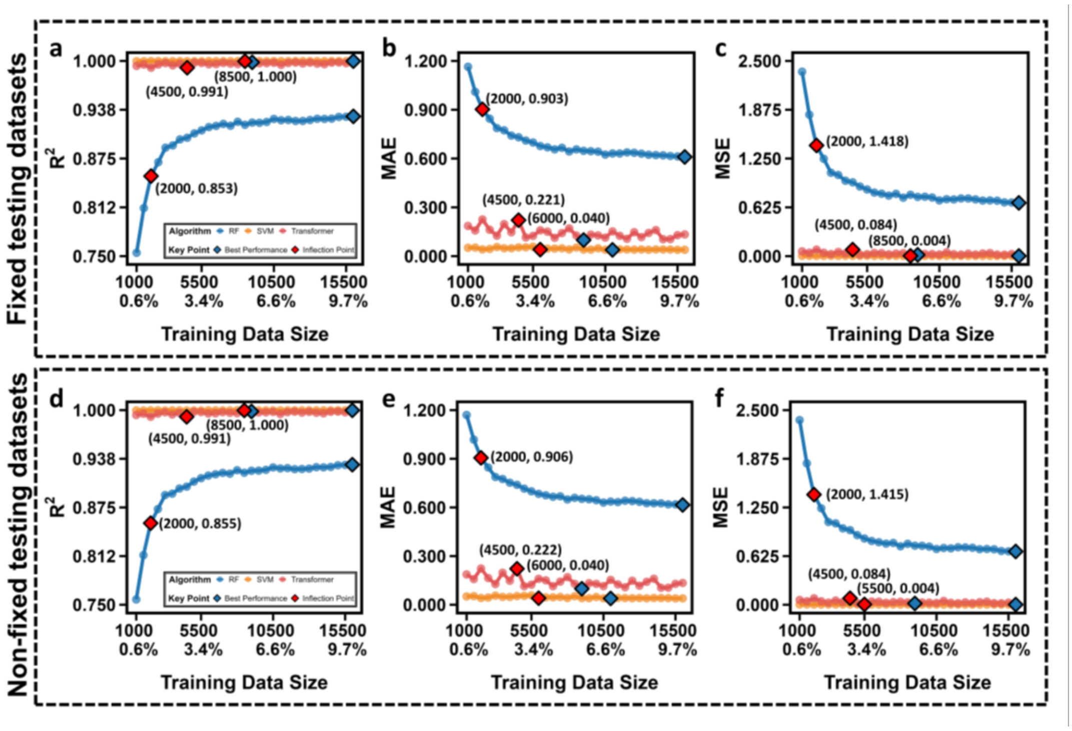

Figure S7. a-c) Performance metrics (R^2, MAE, MSE) of the RF, SVM, and Transformer models in predicting the logP using a fixed testing set, evaluated on models trained with varying training set sizes. **d-f)** Performance metrics (R^2, MAE, MSE) of the RF, SVM, and Transformer models in predicting logP using a non-fixed testing set, evaluated on models trained with different training set sizes.

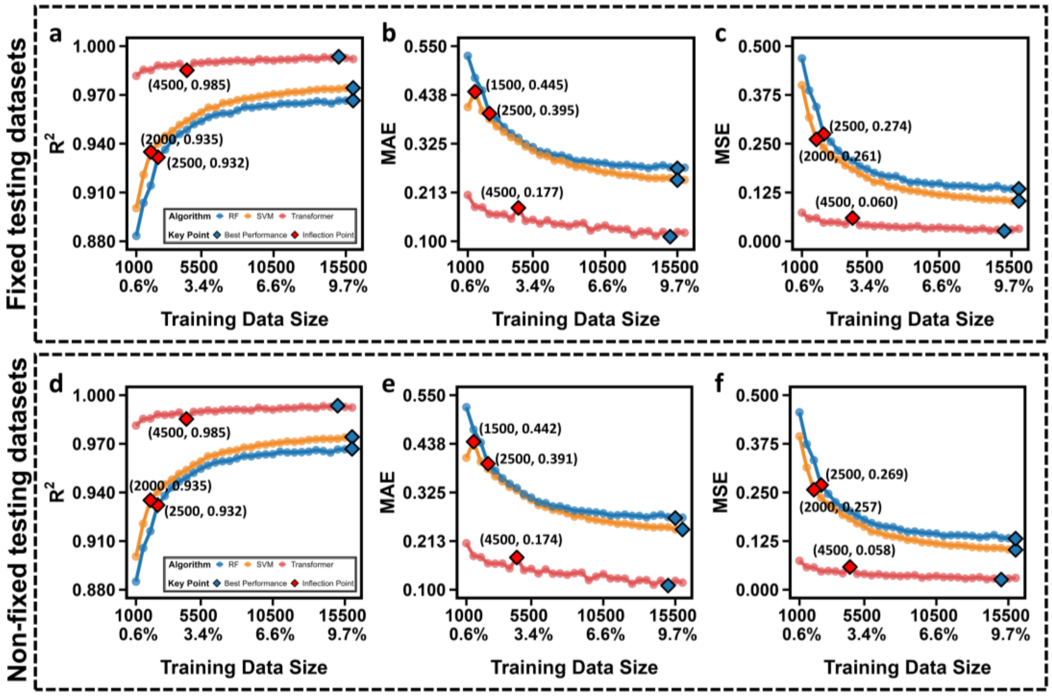

Figure S8. a-c) Performance metrics (R^2, MAE, MSE) of the RF, SVM, and Transformer models in predicting the pI using a fixed testing set, evaluated on models trained with varying training set sizes. **d-f)** Performance metrics (R^2, MAE, MSE) of the RF, SVM, and Transformer models in predicting pI using a non-fixed testing set, evaluated on models trained with different training set sizes.

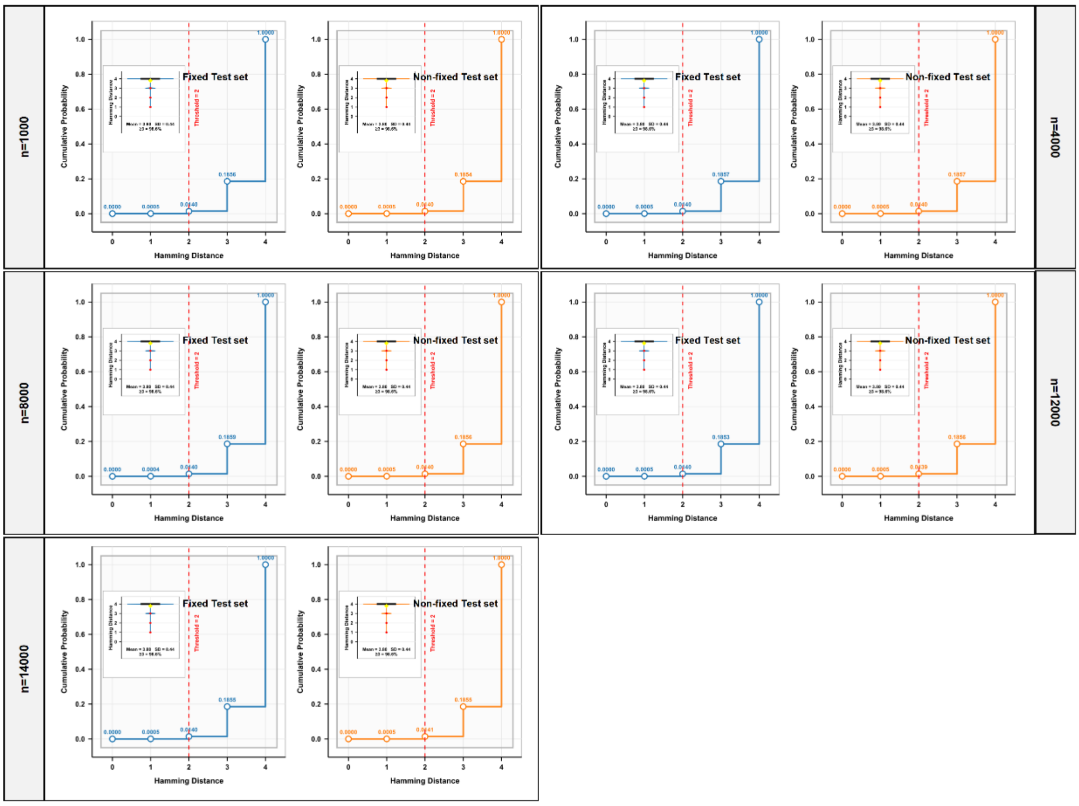

Figure S9. Hamming distance distributions between training and testing tetrapeptide sequences. Summary across five representative training set sizes (n = 1000, 4000, 8000, 12000, and 16000 sequences). (**Left**) Analysis using the fixed testing set. (**Right**) Analysis using the non-fixed (held-out) testing set. In each panel, the step plot shows the cumulative distribution function of Hamming distances (0-4) between training and test sequences. The embedded violin + box plot displays the full distribution of distances, with the yellow diamond marking the mean. The red dashed line indicates a distance of 2 residues. Numerical summaries (mean ± SD and percentage of pairs with distance ≥3) are noted below each plot.

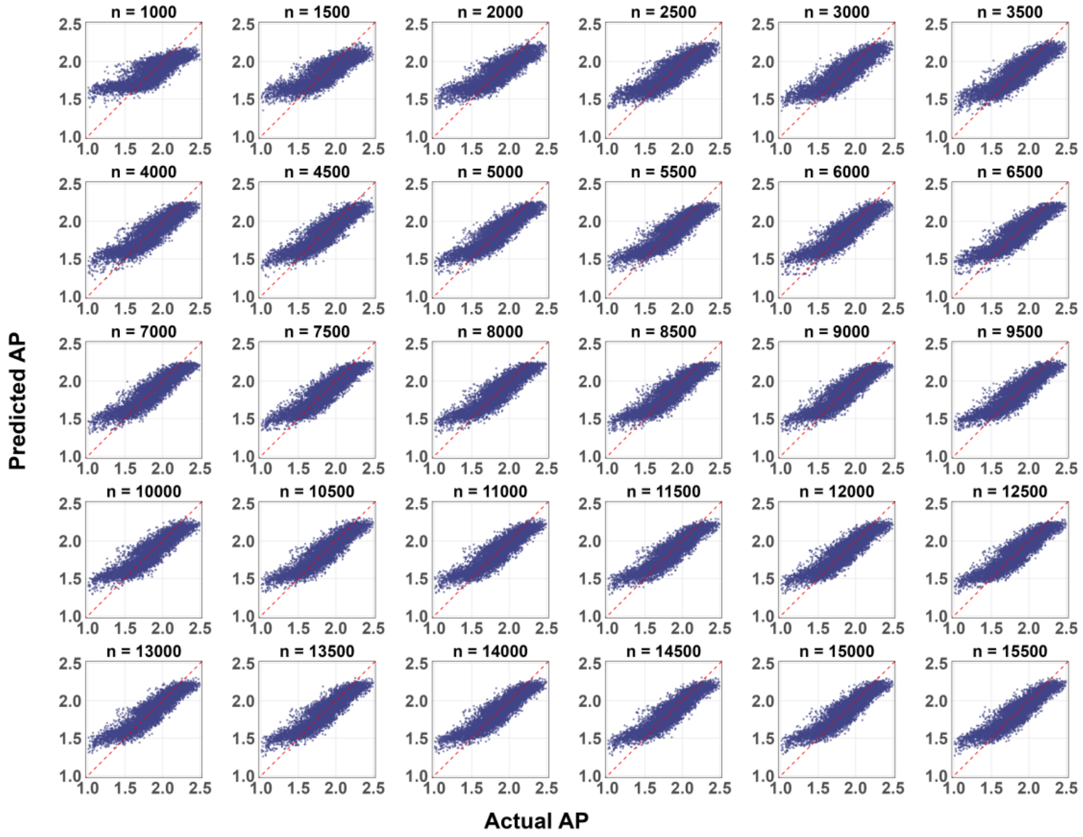

Figure S10. Comparison of actual versus predicted AP values from Random Forest models evaluated using an independent test set of 10,000 samples. The variable n represents the size of the training set (sampled dataset).

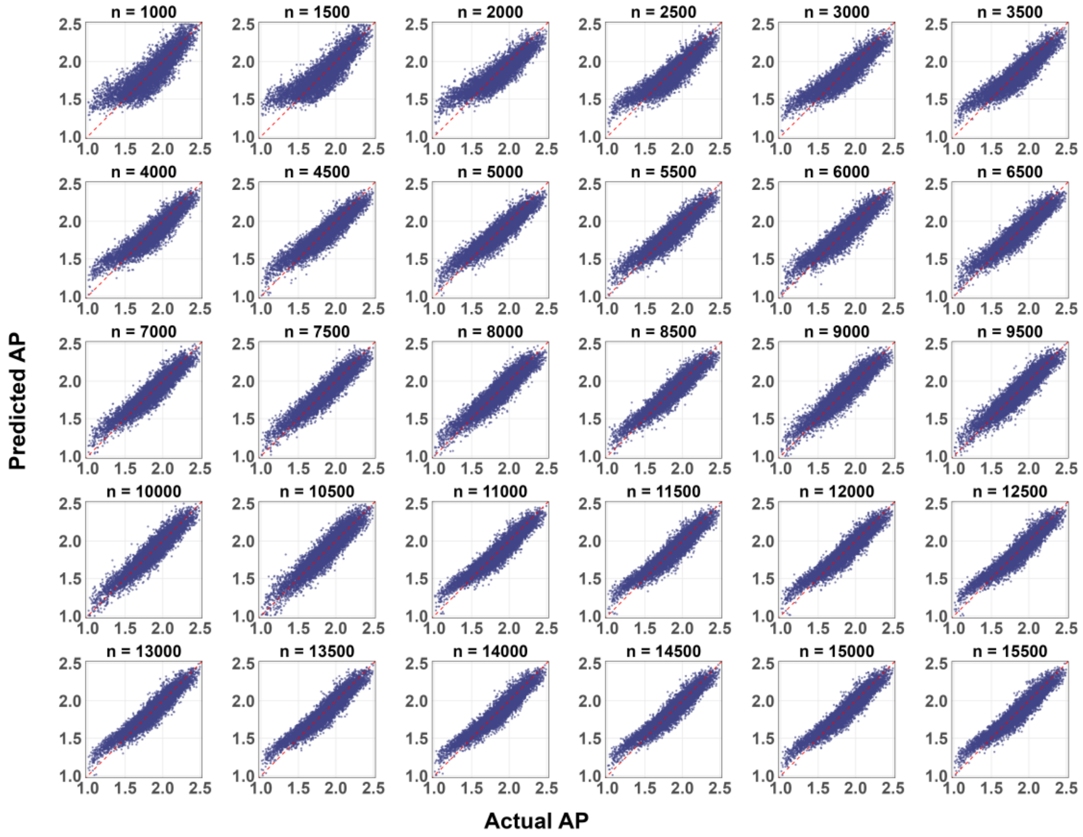

Figure S11. Comparison of actual versus predicted AP values from Support Vector Machine models evaluated using an independent test set of 10,000 samples. The variable n represents the size of the training set (sampled dataset).

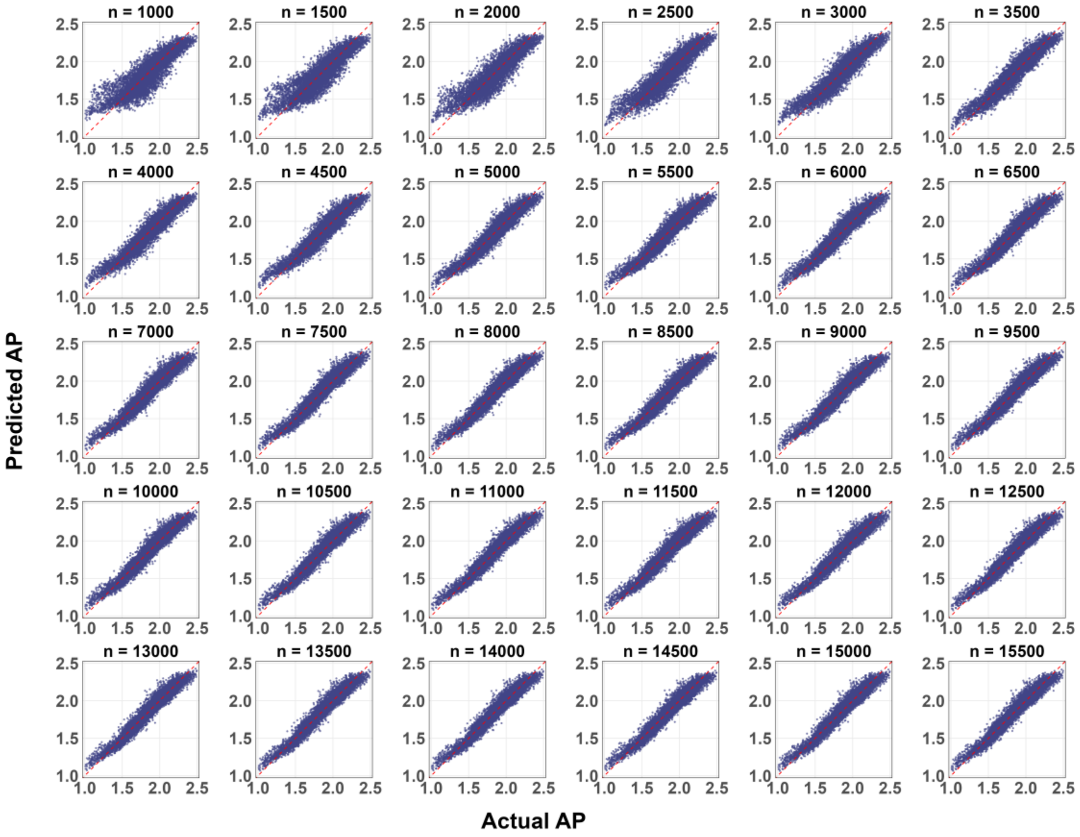

Figure S12. Comparison of actual versus predicted AP values from Transformer models evaluated using an independent test set of 10,000 samples. The variable n represents the size of the training set (sampled dataset).

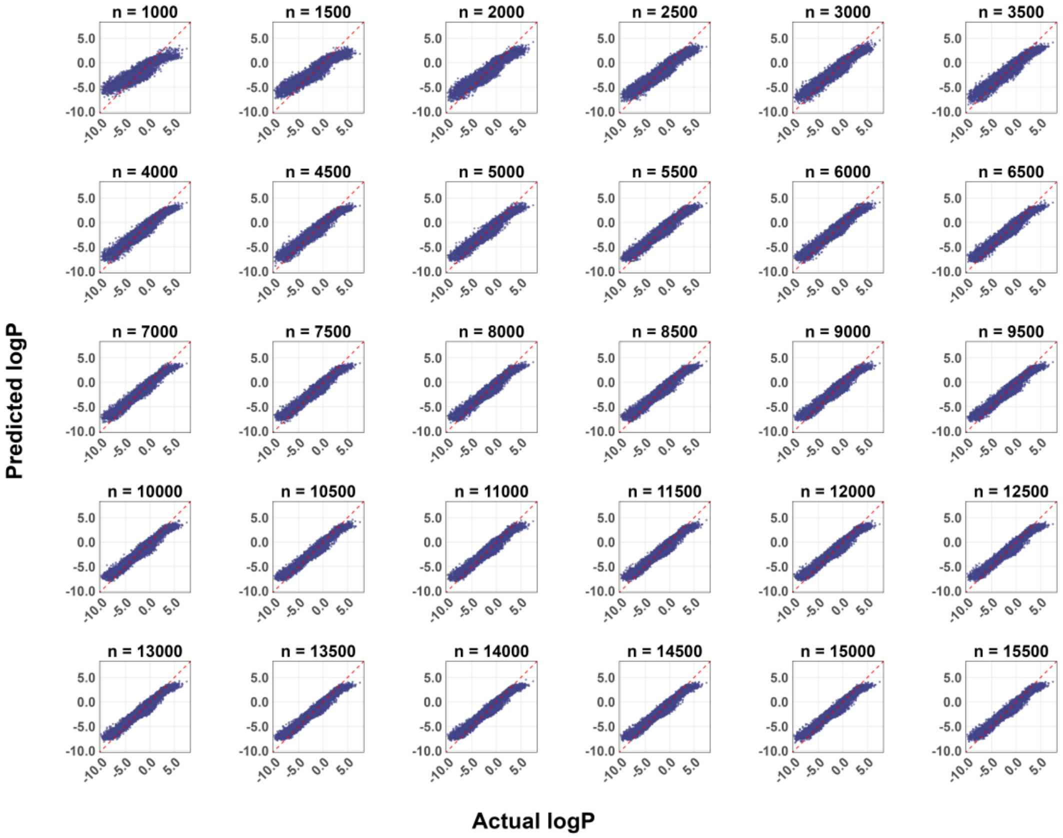

Figure S13. Comparison of actual versus predicted logP values from RF models evaluated using an independent test set of 10,000 samples. The variable n represents the size of the training set (sampled dataset).

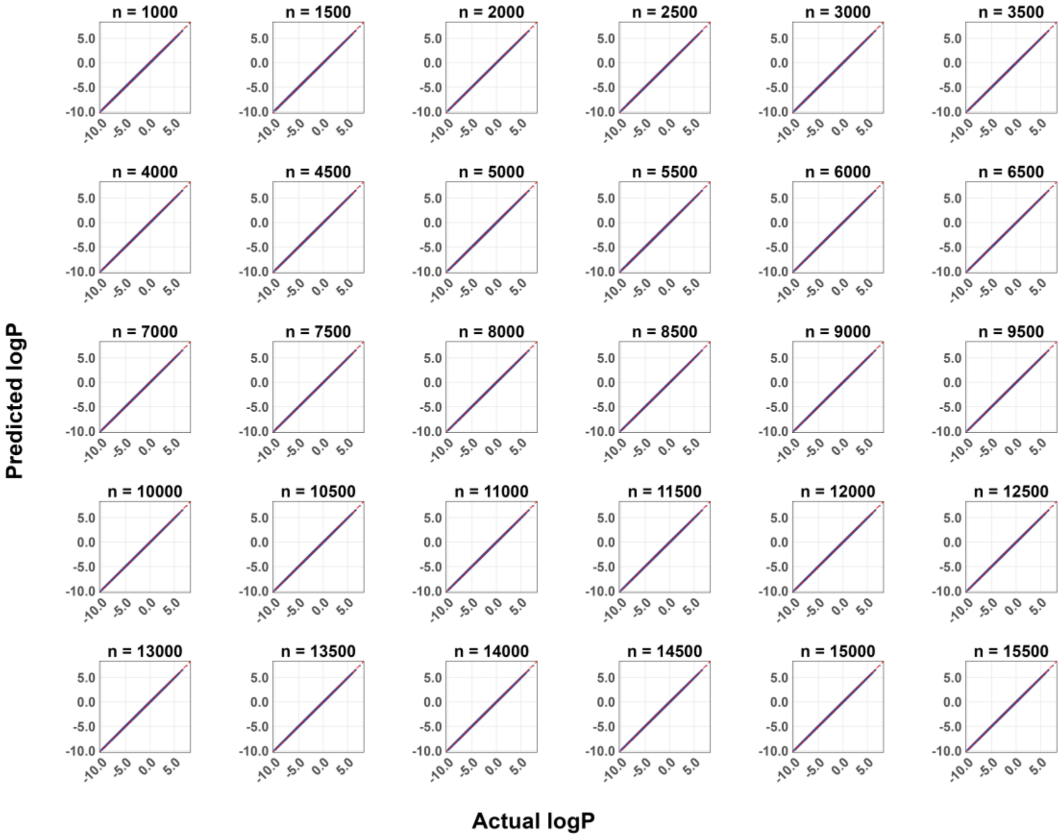

Figure S14. Comparison of actual versus predicted logP values from SVM models evaluated using an independent test set of 10,000 samples. The variable n represents the size of the training set (sampled dataset).

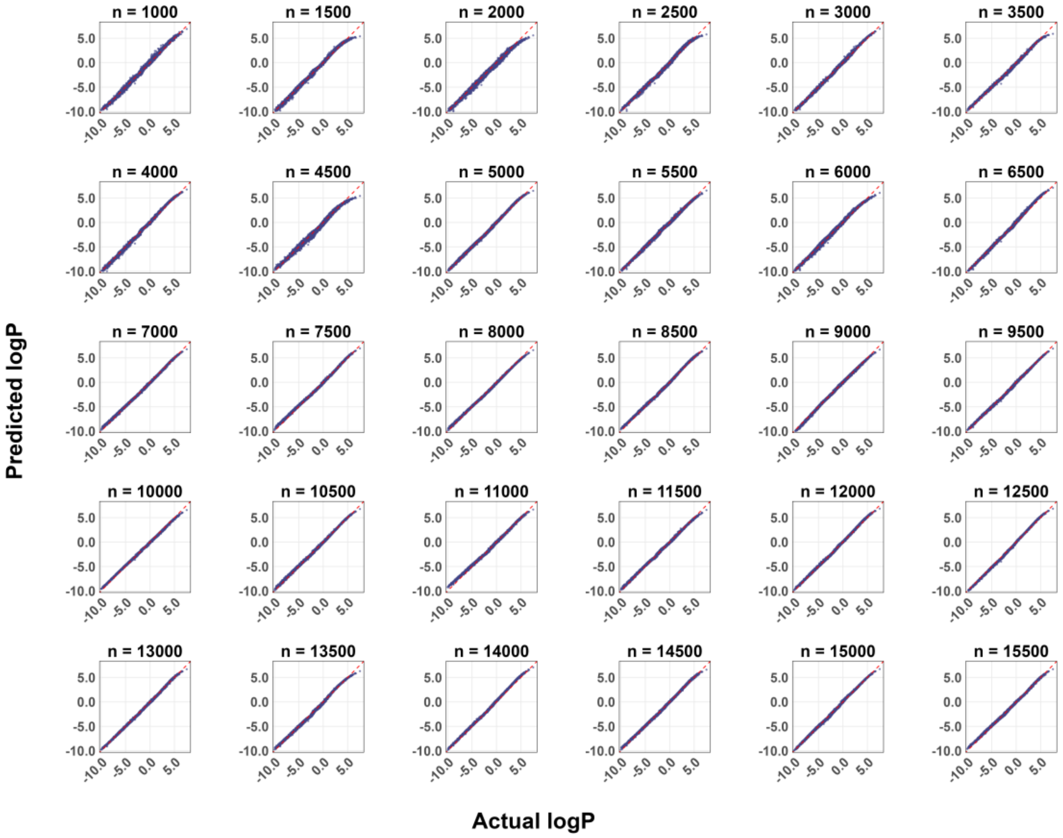

Figure S15. Comparison of actual versus predicted logP values from Transformer models evaluated using an independent test set of 10,000 samples. The variable n represents the size of the training set (sampled dataset).

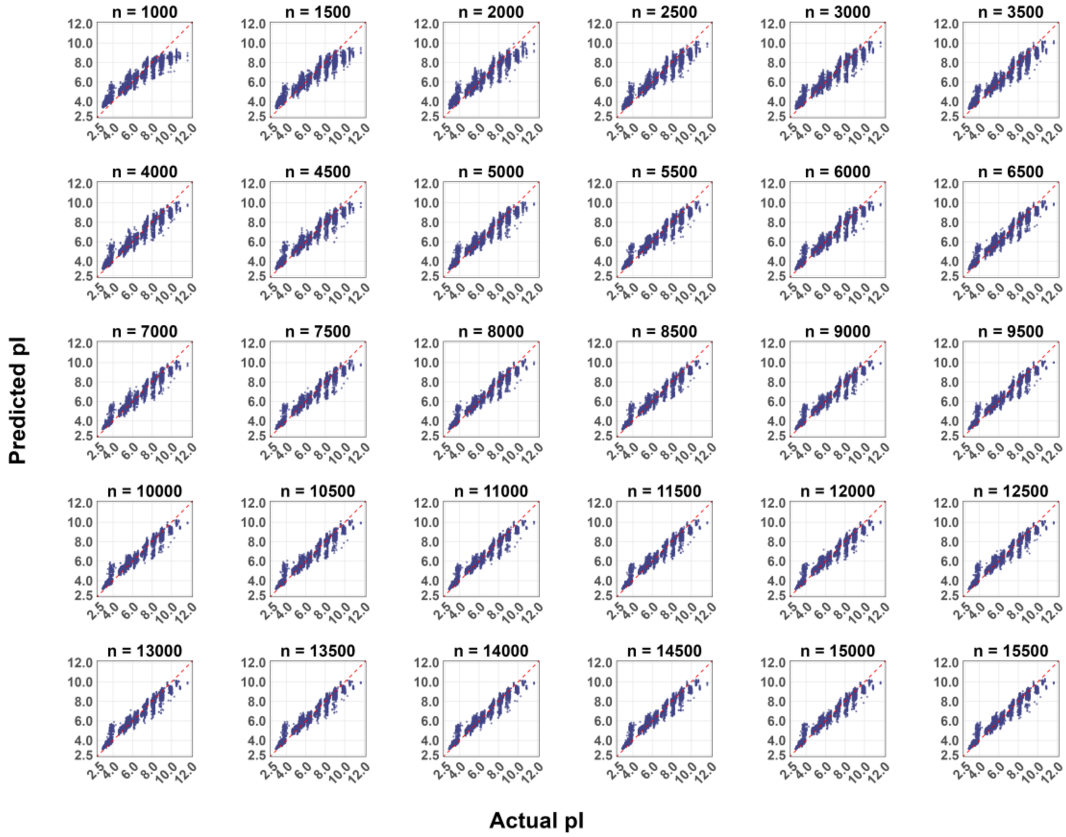

Figure S16. Comparison of actual versus predicted pI values from RF models evaluated using an independent test set of 10,000 samples. The variable n represents the size of the training set (sampled dataset).

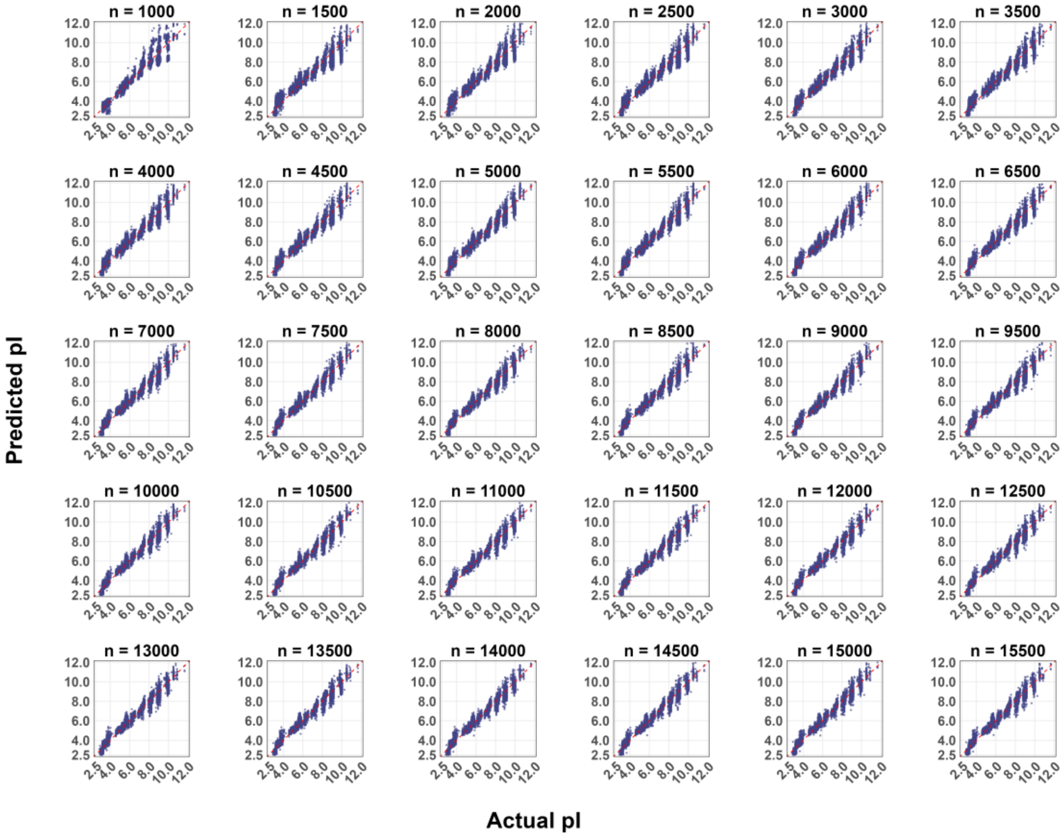

Figure S17. Comparison of actual versus predicted pI values from SVM models evaluated using an independent test set of 10,000 samples. The variable n represents the size of the training set (sampled dataset).

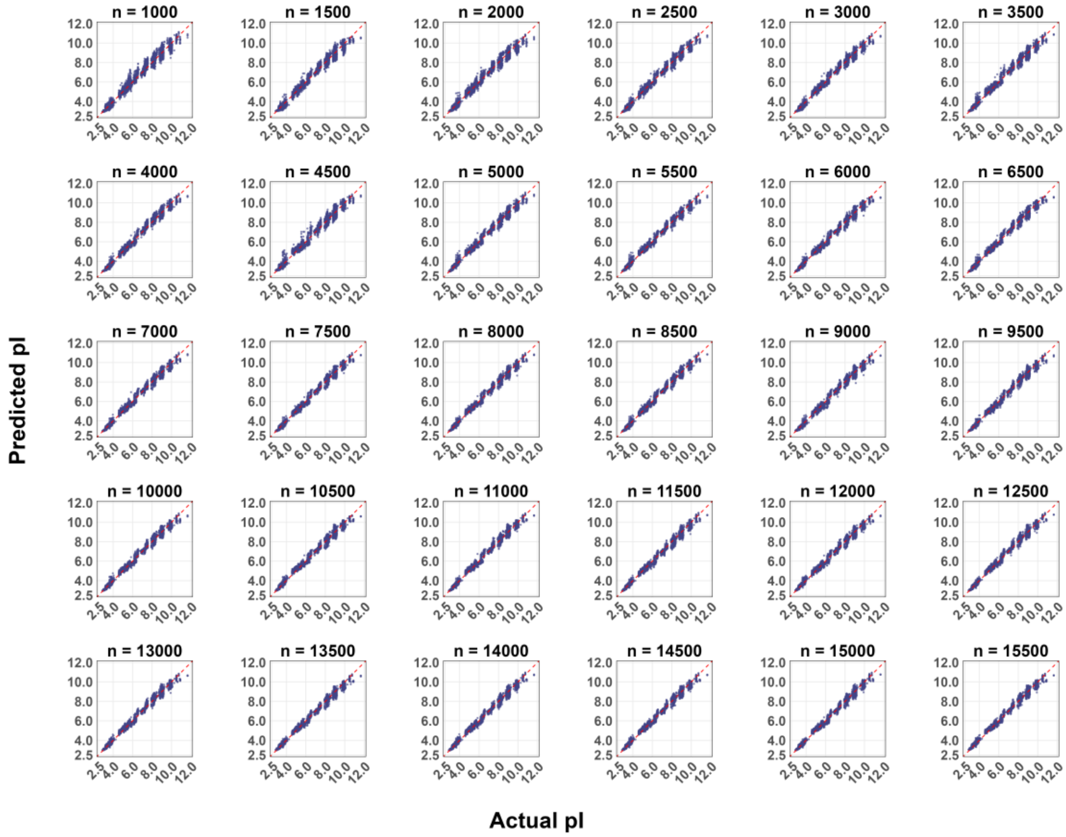

Figure S18. Comparison of actual versus predicted pI values from Transformer models evaluated using an independent test set of 10,000 samples. The variable n represents the size of the training set (sampled dataset).

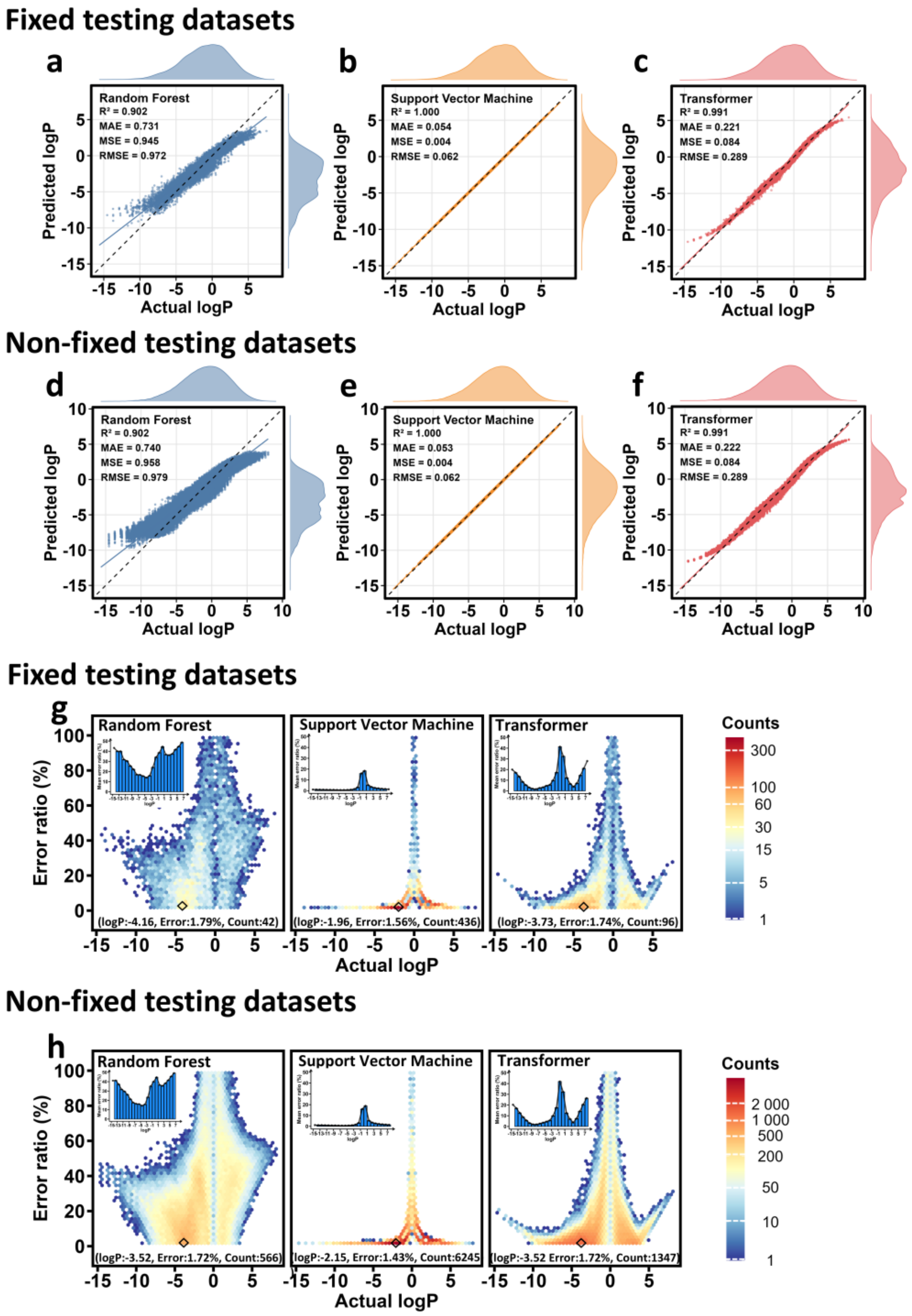

Figure S19. Cross-model prediction error analysis of logP. **a-f)** The relationship between the actual and predicted logP values of the RF, SVM and Transformer models is evaluated using a fixed versus a non-fixed testing dataset with a training set of 4,500 (i.e. the threshold of the Transformer model). **g-h)** The relationship between actual logP values and percentage prediction error is visualized by a hexagonal fractal plot when evaluated using a fixed versus a non-fixed testing dataset with the same amount of data in the training set, thus capturing the density of points in the dataset of the three combinations of prediction models.

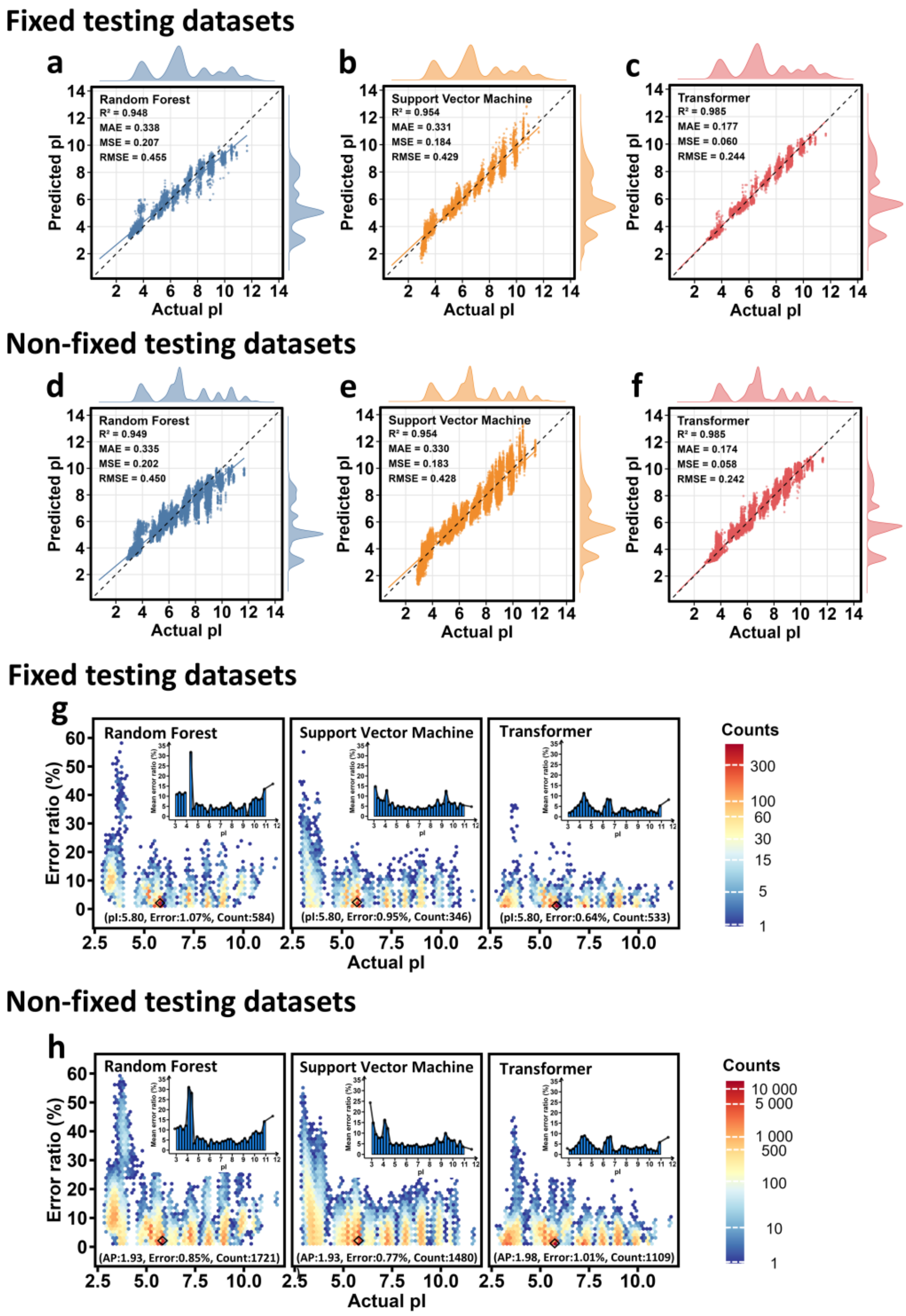

Figure S20. Cross-model prediction error analysis of pI. **a-f)** The relationship between the actual and predicted pI values of the RF, SVM and Transformer models is evaluated using a fixed versus a non-fixed testing dataset with a training set of 4,500 (i.e. the threshold of the Transformer model). **g-h)** The relationship between actual pI values and the percentage prediction error is visualized by a hexagonal fractal plot when evaluated using a fixed versus a non-fixed testing dataset with the same amount of data in the training set, thus capturing the density of points in the dataset of the three combinations of prediction models.

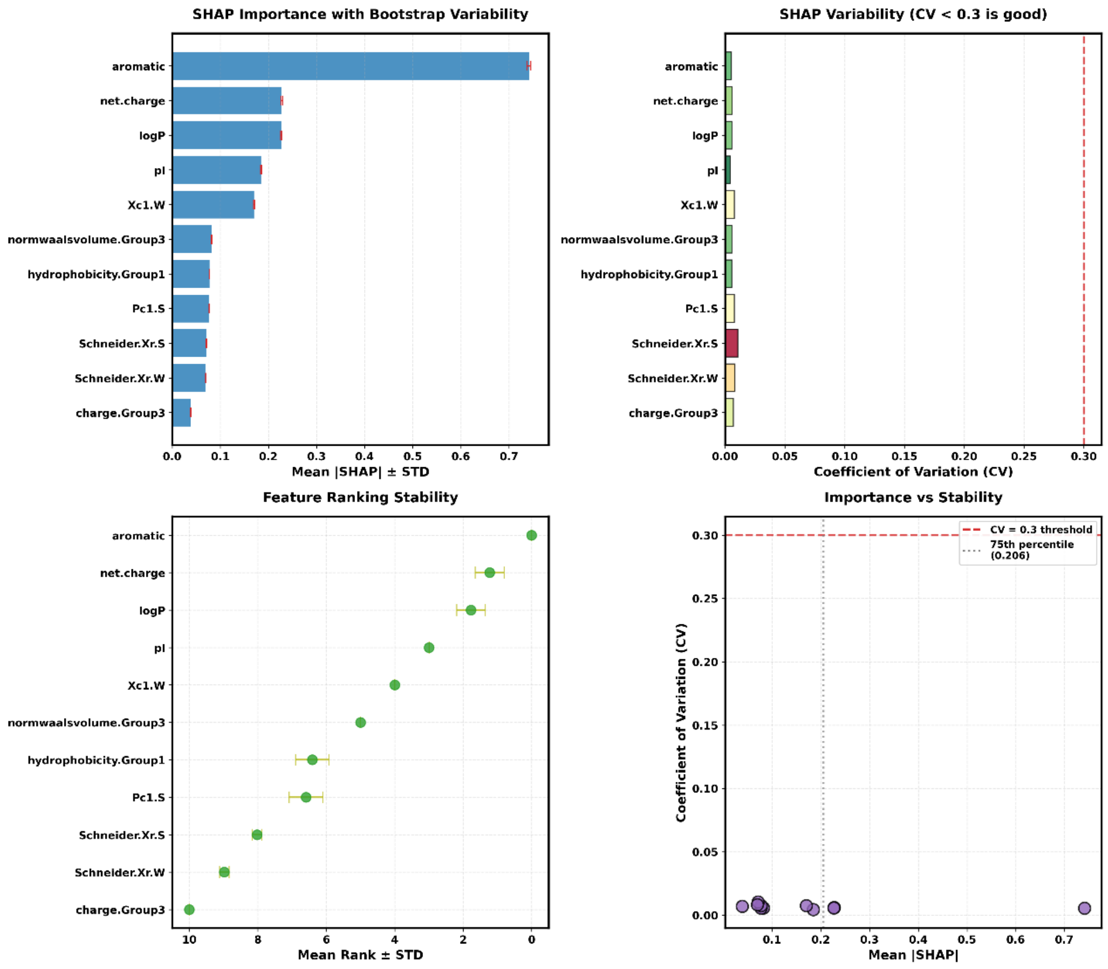

Figure S21. Bootstrap stability assessment of SHAP values for the LightGBM model (Class 1 - optimal aggregation). **(Top-left):** Mean absolute SHAP values (± standard deviation) across 100 bootstrap iterations, ranked from highest to lowest. **(Top-right):** Coefficient of Variation (CV) for each feature; the red dashed line indicates the 0.3 threshold for high variability. **(Bottom-left):** Feature ranking stability: mean rank (± standard deviation) across bootstrap iterations (rank 1 is most important). **(Bottom-right):** Relationship between feature importance (mean |SHAP|) and stability (CV). All features cluster in the high-importance, low-variability region.

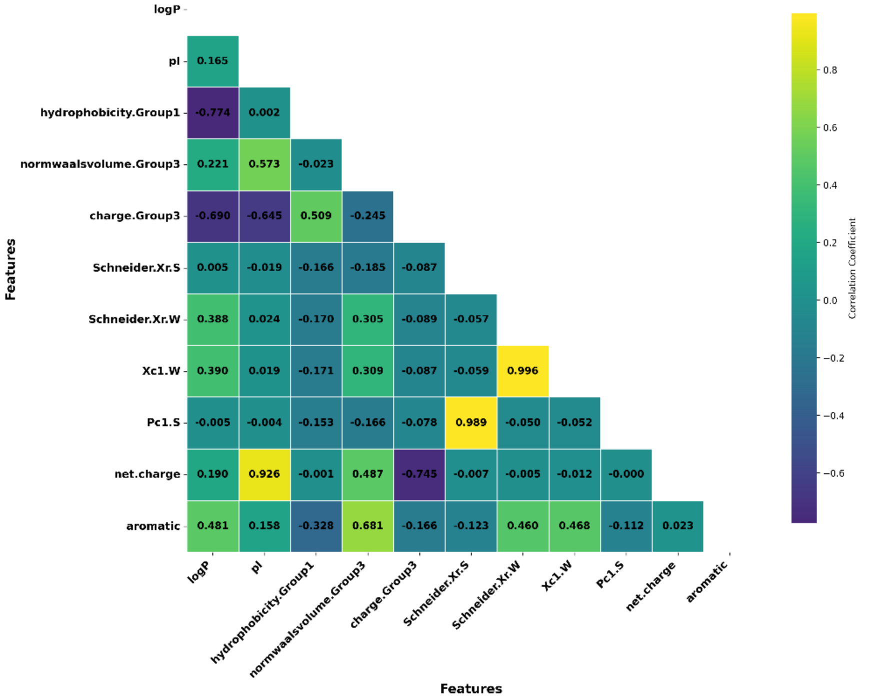

Figure S22. Feature correlation matrix for the 11 selected descriptors used in tree-based model analysis. Pairwise Pearson correlation coefficients are displayed in the upper triangle.

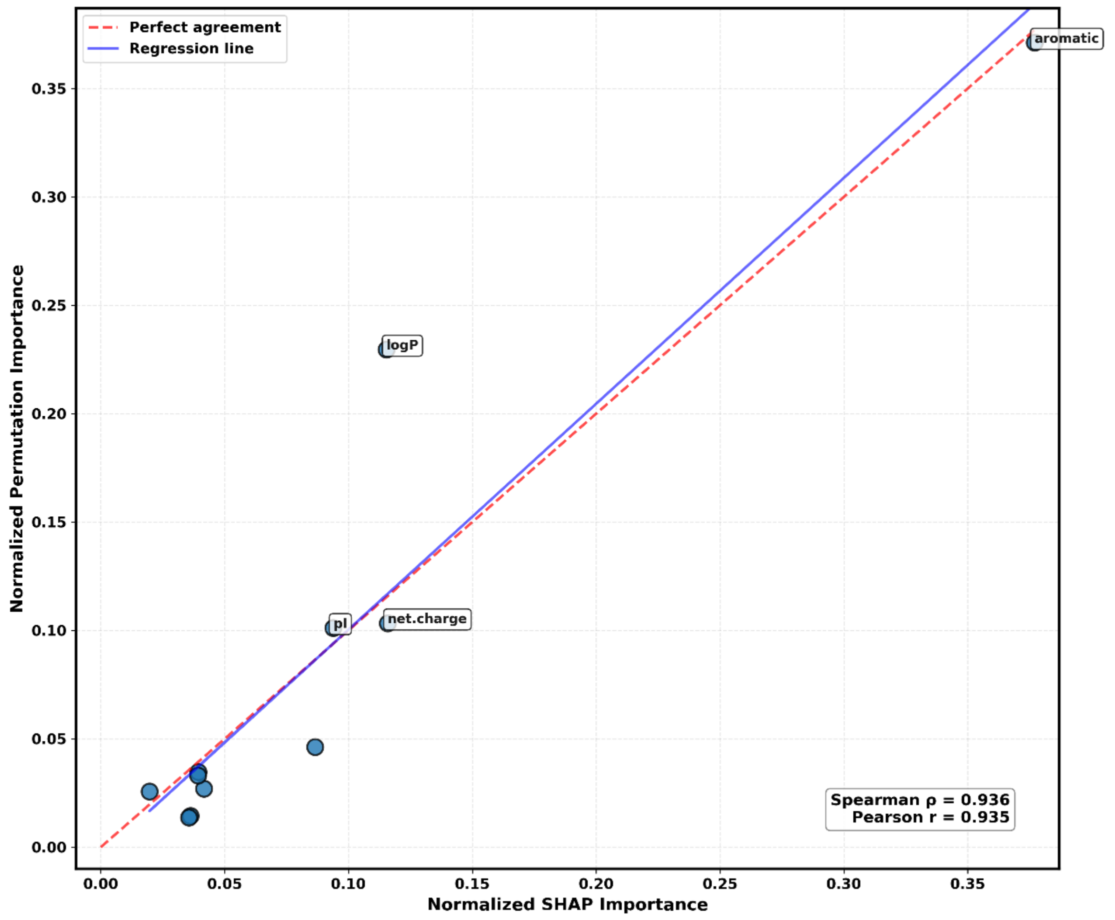

Figure S23. Correlation between normalized SHAP importance and normalized permutation importance for the 11 selected features. The dashed red line indicates perfect agreement. The solid blue line shows the linear regression fit. The Spearman rank correlation (ρ = 0.936) and Pearson correlation (r = 0.932) are displayed in the lower right, indicating excellent consistency between the two distinct importance metrics.

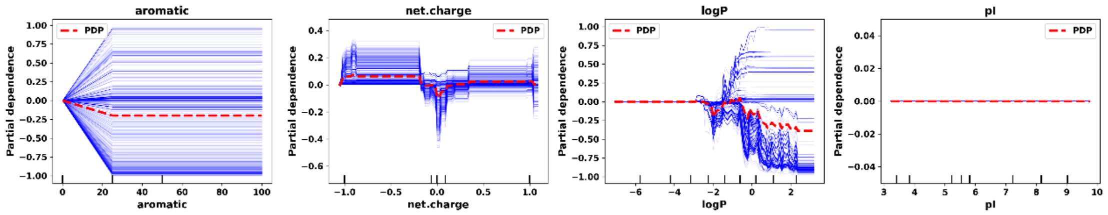

Figure S24. Partial Dependence Plots (PDPs) and Individual Conditional Expectation (ICE) plots for the four most important features influencing Class 1 aggregation (LightGBM model). For each feature (aromatic, logP, pI, net.charge), the thick red line represents the PDP (average marginal effect), and the thin blue lines represent a subset of ICE curves (individual conditional predictions).

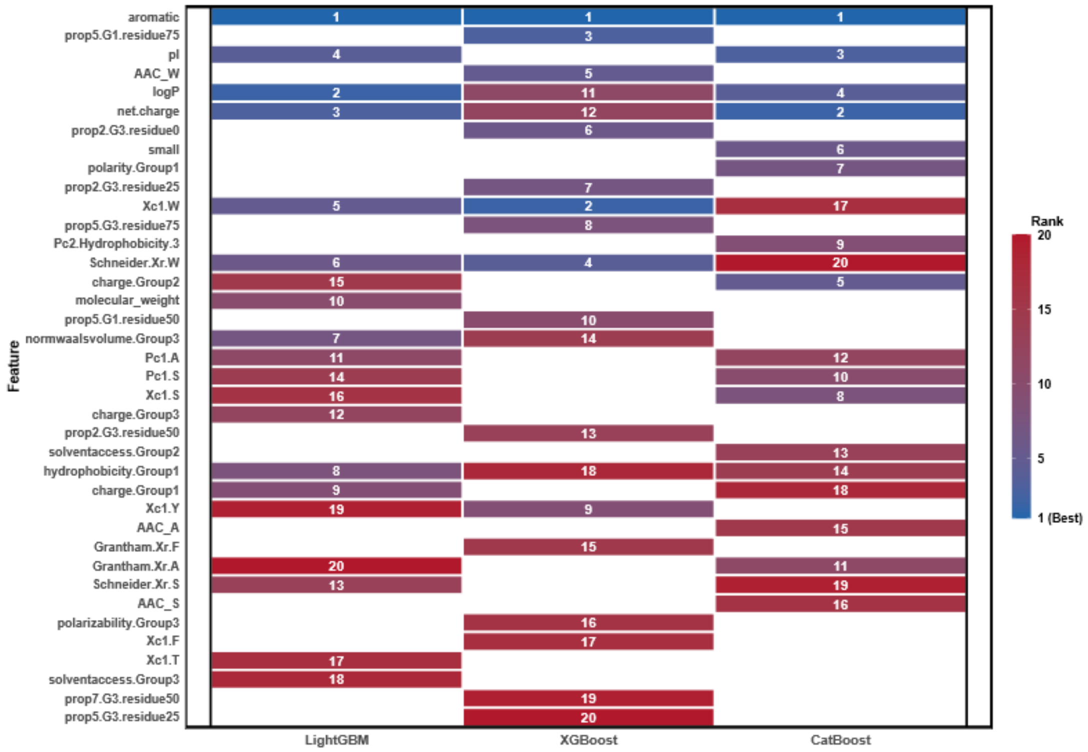

Figure S25. Heatmap of top 20 feature rankings across models. The heatmap compares the rankings of the top 20 most important features (rows) for predicting tetrapeptide aggregation propensity, as identified by the LightGBM, XGBoost, and CatBoost models (columns). Cell color and number indicate the rank within each model (darker red = higher importance).

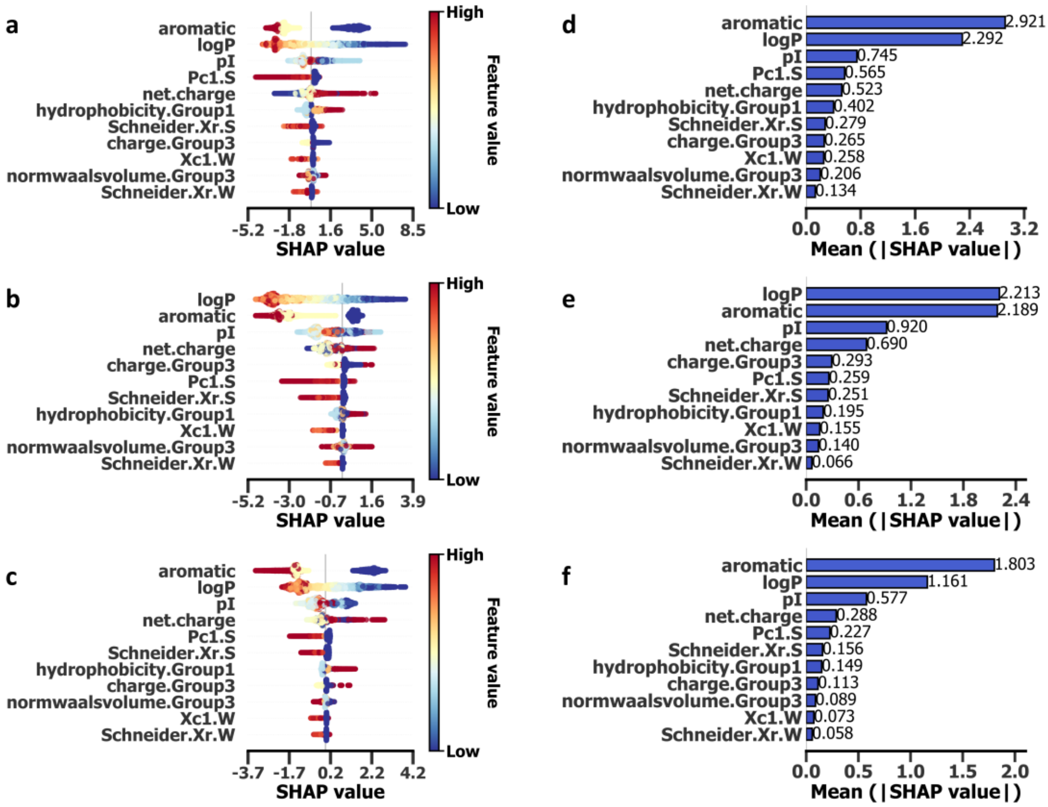

Figure S26. Comprehensive SHAP analysis for tetrapeptides' AP values in Class 0. **a-c)** Beeswarm plots illustrating the global distribution of features for Class 0 classification across the three models (LightGBM, XGBoost, CatBoost, from top to bottom). Each point represents a sample's SHAP value, with colors indicating feature values (red for high, blue for low). The horizontal position denotes the SHAP value magnitude (negative values indicate negative impact and positive values indicate positive impact), while vertical stacking indicates a higher density of SHAP values in that region. The features are ranked in descending order of importance from top to bottom. **d-f)** The summary bar plot displays the mean absolute SHAP values for 11 features, ranked in descending order of importance. These values represent the average impact of features on predictions, with results corresponding to LightGBM, XGBoost, and CatBoost presented sequentially from top to bottom.

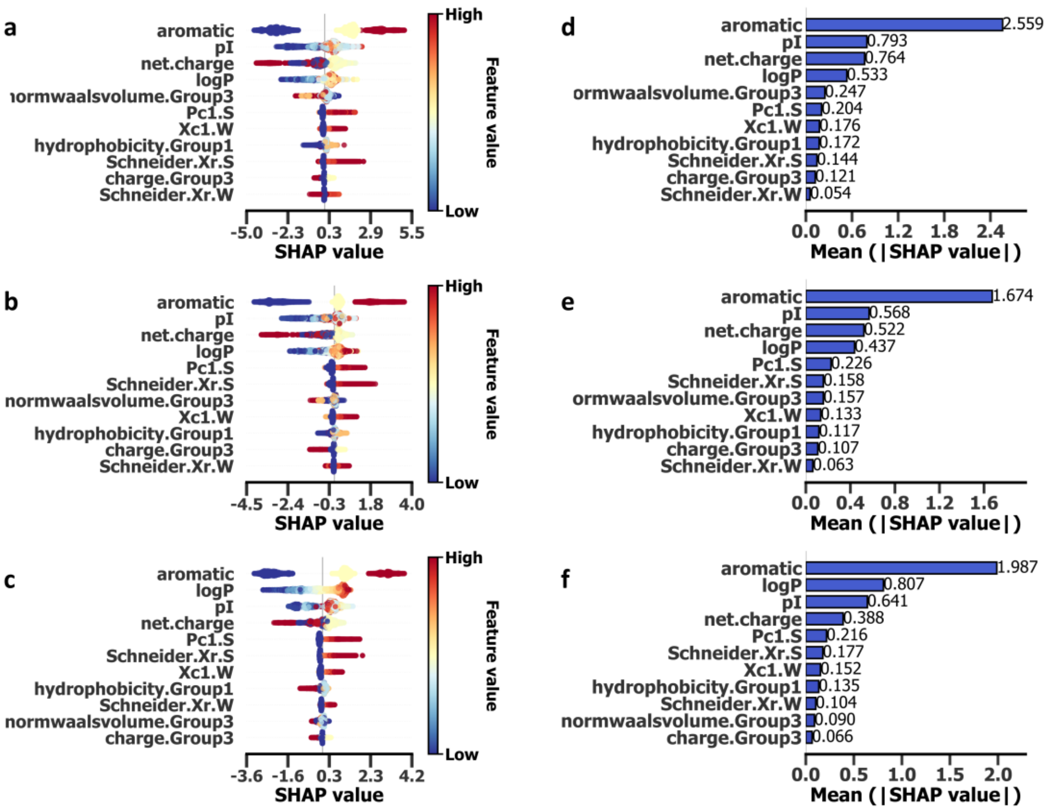

Figure S27. Comprehensive SHAP analysis for tetrapeptides' AP values in Class 2. **a-c)** Beeswarm plots illustrating the global distribution of features for Class 2 classification across the three models (LightGBM, XGBoost, CatBoost, from top to bottom). Each point represents a sample's SHAP value, with colors indicating feature values (red for high, blue for low). The horizontal position denotes the SHAP value magnitude (negative values indicate negative impact and positive values indicate positive impact), while vertical stacking indicates a higher density of SHAP values in that region. The features are ranked in descending order of importance from top to bottom. d-f) The summary bar plot displays the mean absolute SHAP values for 11 features, ranked in descending order of importance. These values represent the average impact of features on predictions, with results corresponding to LightGBM, XGBoost, and CatBoost presented sequentially from top to bottom.

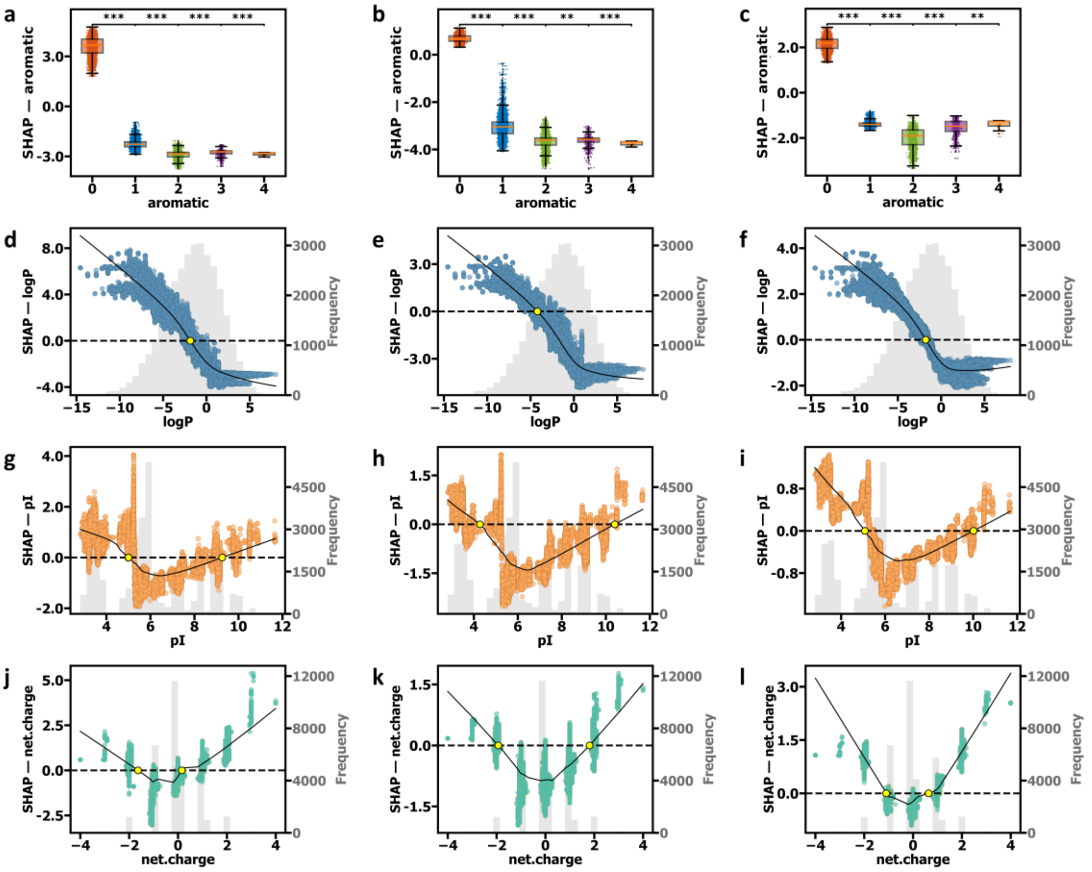

Figure S28. SHAP dependence plots for top features in Class 0 tetrapeptide sequences. **a-c)** Impact of aromatic residue count on Class 0 SHAP values for LightGBM, XGBoost, and CatBoost models. Box plots show SHAP value distributions for different aromatic residue counts (0, 1, 2, 3, and 4). Boxes represent interquartile ranges, middle lines indicate medians, and whiskers extend to 1.5 times the interquartile range. Asterisks or NS (none-significance) above box plots indicate statistical significance levels of SHAP value differences between adjacent groups. Based on Mann-Whitney U tests with Bonferroni correction, significance levels are: *** $p < 0.00025$ (0.001/4), ** $p < 0.0025$ (0.01/4), * $p < 0.0125$ (0.05/4), NS: $p \geq 0.0125$ (not significant). **d-f)** SHAP dependence plots for the logP feature in Class 0 for LightGBM, XGBoost, and CatBoost models. Gray histograms (referring to the right axes) reflect the frequency distribution of logP values in the testing dataset. Black LOWESS (Locally Weighted Scatterplot Smoothing) fitting curves show the overall trend between logP and SHAP values. Black dashed lines indicate the SHAP value of 0 baselines, while yellow markers highlight critical values where the impact of logP on predictions shifts from positive to negative (or vice versa). **g-i)** SHAP dependence plots for the pI feature in Class 0 for LightGBM, XGBoost, and CatBoost models. These plots include scatter points, gray histograms, and black LOWESS curves, similar to d-f. Critical values are marked by yellow points where the impact of pI shifts. **j-l)** SHAP dependence plots for the net charge feature in Class 0 for LightGBM, XGBoost, and CatBoost models. These plots include scatter points, gray histograms, black LOWESS curves, and intersection annotations to comprehensively show how net charge affects model predictions.

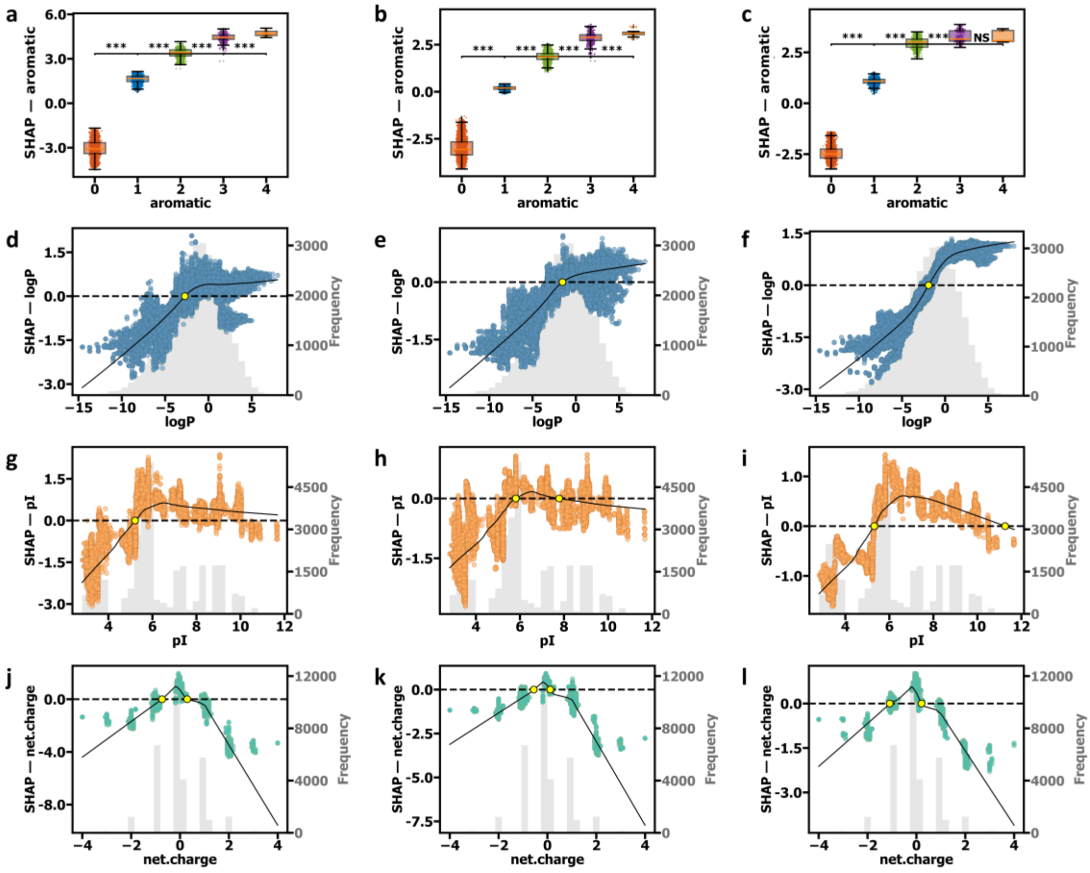

Figure S29. SHAP dependence plots for top features in Class 2 tetrapeptide sequences. **a-c)** Impact of aromatic residue count on Class 2 SHAP values for LightGBM, XGBoost, and CatBoost models. Box plots show SHAP value distributions for different aromatic residue counts (0, 1, 2, 3, and 4). Boxes represent interquartile ranges, middle lines indicate medians, and whiskers extend to 1.5 times the interquartile range. Asterisks or NS (none-significance) above box plots indicate statistical significance levels of SHAP value differences between adjacent groups. Based on Mann-Whitney U tests with Bonferroni correction, significance levels are: *** $p < 0.00025$ (0.001/4), ** $p < 0.0025$ (0.01/4), * $p < 0.0125$ (0.05/4), NS: $p \geq 0.0125$ (not significant). **d-f)** SHAP dependence plots for the logP feature in Class 1 for LightGBM, XGBoost, and CatBoost models. Gray histograms (referring to the right axes) reflect the frequency distribution of logP values in the testing dataset. Black LOWESS (Locally Weighted Scatterplot Smoothing) fitting curves show the overall trend between logP and SHAP values. Black dashed lines indicate the SHAP value of 0 baselines, while yellow markers highlight critical values where the impact of logP on predictions shifts from positive to negative (or vice versa). **g-i)** SHAP dependence plots for the pI feature in Class 2 for LightGBM, XGBoost, and CatBoost models. These plots include scatter points, gray histograms, and black LOWESS curves, similar to d-f. Critical values are marked by yellow points where the impact of pI shifts. **j-l)** SHAP dependence plots for the net charge feature in Class 2 for LightGBM, XGBoost, and CatBoost models. These plots include scatter points, gray histograms, black LOWESS curves, and intersection annotations to comprehensively show how net charge affects model predictions.

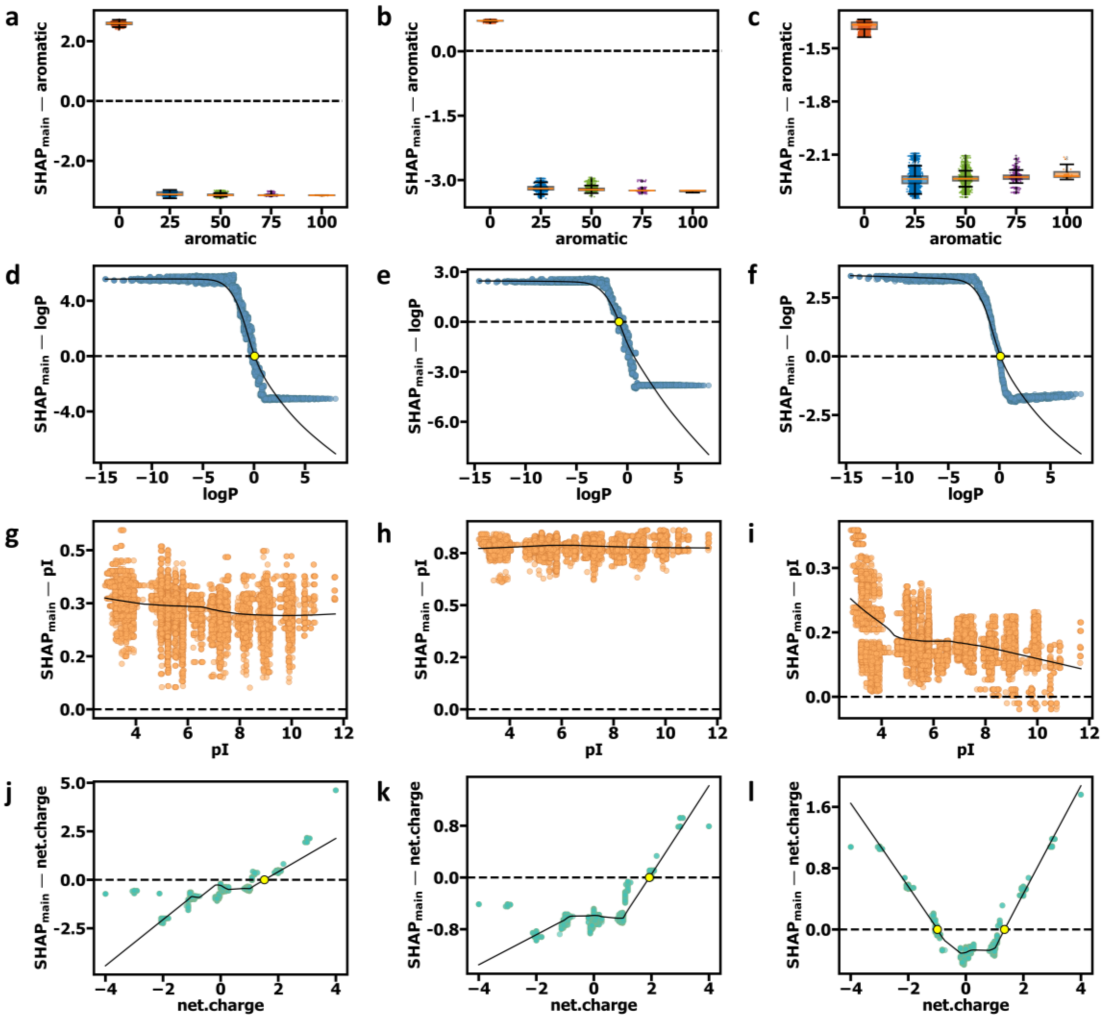

Figure S30. Main effect SHAP dependence plots for top features in Class 0 of AP. **a-c)** SHAP main effect dependence plots for the aromatic property on Class 0 results in the LightGBM, XGBoost, and CatBoost models, from left to right. **d-f)** SHAP main effect dependence plots for the logP feature on Class 0 results in the LightGBM, XGBoost, and CatBoost models, from left to right. **g-i)** SHAP main effect dependence plots for the pI feature on Class 0 results in the LightGBM, XGBoost, and CatBoost models, from left to right. **j-l)** SHAP main effect dependence plots for the net charge feature on Class 0 results. The black LOWESS curve in each Figure Sdepicts the overall trend between different properties and SHAP main effect values.

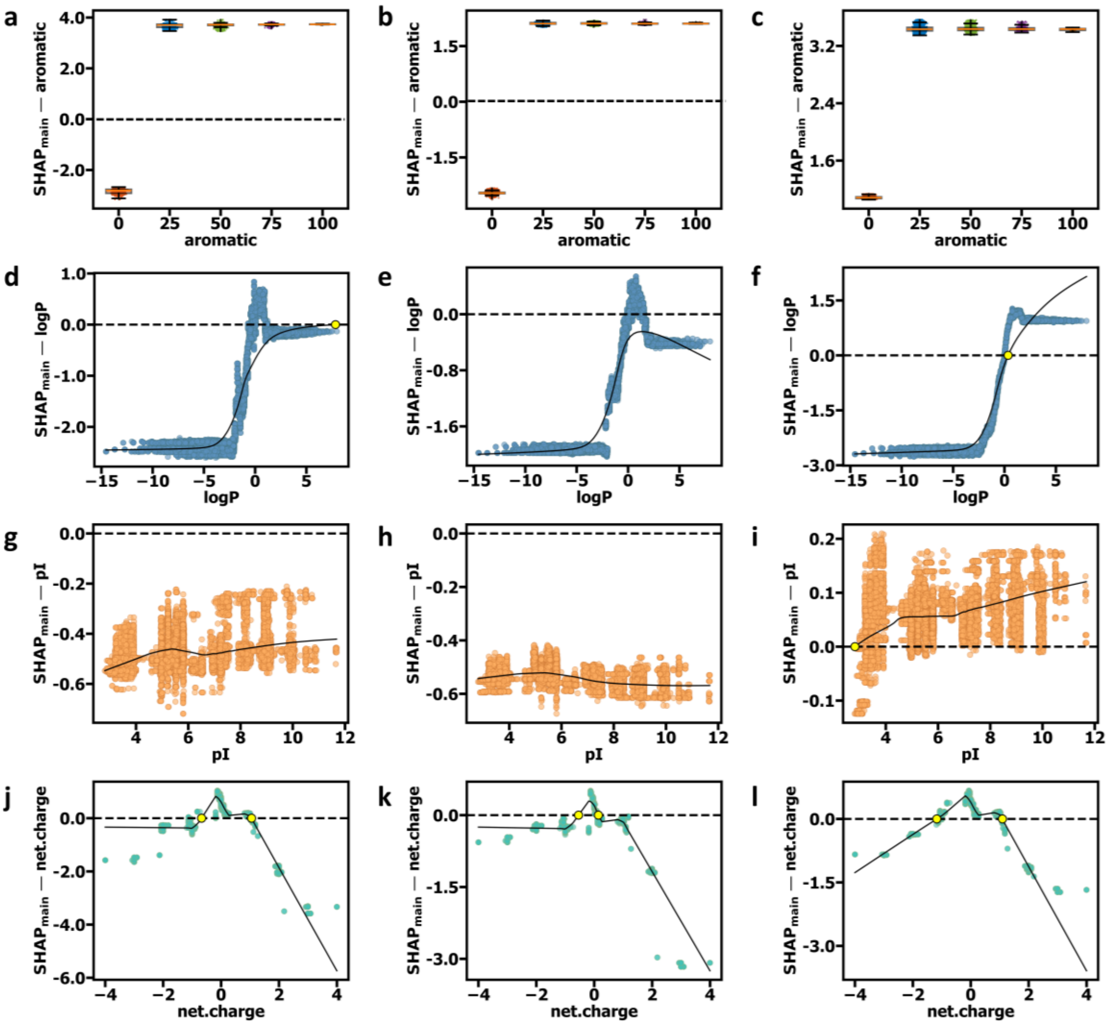

Figure S31. Main effect SHAP dependence plots for top features in Class 2 of AP. **a-c)** SHAP main effect dependence plots for the aromatic property on Class 2 results in the LightGBM, XGBoost, and CatBoost models, from left to right. **d-f)** SHAP main effect dependence plots for the logP feature on Class 2 results in the LightGBM, XGBoost, and CatBoost models, from left to right. **g-i)** SHAP main effect dependence plots for the pI feature on Class 2 results in the LightGBM, XGBoost, and CatBoost models, from left to right. **j-l)** SHAP main effect dependence plots for the net charge feature on Class 2 results. The black LOWESS curve in each Figure Sdepicts the overall trend between different properties and SHAP main effect values.

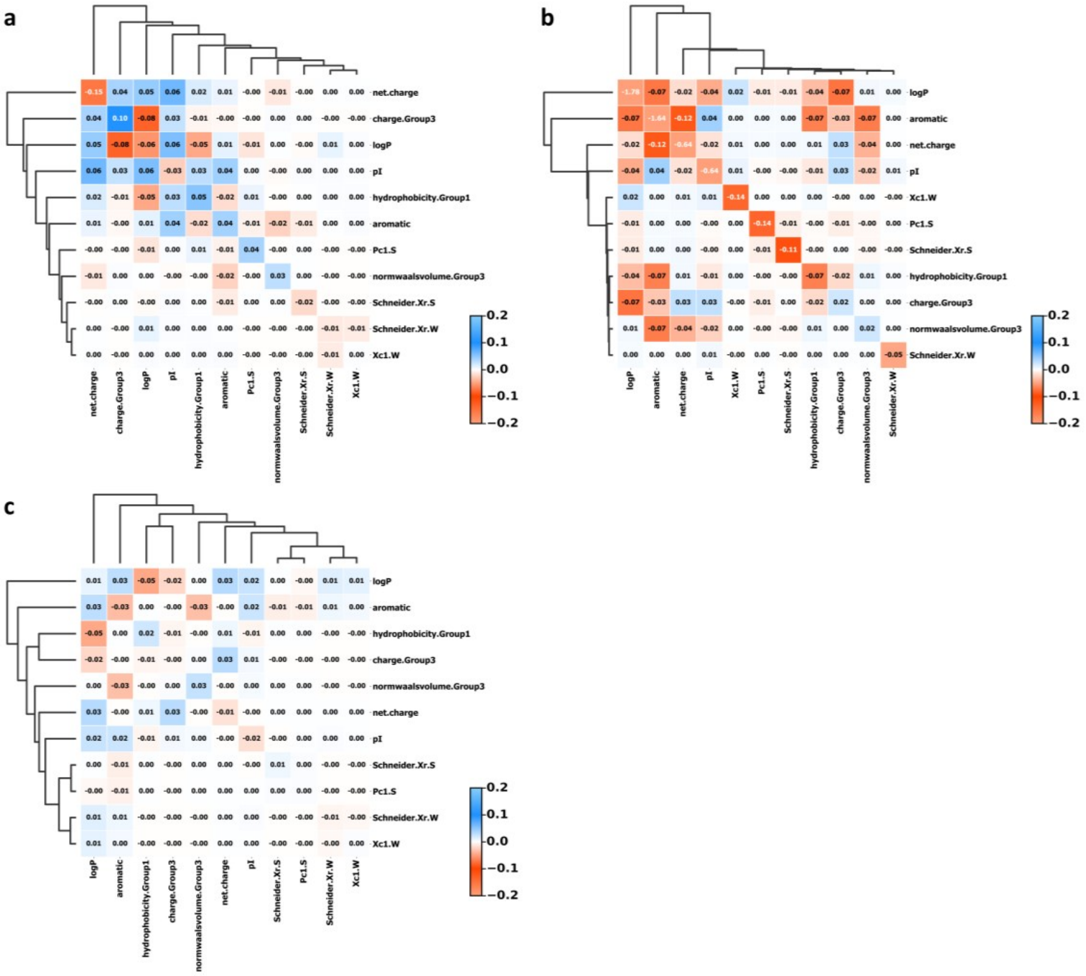

Figure S32. Heatmaps of SHAP interaction values for 11 features in Class 0. **a-c)** The non-diagonal elements quantitatively represent the strength and direction (positive in blue, negative in orange) of interaction effects between features, with diagonal elements replaced by the main effect values of individual features. The figures are arranged in the following order: LightGBM, XGBoost, and CatBoost.

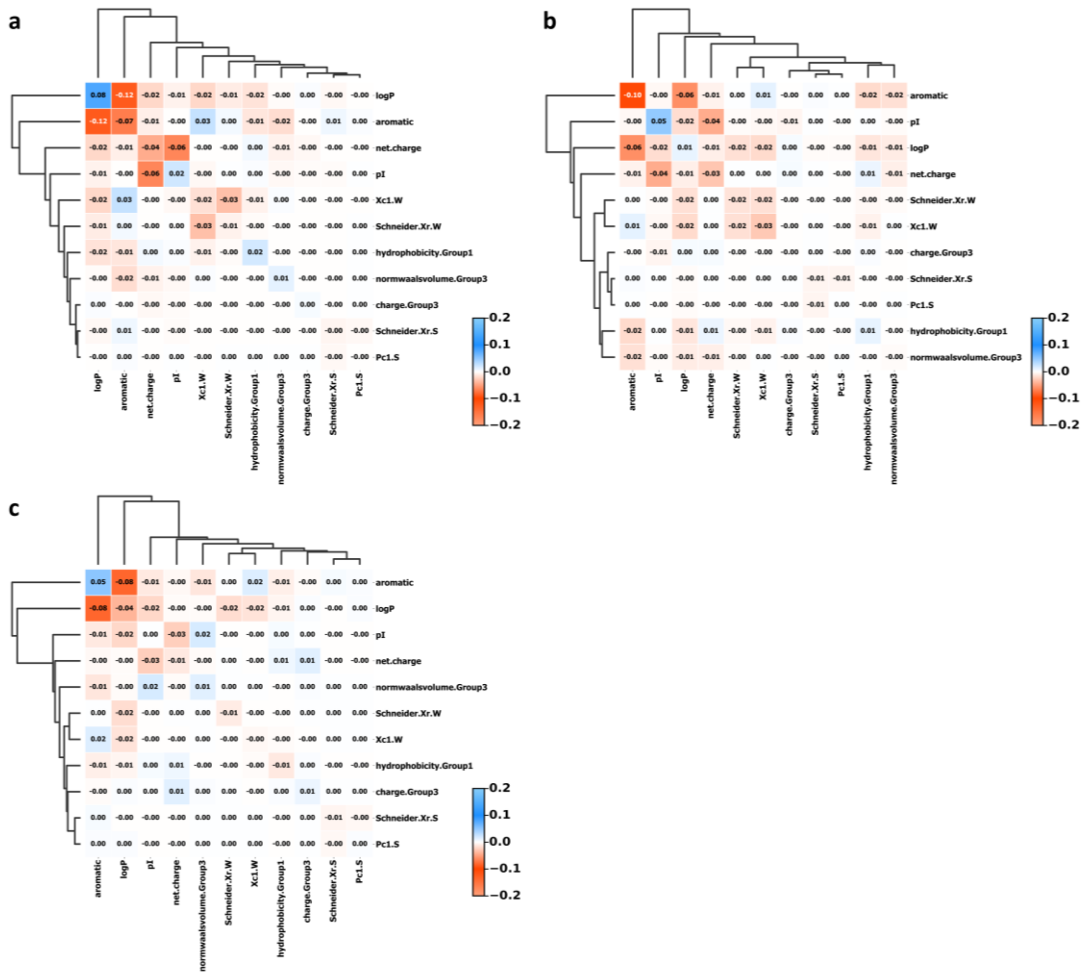

Figure S33. Heatmaps of SHAP interaction values for 11 features in Class 1. **a-c)** The non-diagonal elements quantitatively represent the strength and direction (positive in blue, negative in orange) of interaction effects between features, with diagonal elements replaced by the main effect values of individual features. The figures are arranged in the following order: LightGBM, XGBoost, and CatBoost.

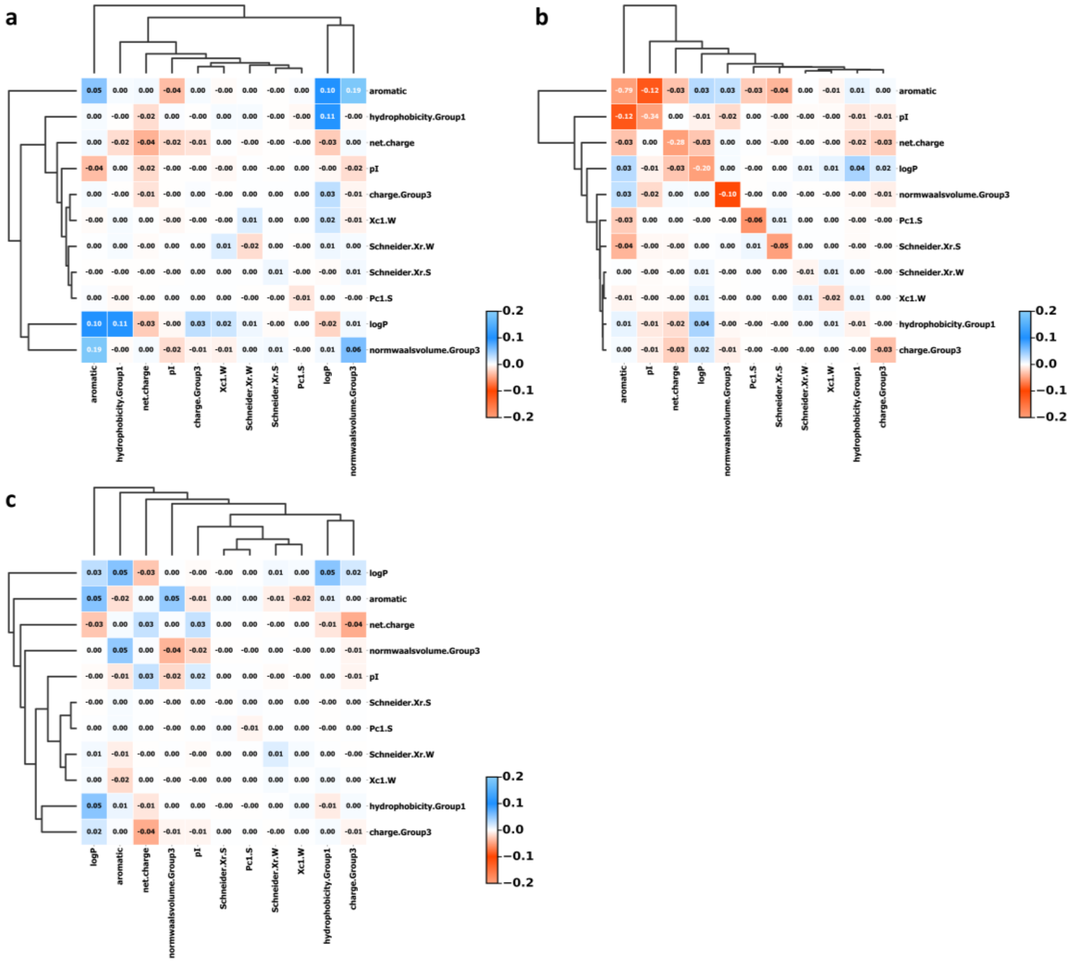

Figure S34. Heatmaps of SHAP interaction values for 11 features in Class 2. **a-c)** The non-diagonal elements quantitatively represent the strength and direction (positive in blue, negative in orange) of interaction effects between features, with diagonal elements replaced by the main effect values of individual features. The figures are arranged in the following order: LightGBM, XGBoost, and CatBoost.

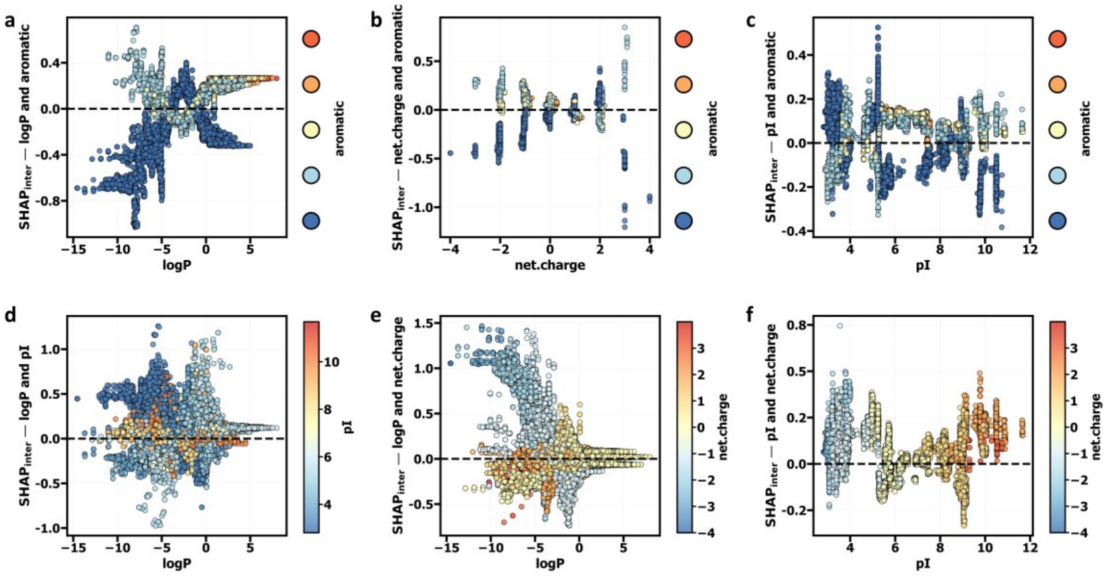

Figure S35. SHAP interaction effect dependence plots for the top 4 features (logP, aromatic, pI, net.charge) in Class 0 for the LightGBM model. **a-c)** SHAP interaction effect dependence plots for logP and aromatic (a), net.charge and aromatic (b), and pI and aromatic (c). The color gradient represents the interaction effect magnitude for the aromatic feature. **d-f)** SHAP interaction effect dependence plots for logP and pI (d), logP and net.charge (e), and pI and net.charge (f). The color gradient indicates the interaction effect magnitude for pI (d), net.charge (e), and net.charge (f), respectively. Black dashed lines indicate where SHAP effect values are zero, showing the direction and strength of feature interaction impacts on the model's predictions.

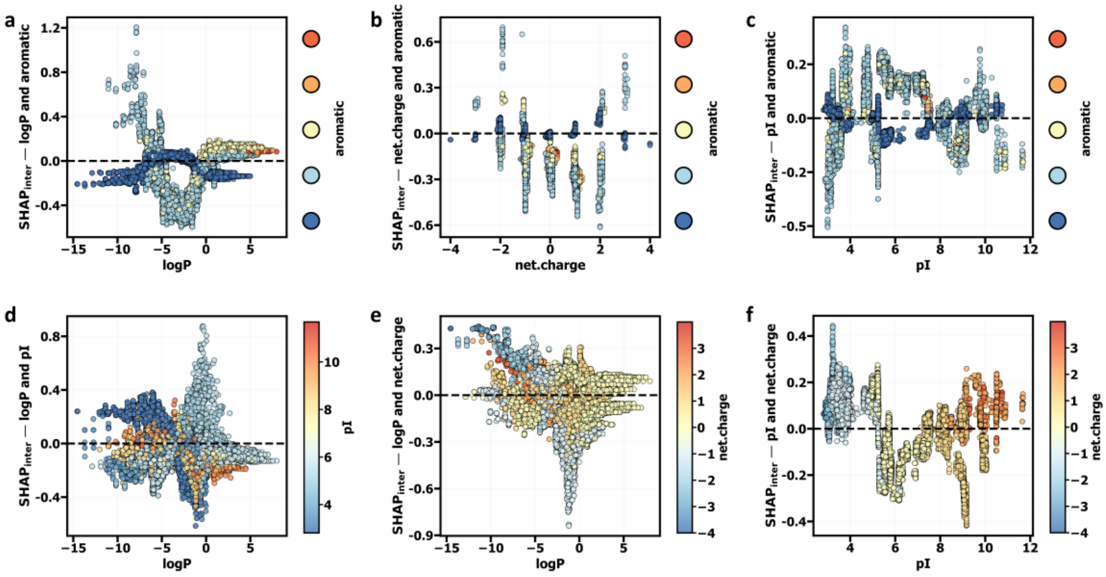

Figure S36. SHAP interaction effect dependence plots for the top 4 features (logP, aromatic, pI, net.charge) in Class 0 for the XGboost model. **a-c)** SHAP interaction effect dependence plots for logP and aromatic (a), net.charge and aromatic (b), and pI and aromatic (c). The color gradient represents the interaction effect magnitude for the aromatic feature. **d-f)** SHAP interaction effect dependence plots for logP and pI (d), logP and net.charge (e), and pI and net.charge (f). The color gradient indicates the interaction effect magnitude for pI (d), net.charge (e), and net.charge (f), respectively. Black dashed lines indicate where SHAP effect values are zero, showing the direction and strength of feature interaction impacts on the model's predictions.

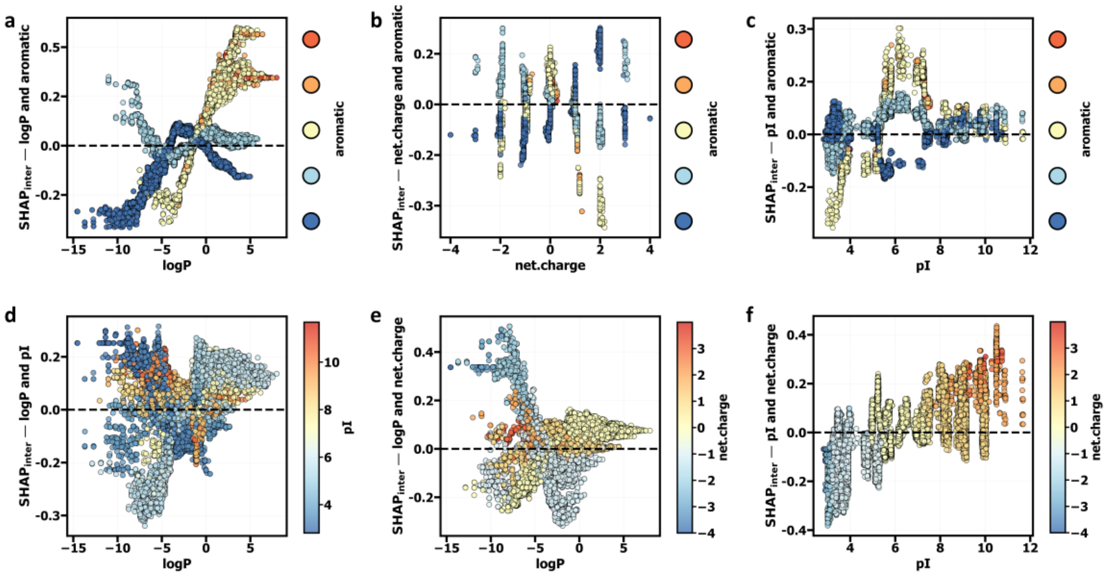

Figure S37. SHAP interaction effect dependence plots for the top 4 features (logP, aromatic, pI, net.charge) in Class 0 for the CatBoost model. **a-c)** SHAP interaction effect dependence plots for logP and aromatic (a), net.charge and aromatic (b), and pI and aromatic (c). The color gradient represents the interaction effect magnitude for the aromatic feature. **d-f)** SHAP interaction effect dependence plots for logP and pI (d), logP and net.charge (e), and pI and net.charge (f). The color gradient indicates the interaction effect magnitude for pI (d), net.charge (e), and net.charge (f), respectively. Black dashed lines indicate where SHAP effect values are zero, showing the direction and strength of feature interaction impacts on the model's predictions.

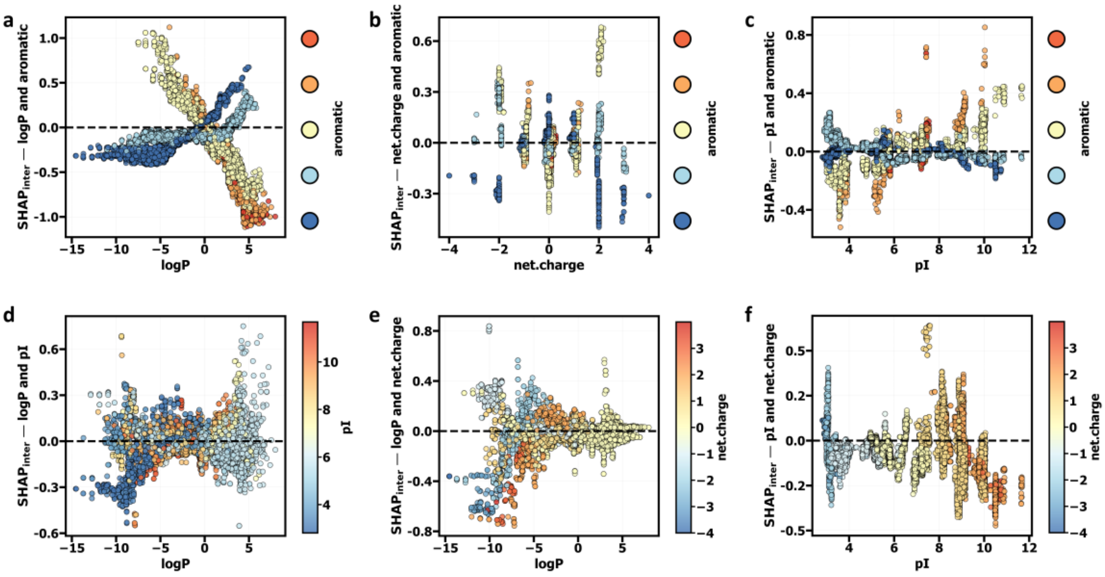

Figure S38. SHAP interaction effect dependence plots for the top 4 features (logP, aromatic, pI, net.charge) in Class 1 for the LightGBM model. **a-c)** SHAP interaction effect dependence plots for logP and aromatic (a), net.charge and aromatic (b), and pI and aromatic (c). The color gradient represents the interaction effect magnitude for the aromatic feature. **d-f)** SHAP interaction effect dependence plots for logP and pI (d), logP and net.charge (e), and pI and net.charge (f). The color gradient indicates the interaction effect magnitude for pI (d), net.charge (e), and net.charge (f), respectively. Black dashed lines indicate where SHAP effect values are zero, showing the direction and strength of feature interaction impacts on the model's predictions.

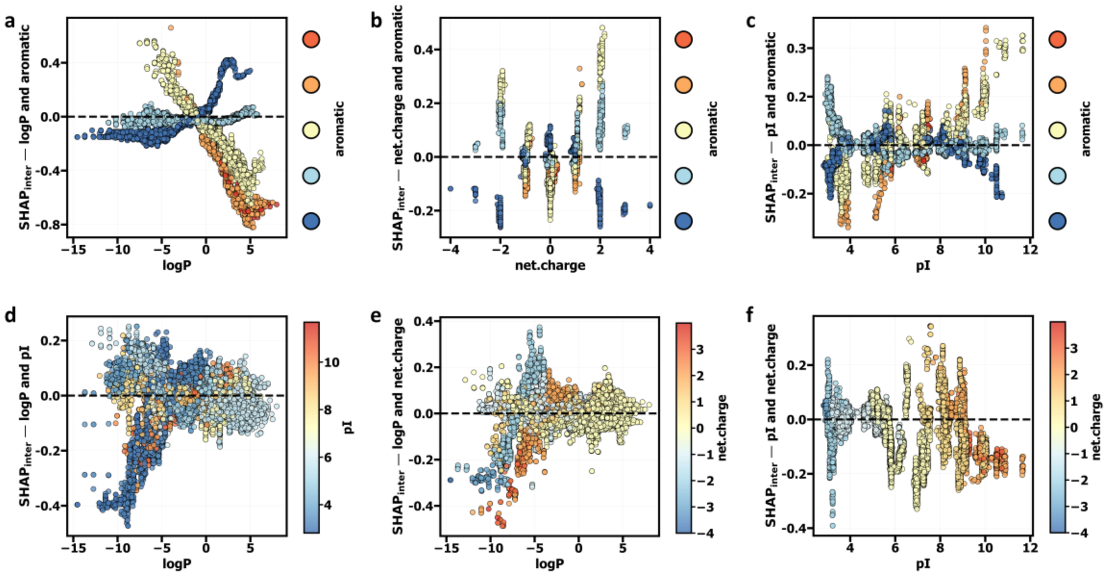

Figure S39. SHAP interaction effect dependence plots for the top 4 features (logP, aromatic, pI, net.charge) in Class 1 for the XGBoost model. **a-c)** SHAP interaction effect dependence plots for logP and aromatic (a), net.charge and aromatic (b), and pI and aromatic (c). The color gradient represents the interaction effect magnitude for the aromatic feature. **d-f)** SHAP interaction effect dependence plots for logP and pI (d), logP and net.charge (e), and pI and net.charge (f). The color gradient indicates the interaction effect magnitude for pI (d), net.charge (e), and net.charge (f), respectively. Black dashed lines indicate where SHAP effect values are zero, showing the direction and strength of feature interaction impacts on the model's predictions.

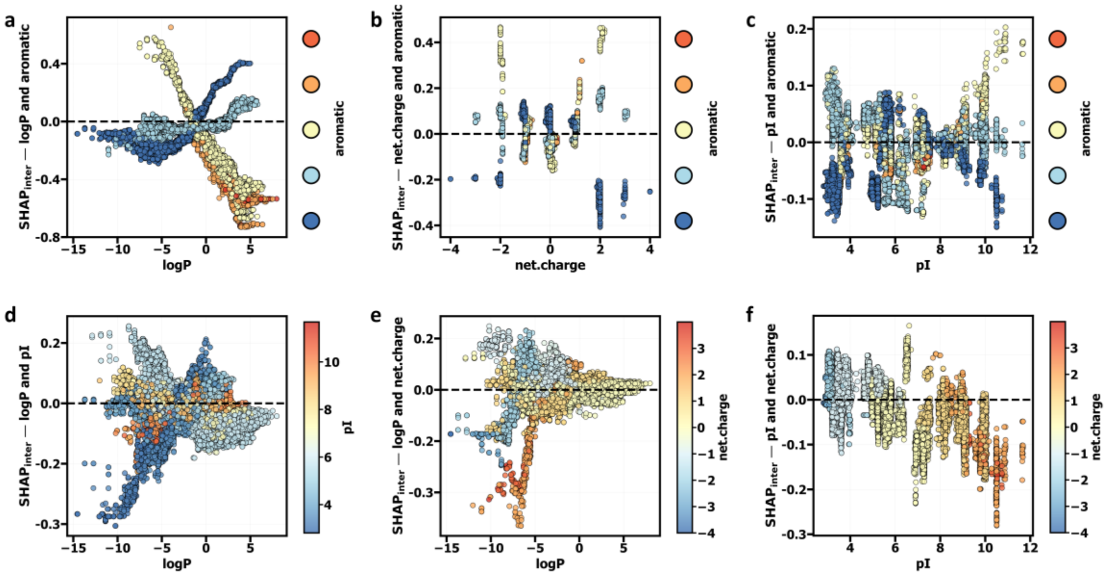

Figure S40. SHAP interaction effect dependence plots for the top 4 features (logP, aromatic, pI, net.charge) in Class 1 for the CatBoost model. **a-c)** SHAP interaction effect dependence plots for logP and aromatic (a), net.charge and aromatic (b), and pI and aromatic (c). The color gradient represents the interaction effect magnitude for the aromatic feature. **d-f)** SHAP interaction effect dependence plots for logP and pI (d), logP and net.charge (e), and pI and net.charge (f). The color gradient indicates the interaction effect magnitude for pI (d), net.charge (e), and net.charge (f), respectively. Black dashed lines indicate where SHAP effect values are zero, showing the direction and strength of feature interaction impacts on the model's predictions.

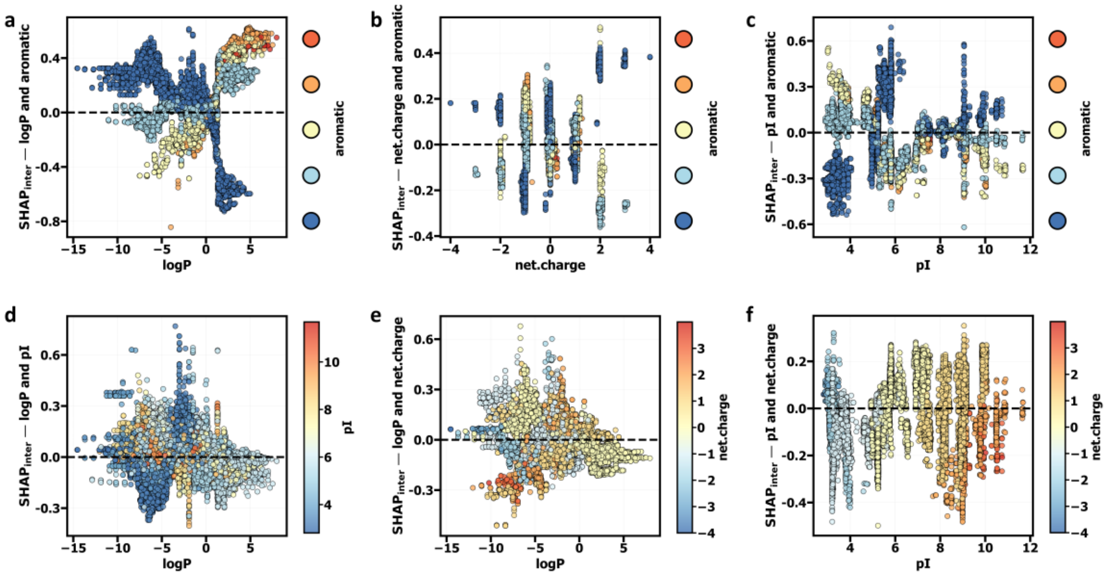

Figure S41. SHAP interaction effect dependence plots for the top 4 features (logP, aromatic, pI, net.charge) in Class 2 for the LightGBM model. **a-c)** SHAP interaction effect dependence plots for logP and aromatic (a), net.charge and aromatic (b), and pI and aromatic (c). The color gradient represents the interaction effect magnitude for the aromatic feature. **d-f)** SHAP interaction effect dependence plots for logP and pI (d), logP and net.charge (e), and pI and net.charge (f). The color gradient indicates the interaction effect magnitude for pI (d), net.charge (e), and net.charge (f), respectively. Black dashed lines indicate where SHAP effect values are zero, showing the direction and strength of feature interaction impacts on the model's predictions.

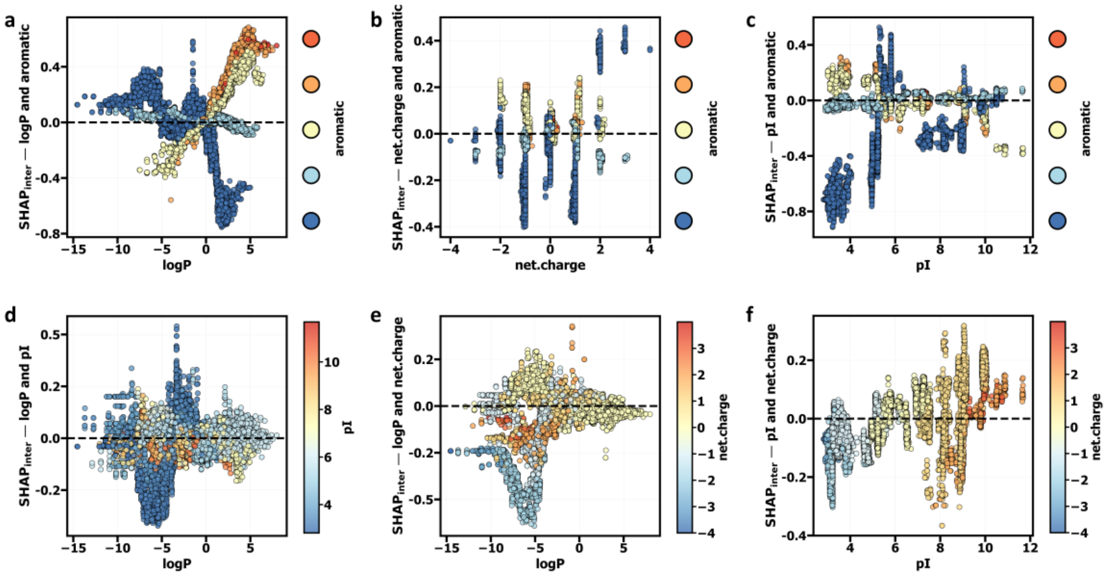

Figure S42. SHAP interaction effect dependence plots for the top 4 features (logP, aromatic, pI, net.charge) in Class 2 for the XGBoost model. **a-c)** SHAP interaction effect dependence plots for logP and aromatic (a), net.charge and aromatic (b), and pI and aromatic (c). The color gradient represents the interaction effect magnitude for the aromatic feature. **d-f)** SHAP interaction effect dependence plots for logP and pI (d), logP and net.charge (e), and pI and net.charge (f). The color gradient indicates the interaction effect magnitude for pI (d), net.charge (e), and net.charge (f), respectively. Black dashed lines indicate where SHAP effect values are zero, showing the direction and strength of feature interaction impacts on the model's predictions.

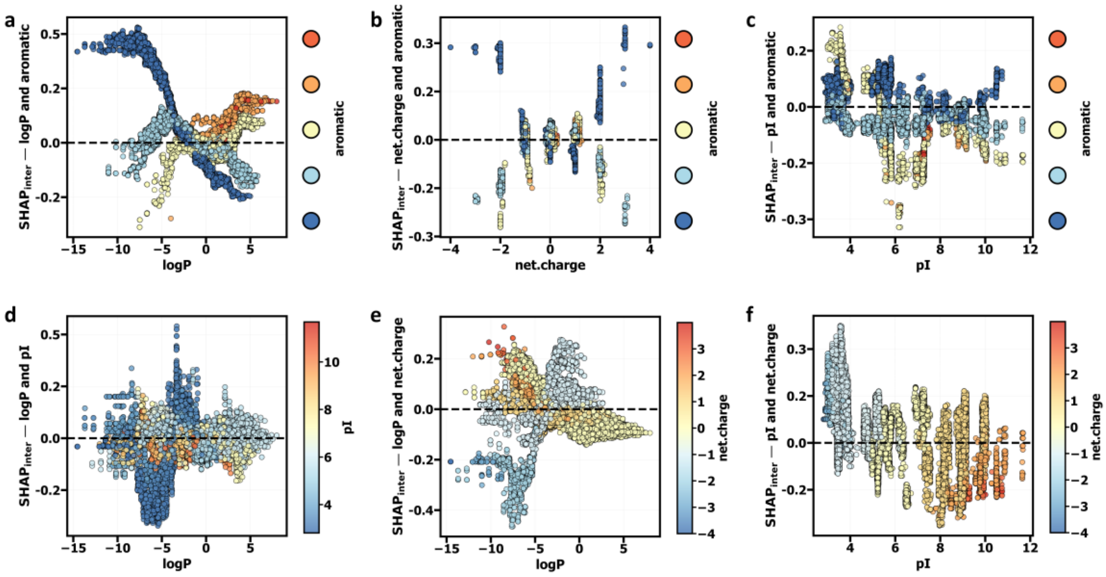

Figure S43. SHAP interaction effect dependence plots for the top 4 features (logP, aromatic, pI, net.charge) in Class 1 for the CatBoost model. **a-c)** SHAP interaction effect dependence plots for logP and aromatic (a), net.charge and aromatic (b), and pI and aromatic (c). The color gradient represents the interaction effect magnitude for the aromatic feature. **d-f)** SHAP interaction effect dependence plots for logP and pI (d), logP and net.charge (e), and pI and net.charge (f). The color gradient indicates the interaction effect magnitude for pI (d), net.charge (e), and net.charge (f), respectively. Black dashed lines indicate where SHAP effect values are zero, showing the direction and strength of feature interaction impacts on the model's predictions.

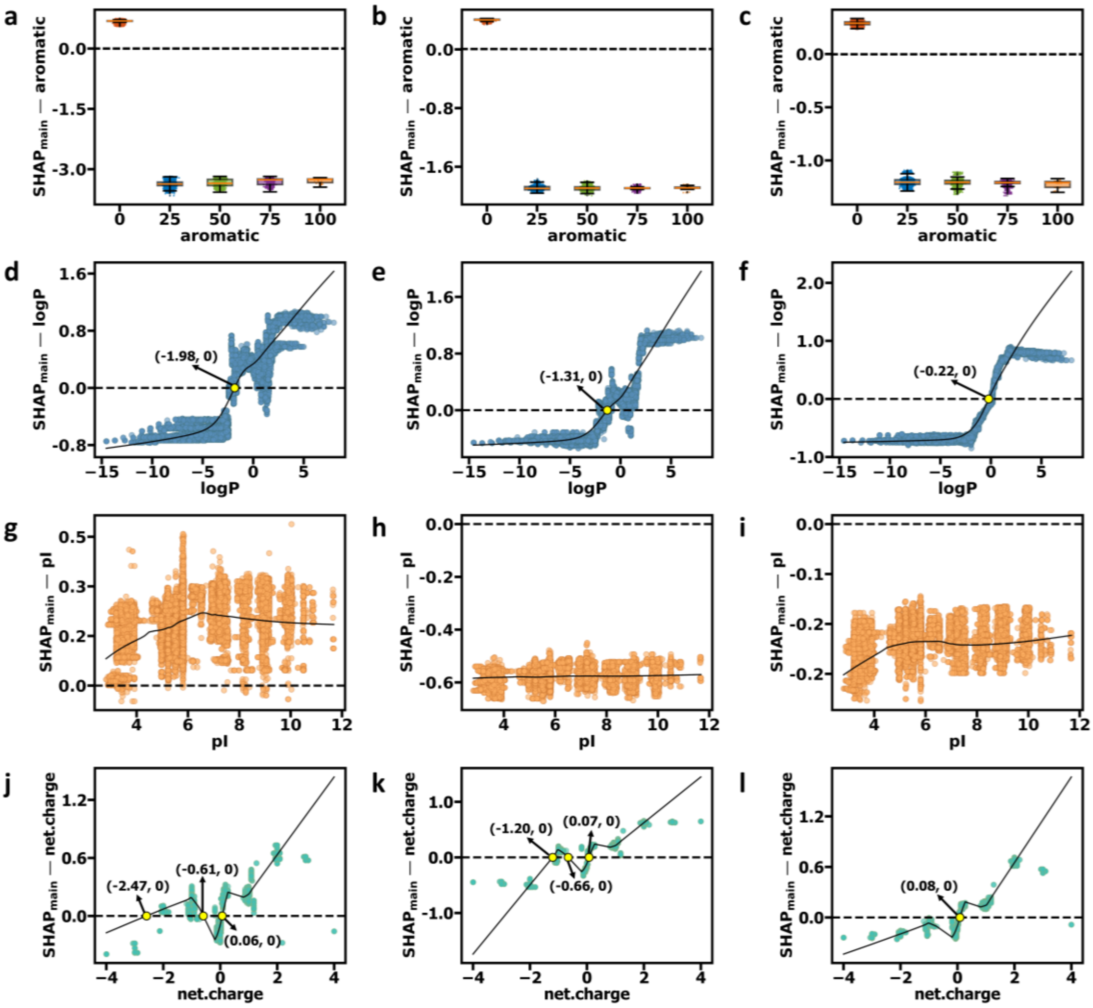

Figure S44. Main effect SHAP dependence plots for top features in Class 1 of AP. a-c) SHAP main effect dependence plots for the aromatic property on Class 1 results in the LightGBM, XGBoost, and CatBoost models (from left to right). d-f) SHAP main effect dependence plots for the logP feature on Class 1 results in the LightGBM, XGBoost, and CatBoost models (from left to right). g-i) SHAP main effect dependence plots for the pI feature on Class 1 results in the LightGBM, XGBoost, and CatBoost models (from left to right). j-l) SHAP main effect dependence plots for the net charge feature on Class 1 results. The black LOWESS (Locally Weighted Scatterplot Smoothing) curve in each plot depicts the overall trend between the feature values and their corresponding SHAP main effect values.

Section 2: Supplementary Tables

Table S1. Hyperparameter search space for random forest and support vector machine models.

Model	Hyperparameter	Possible Values
**Random Forest (RF)**	n_estimators	[100, 200, 300, 400, 500, 600, 700, 800, 900, 1000]
	max_features	['sqrt', 'log2']
	max_depth	[None, 10, 20, 30, 40, 50]
	min_samples_split	[2, 5, 10]
	min_samples_leaf	[1, 2, 4]
	bootstrap	[True, False]
	min_weight_fraction_leaf	[0.0, 0.1, 0.2]
	max_leaf_nodes	[None, 10, 20, 30]
	min_impurity_decrease	[0.0, 0.01, 0.02]
**Support Vector Machine (SVM)**	kernel	['linear', 'poly', 'rbf']
	C	Uniform(0.001, 100)
	degree	randint(1, 5)
	gamma	['scale', 'auto']

Table S2. Hyperparameter search space for tree-based models (XGBoost, LightGBM, CatBoost).

Model	Hyperparameter	Possible Values
**XGBoost**	number of trees	[100, 200, 300, 400, 500]
	tree depth	[3, 4, 5, 6, 7]
	learning rate	[0.01, 0.02, 0.05, 0.1]
**LightGBM**	number of trees	[100, 200, 300, 400, 500]
	tree depth	[3, 4, 5, 6, 7]
	learning rate	[0.01, 0.02, 0.05, 0.1]
**CatBoost**	number of trees	[100, 200, 300]
	tree depth	[3, 4, 5, 6, 7]
	learning rate	[0.01, 0.02, 0.05, 0.1]

Table S3. Performance (AP prediction) analysis of different machine learning algorithms (RF, SVM, Transformer) on a **fixed testing dataset of 10,000 samples**. This table summarizes the training sizes at which the key performance metrics (R^2, MAE, MSE, RMSE) reach their best performance points and inflection points during the learning curve.

algorithm	train_size	value	point_type	properties	metric
**RF**	15000	0.821	Best	AP	R2_TEST
**RF**	2500	0.741	Inflection	AP	R2_TEST
**SVM**	15000	0.901	Best	AP	R2_TEST
**SVM**	10500	0.864	Inflection	AP	R2_TEST
**Transformer**	16000	0.945	Best	AP	R2_TEST
**Transformer**	3500	0.910	Inflection	AP	R2_TEST
**RF**	15000	0.077	Best	AP	MAE_TEST
**RF**	1500	0.107	Inflection	AP	MAE_TEST
**SVM**	16000	0.061	Best	AP	MAE_TEST
**SVM**	10500	0.072	Inflection	AP	MAE_TEST
**Transformer**	16000	0.045	Best	AP	MAE_TEST
**Transformer**	3500	0.058	Inflection	AP	MAE_TEST
**RF**	15000	0.104	Best	AP	RMSE_TEST
**RF**	2500	0.126	Inflection	AP	RMSE_TEST
**SVM**	15000	0.078	Best	AP	RMSE_TEST
**SVM**	10500	0.091	Inflection	AP	RMSE_TEST
**Transformer**	16000	0.058	Best	AP	RMSE_TEST
**Transformer**	3500	0.074	Inflection	AP	RMSE_TEST
**RF**	15000	0.011	Best	AP	MSE_TEST
**RF**	2500	0.016	Inflection	AP	MSE_TEST
**SVM**	15000	0.006	Best	AP	MSE_TEST
**SVM**	10500	0.008	Inflection	AP	MSE_TEST
**Transformer**	13500	0.003	Best	AP	MSE_TEST
**Transformer**	14000	0.003	Best	AP	MSE_TEST
**Transformer**	14500	0.003	Best	AP	MSE_TEST
**Transformer**	15000	0.003	Best	AP	MSE_TEST
**Transformer**	15500	0.003	Best	AP	MSE_TEST
**Transformer**	16000	0.003	Best	AP	MSE_TEST
**Transformer**	3500	0.005	Inflection	AP	MSE_TEST
**RF**	16000	0.929	Best	logP	R2_TEST
**RF**	2000	0.853	Inflection	logP	R2_TEST
**SVM**	16000	1.000	Best	logP	R2_TEST
**SVM**	8500	1.000	Inflection	logP	R2_TEST
**Transformer**	9000	0.998	Best	logP	R2_TEST
**Transformer**	4500	0.991	Inflection	logP	R2_TEST
**RF**	16000	0.610	Best	logP	MAE_TEST
**RF**	2000	0.903	Inflection	logP	MAE_TEST
**SVM**	11000	0.039	Best	logP	MAE_TEST
**SVM**	6000	0.040	Inflection	logP	MAE_TEST
**Transformer**	9000	0.099	Best	logP	MAE_TEST
**Transformer**	4500	0.221	Inflection	logP	MAE_TEST

**RF**	16000	0.826	Best	logP	RMSE_TEST
**RF**	2000	1.191	Inflection	logP	RMSE_TEST
**SVM**	16000	0.050	Best	logP	RMSE_TEST
**SVM**	8500	0.061	Inflection	logP	RMSE_TEST
**Transformer**	9000	0.131	Best	logP	RMSE_TEST
**Transformer**	4500	0.289	Inflection	logP	RMSE_TEST
**RF**	16000	0.682	Best	logP	MSE_TEST
**RF**	2000	1.418	Inflection	logP	MSE_TEST
**SVM**	16000	0.002	Best	logP	MSE_TEST
**SVM**	8500	0.004	Inflection	logP	MSE_TEST
**Transformer**	9000	0.017	Best	logP	MSE_TEST
**Transformer**	4500	0.084	Inflection	logP	MSE_TEST
**RF**	16000	0.967	Best	pI	R2_TEST
**RF**	2500	0.932	Inflection	pI	R2_TEST
**SVM**	16000	0.974	Best	pI	R2_TEST
**SVM**	2000	0.935	Inflection	pI	R2_TEST
**Transformer**	15000	0.993	Best	pI	R2_TEST
**Transformer**	4500	0.985	Inflection	pI	R2_TEST
**RF**	15500	0.268	Best	pI	MAE_TEST
**RF**	2500	0.395	Inflection	pI	MAE_TEST
**SVM**	15500	0.241	Best	pI	MAE_TEST
**SVM**	1500	0.445	Inflection	pI	MAE_TEST
**Transformer**	15000	0.111	Best	pI	MAE_TEST
**Transformer**	4500	0.177	Inflection	pI	MAE_TEST
**RF**	16000	0.366	Best	pI	RMSE_TEST
**RF**	2500	0.524	Inflection	pI	RMSE_TEST
**SVM**	16000	0.321	Best	pI	RMSE_TEST
**SVM**	2000	0.511	Inflection	pI	RMSE_TEST
**Transformer**	15000	0.162	Best	pI	RMSE_TEST
**Transformer**	4500	0.244	Inflection	pI	RMSE_TEST
**RF**	16000	0.134	Best	pI	MSE_TEST
**RF**	2500	0.274	Inflection	pI	MSE_TEST
**SVM**	16000	0.103	Best	pI	MSE_TEST
**SVM**	2000	0.261	Inflection	pI	MSE_TEST
**Transformer**	15000	0.026	Best	pI	MSE_TEST
**Transformer**	4500	0.060	Inflection	pI	MSE_TEST

Table S4. Performance (AP prediction) analysis of different AI algorithms (RF, SVM, Transformer) on a **non-fixed testing dataset**. This table summarizes the training sizes at which the key performance metrics (R^2, MAE, MSE, RMSE) reach their best performance points and inflection points during the learning curve. The results are derived from evaluations on a varying test set, in contrast to the fixed testing dataset used in **Table 3**.

algorithm	train_size	value	point_type	properties	metric
**RF**	16000	0.821	Best	AP	R2_TEST
**RF**	2500	0.744	Inflection	AP	R2_TEST
**SVM**	15000	0.902	Best	AP	R2_TEST
**SVM**	10500	0.869	Inflection	AP	R2_TEST
**Transformer**	15500	0.946	Best	AP	R2_TEST
**Transformer**	3500	0.910	Inflection	AP	R2_TEST
**RF**	15500	0.079	Best	AP	MAE_TEST
**RF**	16000	0.079	Best	AP	MAE_TEST
**RF**	2500	0.096	Inflection	AP	MAE_TEST
**SVM**	15000	0.061	Best	AP	MAE_TEST
**SVM**	10500	0.071	Inflection	AP	MAE_TEST
**Transformer**	15500	0.045	Best	AP	MAE_TEST
**Transformer**	16000	0.045	Best	AP	MAE_TEST
**Transformer**	3500	0.059	Inflection	AP	MAE_TEST
**RF**	16000	0.106	Best	AP	RMSE_TEST
**RF**	2500	0.127	Inflection	AP	RMSE_TEST
**SVM**	15000	0.078	Best	AP	RMSE_TEST
**SVM**	10500	0.090	Inflection	AP	RMSE_TEST
**Transformer**	15500	0.058	Best	AP	RMSE_TEST
**Transformer**	3500	0.075	Inflection	AP	RMSE_TEST
**RF**	16000	0.011	Best	AP	MSE_TEST
**RF**	2500	0.016	Inflection	AP	MSE_TEST
**SVM**	15000	0.006	Best	AP	MSE_TEST
**SVM**	15500	0.006	Best	AP	MSE_TEST
**SVM**	16000	0.006	Best	AP	MSE_TEST
**SVM**	10500	0.008	Inflection	AP	MSE_TEST
**Transformer**	15000	0.003	Best	AP	MSE_TEST
**Transformer**	15500	0.003	Best	AP	MSE_TEST
**Transformer**	16000	0.003	Best	AP	MSE_TEST
**Transformer**	3500	0.006	Inflection	AP	MSE_TEST
**RF**	16000	0.930	Best	logP	R2_TEST
**RF**	2000	0.855	Inflection	logP	R2_TEST
**SVM**	16000	1.000	Best	logP	R2_TEST
**SVM**	8500	1.000	Inflection	logP	R2_TEST
**Transformer**	9000	0.998	Best	logP	R2_TEST
**Transformer**	4500	0.991	Inflection	logP	R2_TEST
**RF**	16000	0.615	Best	logP	MAE_TEST
**RF**	2000	0.906	Inflection	logP	MAE_TEST
**SVM**	11000	0.039	Best	logP	MAE_TEST
**SVM**	6000	0.040	Inflection	logP	MAE_TEST
**Transformer**	9000	0.099	Best	logP	MAE_TEST

**Transformer**	4500	0.222	Inflection	logP	MAE_TEST
**RF**	16000	0.827	Best	logP	RMSE_TEST
**RF**	2000	1.190	Inflection	logP	RMSE_TEST
**SVM**	16000	0.050	Best	logP	RMSE_TEST
**SVM**	8500	0.061	Inflection	logP	RMSE_TEST
**Transformer**	5500	0.124	Best	logP	RMSE_TEST
**Transformer**	4500	0.289	Inflection	logP	RMSE_TEST
**RF**	16000	0.684	Best	logP	MSE_TEST
**RF**	2000	1.415	Inflection	logP	MSE_TEST
**SVM**	16000	0.002	Best	logP	MSE_TEST
**SVM**	5500	0.004	Inflection	logP	MSE_TEST
**Transformer**	9000	0.017	Best	logP	MSE_TEST
**Transformer**	4500	0.084	Inflection	logP	MSE_TEST
**RF**	16000	0.967	Best	pI	R2_TEST
**RF**	2500	0.932	Inflection	pI	R2_TEST
**SVM**	16000	0.974	Best	pI	R2_TEST
**SVM**	2000	0.935	Inflection	pI	R2_TEST
**Transformer**	15000	0.994	Best	pI	R2_TEST
**Transformer**	4500	0.985	Inflection	pI	R2_TEST
**RF**	15500	0.266	Best	pI	MAE_TEST
**RF**	2500	0.391	Inflection	pI	MAE_TEST
**SVM**	16000	0.239	Best	pI	MAE_TEST
**SVM**	1500	0.442	Inflection	pI	MAE_TEST
**Transformer**	15000	0.110	Best	pI	MAE_TEST
**Transformer**	4500	0.174	Inflection	pI	MAE_TEST
**RF**	16000	0.362	Best	pI	RMSE_TEST
**RF**	2500	0.519	Inflection	pI	RMSE_TEST
**SVM**	16000	0.320	Best	pI	RMSE_TEST
**SVM**	2000	0.507	Inflection	pI	RMSE_TEST
**Transformer**	15000	0.161	Best	pI	RMSE_TEST
**Transformer**	4500	0.242	Inflection	pI	RMSE_TEST
**RF**	16000	0.131	Best	pI	MSE_TEST
**RF**	2500	0.269	Inflection	pI	MSE_TEST
**SVM**	16000	0.102	Best	pI	MSE_TEST
**SVM**	2000	0.257	Inflection	pI	MSE_TEST
**Transformer**	15000	0.026	Best	pI	MSE_TEST
**Transformer**	4500	0.058	Inflection	pI	MSE_TEST

Table S5. Cross-seed performance of the Transformer model for AP prediction (n=3,500).

Sample Size	Metric	Seed 42	Seed 123	Seed 456	Seed 789	Seed 999	Mean ± SD
**3,500**	R^2	0.9005	0.8909	0.8774	0.8942	0.9046	0.8935 ± 0.0094
**3,500**	MAE	0.0615	0.0636	0.0666	0.0627	0.0603	0.0629 ± 0.0021
**3,500**	RMSE	0.0790	0.0827	0.0876	0.0814	0.0773	0.0816 ± 0.0036

Table S6. Summary of Hamming distances between training and test sets across five training set sizes.

Training set size	Test set type	Test set size	Mean Hamming distance	Distance = 0 (%)	Distance = 1 (%)	Distance ≥ 2 (%)	Distance ≥ 3 (%)
**1,000**	Fixed	10,000	3.800 ± 0.436	0.00	0.05	99.95	98.60
**1,000**	Non-fixed	159,000	3.800 ± 0.436	0.00	0.05	99.95	98.60
**4,000**	Fixed	10,000	3.800 ± 0.436	0.00	0.05	99.95	98.60
**4,000**	Non-fixed	156,000	3.800 ± 0.436	0.00	0.05	99.95	98.60
**8,000**	Fixed	10,000	3.800 ± 0.436	0.00	0.04	99.96	98.60
**8,000**	Non-fixed	152,000	3.800 ± 0.436	0.00	0.05	99.95	98.60
**12,000**	Fixed	10,000	3.800 ± 0.435	0.00	0.05	99.95	98.61
**12,000**	Non-fixed	148,000	3.800 ± 0.436	0.00	0.05	99.95	98.61
**16,000**	Fixed	10,000	3.800 ± 0.436	0.00	0.05	99.95	98.60
**16,000**	Non-fixed	144,000	3.800 ± 0.436	0.00	0.05	99.95	98.59

Table S7. Bootstrap stability statistics of SHAP values for the LightGBM model.

Feature	Mean |SHAP|	Std |SHAP|	CV	Mean Rank	Rank Stability
**aromatic**	0.7414	0.0040	0.0054	1.00	√
**net.charge**	0.2277	0.0014	0.0059	2.23	√
**logP**	0.2267	0.0013	0.0056	2.77	√
**pI**	0.1846	0.0008	0.0044	4.00	√
**Xc1.W**	0.1701	0.0013	0.0075	5.00	√
**normwaalsvolume.Group3**	0.0819	0.0005	0.0056	6.00	√
**hydrophobicity.Group1**	0.0773	0.0004	0.0055	7.41	√
**Pc1.S**	0.0772	0.0006	0.0075	7.59	√
**Schneider.Xr.S**	0.0713	0.0007	0.0105	9.02	√
**Schneider.Xr.W**	0.0699	0.0006	0.0082	9.98	√
**charge.Group3**	0.0387	0.0003	0.0069	11.00	√

Note: The table reports the mean absolute SHAP value (Mean |SHAP|), its standard deviation (Std |SHAP|), the coefficient of variation (CV = Std |SHAP| / Mean |SHAP|), the mean rank of importance (1 = most important), and a binary indicator of rank stability (TRUE if the standard deviation of rank across bootstrap samples is less than 2). The average CV across all features is 0.0067, indicating exceptional stability of SHAP attributions.

Table S8. Performance evaluation of three machine learning models (XGBoost, LightGBM, CatBoost) after feature selection, where 11 key features were identified from an initial pool of 725 variables. The table presents various commonly performance metrics for each model, including accuracy, precision, recall, F1 score, ROC AUC, and PR AUC.

Model	Accuracy	Precision	Recall	F1 Score	ROC AUC	PR AUC	Parameters
**XGBoost**	0.879	0.879	0.879	0.878	0.969	0.934	'n_estimators': 400, 'max_depth': 6, 'learning_rate': 0.1
**LightGBM**	0.885	0.884	0.885	0.884	0.971	0.941	'n_estimators': 400, 'max_depth': 6, 'learning_rate': 0.1
**CatBoost**	0.866	0.865	0.866	0.864	0.962	0.91	'learning_rate': 0.1, 'iterations': 300, 'depth': 6

Section 3: Supplementary Methods

3.1 A mathematical framework for peptide sequence sampling and analysis

Next we will discuss on analysis method for the Question (iii) first and then come back to establish the optimality result on the UD for the Question (ii). We will employ RF as our primary analysis method after we sample the points and conduct MD experiments and AI predictions for obtaining the physicochemical properties. RF, originally proposed by the statistician, Leo Breimann(2), is one of widely used ML methods in AI and shown to be a consistent estimator of the function f. Compared to other ML methods, it carries a unique feature of a built-in cross-validation, and thus carries a minimal bias while the others possibly overfit the data to a larger bias. It is noted that in practice, RF usually induces a worse performance compared to other ML methods. However, as its overfitting is minimal, its performance should be closer to the theoretical bound. We will quote an important result on RF(3).

Lemma 2 (The Consistency of Random Forest(3)). Let $\{(X_i,Y_i)\}_{i=1}^{n}$ be i.i.d. observations where $X_i \in [0,1]^p$ and $\mathbb{E}[Y^2] < \infty$. Consider a random forest predictor $T_n(x)$ constructed as follows:

1. At each tree node, a split is chosen from m_{try} randomly selected coordinates

2. Trees are grown to maximal depth (until nodes contain k_n points)

3. The forest aggregates predictions from B_n such trees

Under these conditions:

1. (Breiman's Original RF) If $k_n \to \infty$ and $k_n/n \to 0$, then for almost all x,

$$\lim_{n\to\infty} \mathbb{E}\left[\left(T_n(X) - \mathbb{E}\left[Y[X]\right]\right)^2\right] = 0$$

Provided that:

· The feature space is partitioned uniformly at random

· $m_{try} = \lfloor p/3 \rfloor$ (default in many implementations)

It is noted that in the problem of evaluating k-peptides, $n \leq N$ is finite. However, as N grows, the histogram of f can be approximated by a distribution F as in this project, we will explore at what thresholds of sample size n or sampling fraction $sf = n/N$, selected analysis methods reach a convergence.\medskip

Lastly we will establish the optimality of UD in this study, under the mean square error, $\mathbb{E}\left[\left(\hat{f}\left(x|X(D),Y(D)\right) - Y|X = x\right)^2\right]$ with $X(D), Y(D)$being the design matrix and observed responses from the Design D, and utilizing Lemmas 1 and 2.

Theorem 1 (The Optimality of the Uniform Design). For a consistent estimator $\hat{f}$, if the function f is totally unknown and n is sufficiently large, then the

Uniform design will yield the predictor with lowest MSE among all possible designs.

Proof: We will start with a decomposition of MSE:

$$MSE(D) = \mathbb{E}\big(\hat{f}\big(x|X(D), Y(D)\big) - y|X = x\big)^2 = \mathbb{E}\big(\hat{f}(x|X(D), y(D)) - Y\big)^2$$
$$= \mathbb{E}(\hat{f}(x|X(D), y(D)) - f(X))^2 + \sigma^2$$

To establish the result, we will then convert the problem on the discrete space, S to the one on the unit hypercube, $[0, 1]^s$. Consider a q-equal-interval partition of $[0, 1]$, i.e., one can map q amino acid into $0, \frac{1}{q}, \cdots, \frac{q-1}{q}$. Then one can show that those two problems are equivalent via the following construction.

For any UD on $S = q^s$, one can construct a UD_1 on $[0, 1]^s$ by setting $UD_1(i) = \frac{i}{2q}$, if the ith amino acid is in the UD. That is, suppose $(i_1, i_2, \cdots, i_s) \epsilon\ UD_0$, then $UD_1 = \left\{\left(\frac{i_1}{2q}, \frac{i_2}{2q}, \cdots, \frac{i_s}{2q}\right)\right\}$, which is uniform design on $[0, 1]^s$.

Vice versa: For any point $(j_1, \cdots, j_s) \in UD_1$, then $UD_0 = \{\lfloor 2q * j_1 \rfloor, \lfloor 2q * j_2 \rfloor, \cdots, \lfloor 2q * j_s \rfloor\}^s$ is a uniform design on $[0, 1]^s$, here $\lfloor t \rfloor$ is the floor of a number t.

In this way, the problem of evaluating UD on S can be converted to evaluating UD on $[0, 1]^s$ with f being extended into a continuous function on $[0, 1]^s$. Therefore, the distribution of Y can be extended into a continuous one F on $[0, 1]^s$. For a continuous distribution function F on $[0, 1]^s$, we have $F(U) = F$, where U is uniform. Combining it with Lemma 1, one can show that the conditions in Lemma 2 hold.

Then we will have the following on $[0, 1]^s$:

$$MSE(D) = \mathbb{E}\big(\hat{f}\big(x|X(D), y(D)\big) - Y|X = x\big)^2$$
$$= \mathbb{E}\left(\hat{f}\big(x|X(D), y(D)\big) - f(x)\right)^2 + \sigma^2 \to \sigma^2\ as\ n \to \infty\ and\ \frac{n}{N} \to f < 1.$$

We will finally show the final result:

$$MSE(D_1) = \mathbb{E}\left(\hat{f}\big(x|X(D_1), y(D_1)\big) - \mathbb{E}(Y|X)\right)^2$$
$$= \mathbb{E}\left(\hat{f}\big(x|X(D_1), y(D_1)\big) - f(X)\right)^2 + \sigma^2 \geq \sigma^2.$$

Therefore, when n is sufficiently large, $MSE(D_1) \geq MSE(D)$.

With the established equivalency, such a result hold for the problem on S.

Note that f needs to be assumed to be totally unknown. Otherwise, suppose f can be represented as $g \circ h$, where g is totally unknown and h is known. Then following the similar approach in the above proof, one can show that $h^{-1}(D)$ is asymptotically better than D in MSE.

Currently, there is no unbiased predictor of the MSE. Therefore, even though RF enjoys consistency, there are many methods that output it in terms of a predictor of MSE, e.g., sample MSE with k-fold cross-validation.

3.2 CGMD simulations

To obtain the AP values of tetrapeptides, we conduct high-throughput coarse-grained molecular dynamics (CGMD) simulations using the Martini force field version 2.1(4), which has been rigorously validated for its effectiveness and accuracy in assessing the aggregation of short peptides (5-7). This force field simplifies molecular representations while retaining critical interactions by grouping approximately four heavy atoms into a single “bead”, rendering it particularly suitable for systematic peptide screening. We perform a total of 160,000 independent CGMD simulations in an aqueous environment, each containing 120 identical CG-ed tetrapeptides and approximately 4,000 water beads, which corresponds to roughly 16,000 explicit water molecules at the all-atom level due to the coarse-graining mapping. The cubic simulation box dimensions are initially set at 8 × 8 × 8 nm^3, resulting in a peptide concentration of 0.39 mol/L and a bulk solvent density of approximately 1 g/cm3. All simulations are conducted under isothermal-isobaric (NpT) ensemble conditions with the temperature maintained at 300 K using the velocity-rescaling thermostat and the pressure maintained at 1 bar using the Parrinello-Rahman barostat. Each simulation runs for a total of 100 ns, equivalent to 4,000,000 integration steps with a time step of 25 fs. Trajectory coordinates are saved every 500 ps for analysis. The aggregation propensity for each sequence is calculated from the last 50 ns of the production trajectory as the normalized average number of inter-peptide contacts, defined when any two non-bonded beads from distinct peptides are within a cutoff distance of 0.6 nm.

4. Supplementary results

4.1 Validation of SHAP analysis robustness

To ensure the reliability of the SHAP interpretations presented in the main text, we performed a comprehensive assessment of their stability against potential confounding factors, such as feature correlation. The key validation analyses are summarized below and detailed in the accompanying figures and table.

4.1.1 Multi-model consensus and feature correlation.

Our SHAP conclusions are derived from a consensus across three independent tree-based models (XGBoost, LightGBM, CatBoost), whose architecture is inherently robust to multicollinearity. While some feature correlations were identified (**Figure S22**), they do not invalidate the core findings: the high correlation between pI and net.charge is chemically expected, and the other highly correlated pairs involve secondary descriptors not central to our mechanistic conclusions.

4.1.2 Bootstrap stability of SHAP values.

A bootstrap resampling analysis (100 iterations) on the LightGBM model demonstrated exceptional stability in SHAP attributions for the optimal aggregation class (Class 1). As shown in **Figure S21** and **Table S7**, the average coefficient of variation (CV) across all features was only **0.0067**, with all key features (aromatic, logP, pI, net.charge) exhibiting CVs < 0.01, far below common stability thresholds.

4.1.3 Concordance with model-agnostic metrics.

The ranking of feature importance obtained from SHAP showed excellent agreement with that derived from model-agnostic permutation importance, yielding a Spearman rank correlation of **0.936** (**Figure S23**). This strong correlation provides independent confirmation that the features highlighted by SHAP are indeed critical for model performance.

4.1.4 Comparison with Partial Dependence Plots (PDP).

PDP and ICE plots for the top features are provided in **Figure S24**. The plots for aromatic and logP align with the non-linear relationships identified by SHAP. The relatively flat PDP for pI underscores a key methodological insight: PDPs measure the average marginal effect, while SHAP captures conditional contributions within feature interactions. This explains why pI shows minimal isolated effect in PDPs but a significant interactive role with net.charge in SHAP analysis, which is consistent with the expected electrostatics of peptide aggregation.

Collectively, these validation steps confirm that the principal SHAP-driven insights the aromaticity threshold effect, the logP window effect, and the critical interplay between pI and net charge are robust and reliable.

4.2 SHAP main effect analysis for key features in class 1 prediction

4.2.1 Aromaticity

Upon further isolating the $SHAP_{main}$ effect of the aromatic feature (**Figure S44a-c**), we discern a more precise influence pattern: the aromatic feature exhibits a positive impact on Class 1 prediction only in the absence of aromatic residues. However, the introduction of even a single aromatic residue results in a negative effect. This finding refines the prior threshold hypothesis, indicating that the influence of aromatic residues may be highly contingent upon the molecular environment, including the positional effects of aromatic residue sequence(8), the characteristics of adjacent amino acid residues(9), and the overall conformation of the peptide chain(10). Notably, while the presence of aromatic residues obstructs the formation of Class 1 aggregates, it positively contributes to a stronger aggregation tendency associated with Class 2 (**Figure S27**a–c and **Figure S29**a–c). This phenomenon may arise from the substantial modification of the peptide's physicochemical properties by aromatic residues through phi-phi stacking interactions, thereby influencing the self-assembly behavior.

4.2.2 logP

Further analysis of the SHAP main value dependence plot for logP in class 1 reveals that when logP is below the intersection points (-1.98 for LightGBM, -1.31 for XGBoost, -0.22 for CatBoost), the SHAP main effect value is negative. This implies that logP acts as an independent inhibitor to the model's prediction for Class 1. Nevertheless, as logP levels increase within the favorable range of -2 to 2, a positive correlation emerges between logP and SHAP main effect values. Despite the SHAP main effect value remaining consistently positive, the total SHAP value of logP tends to be negative, with the LOWESS curve positioned below zero (**Figure S44d-f**). This indicates that the positive independent effect of logP is counterbalanced by a substantial negative interaction effect, resulting in an overall negative contribution of logP to class 1 prediction.

4.2.3 pI

Analysis of the SHAP main effect values for pI characteristics reveals substantial differences among the three models. The LightGBM model indicates that pI consistently provides a positive independent contribution to Class 1 prediction across all ranges, whereas the XGBoost and CatBoost models show a negative independent contribution in all ranges (**Figure S44g–i**). This disparity not only highlights the intricate mode of action that pI characteristics may possess but also uncovers the robust interactions that may occur between pI and other attributes.

This notable difference may arise from several factors. First, the pI feature may exhibit complex distribution characteristics, such as multi-modality, which could lead the models to adopt different strategies in interpreting its effect. Second, inherent variations in feature splitting and tree structure construction among the models might result in divergent assessments of the main effect of pI. Third, the strong interaction between pI and other features, such as net charge or logP, may be handled differently by each model during the separation of main effects. Despite these variations, the general consistency of the SHAP dependence plots provides significant insights: maintaining the pI value within the inflection point range (between 5.7 and 6.3) appears crucial for designing optimal self-assembling peptide sequences. Within this range, the peptide chain can achieve sufficient solubility while efficiently facilitating molecular interactions, thereby enhancing the formation of stable self-assembled structures.

4.2.4 Net Charge

Regarding the SHAP main effect values for net charge, we observe that for most scatter points, the SHAP main effect values are above zero, further demonstrating the independent positive effect of high net charge on Class 1 (**Figure S44j-l**). The observed discrepancies indicate that the interactive effect between net charge and other features considerably influences the overall effect size, resulting in a negative impact on the total SHAP value. Thus, it is crucial to investigate how the interplay between net charge and

other characteristics affects these patterns, especially within the high net charge spectrum.

Reference

1. Fang K-T. The Uniform Design: Application of Number-Theoretic Methods in Experimental Design. Acta Math Appl Sin. 1980;3.

2. Breiman L. Random Forests. Machine Learning. 2001;45:5-32.

3. Scornet E, Biau G, Vert J-P. Consistency of Random Forests. The Annals of Statistics. 2014;43.

4. Marrink SJ, Risselada HJ, Yefimov S, Tieleman DP, de Vries AH. The MARTINI Force Field: Coarse Grained Model for Biomolecular Simulations. The Journal of Physical Chemistry B. 2007;111(27):7812-24.

5. Wang J, Liu Z, Zhao S, Zhang Y, Xu T, Li SZ, et al. Aggregation Rules of Short Peptides. JACS Au. 2024.

6. Wang J, Liu Z, Zhao S, Xu T, Wang H, Li SZ, et al. Deep Learning Empowers the Discovery of Self-Assembling Peptides with Over 10 Trillion Sequences. Advanced Science. 2023;10(31).

7. Xu T, Wang J, Zhao S, Chen D, Zhang H, Fang Y, et al. Accelerating the prediction and discovery of peptide hydrogels with human-in-the-loop. Nature Communications. 2023;14(1).

8. Baldwin RL, Rose GD. Molten globules, entropy-driven conformational change and protein folding. Current opinion in structural biology. 2013;23 1:4-10.

9. Finkelstein A, Ptitsyn OB. Protein Physics: A Course of Lectures: Second, Updated and Extended Edition2016. 1-508 p.

10. Krause E, Bienert M, Schmieder P, Wenschuh H. The Helix-Destabilizing Propensity Scale of d-Amino Acids: The Influence of Side Chain Steric Effects. Journal of the American Chemical Society. 2000;122(20):4865-70.
